# Structure-aware fatigue modeling in foot deformities: A digital health framework for tissue-specific running injury risk prediction using multi-modal data

**DOI:** 10.1371/journal.pdig.0001537

**Published:** 2026-07-10

**Authors:** Zhifeng Zhou, Huiyu Zhou, Datao Xu, Bas Van Hooren, Yi Yuan, Tianle Jie, Xiangli Gao, Xiuye Qu, Zanni Zhang, Zixiang Gao, Liangliang Xiang, Yaodong Gu

**Affiliations:** 1 Faculty of Sports Science, Ningbo University, Ningbo, China; 2 Department of Nutrition and Movement Sciences, NUTRIM Institute of Nutrition and Translational Research in Metabolism, Maastricht University Medical Centre+, Universiteitssingel 50, Maastricht, The Netherlands; 3 Research Academy of Medicine Combining Sports, Ningbo No. 2 Hospital, Ningbo, China; 4 Human Performance Laboratory, Faculty of Kinesiology, University of Calgary, Calgary, Alberta, Canada; 5 The Doctoral School on Safety and Security Sciences, Óbuda University, Budapest, Hungary; 6 Research Center for Molecular Exercise Science, Hungarian University of Sports Science, Budapest, Hungary; 7 KTH Move Ability Lab, Department of Engineering Mechanics, KTH Royal Institute of Technology, Stockholm, Sweden; Northumbria University, UNITED KINGDOM OF GREAT BRITAIN AND NORTHERN IRELAND

## Abstract

Hallux Valgus (HV) is a common deformity in runners that reflects altered foot morphology and thereby redistributes mechanical loads along the lower limb, possibly increasing injury risk. However, the biomechanical consequences of this deformity on loading at common running injury locations, and the resulting fatigue-failure injury risk remain undocumented. This study integrated gait analysis with subject-specific musculoskeletal modelling to estimate Achilles tendon, plantar fascia, patellofemoral, and tibial loading and probability of fatigue-failure during running at 12 km/h in a cohort of 26 runners with HV and 26 healthy controls. HV runners exhibited pronounced structural deviation, reflected by increased hallux valgus and intermetatarsal angles, and elevated Foot Posture Index scores. During running, HV runners showed reduced ankle and metatarsophalangeal range of motions and increased peak joint moments (ankle p = 0.004; MTP p = 0.003). These biomechanical alterations were associated with higher Cumulative Load, Cumulative Damage, and Probability of Fatigue Failure, particularly in the Achilles tendon and plantar fascia, whereas tibial loading was largely similar to individuals without HV. To enable individualized prediction of biomechanical loading at the four injury locations, we trained deep learning models that used IMU input data (Model 1) or IMU plus foot structure data (Model 2). Model 1 (SSO–CNN–BiLSTM–HAM) accurately estimated loading indices (R² = 0.89–0.93), and Model 2 further enhanced predictive accuracy (R² = 0.94–0.97). Collectively, these findings suggest that HVA and foot posture index-related structural deviations increase lower-limb loading patterns and accelerate tissue fatigue-failure probability.

## Author summary

Foot deformities are common in the general population, and hallux valgus is one of the most prevalent conditions. Although often considered a mild structural alteration, hallux valgus can influence the leg loading patterns during daily movement and sports such as running. Over time, these altered loading patterns may contribute to increased mechanical demand on key musculoskeletal tissues, such as the Achilles tendon and plantar fascia, potentially elevating the risk of overuse-related injury. However, because the biomechanical consequences of foot deformities remain largely undocumented, we quantified the biomechanical alterations in loading at common running injury locations in individuals with hallux valgus during running.

Further, the assessment of tissue-specific fatigue-failure processes typically requires laboratory-based biomechanical measurements, which limits accessibility for wider use. To address this challenge, as a secondary aim, we developed a digital health framework that integrates wearable inertial sensor data with structure-aware fatigue modeling to estimate tissue-specific fatigue-failure risk in individuals with and without foot deformities. The developed model demonstrated strong performance in predicting fatigue-related outcomes using wearable-derived signals, suggesting potential for non-invasive and scalable injury risk estimation based on wearable data.

## 1. Introduction

Running is a popular exercise owing to its ability to enhance cardiovascular fitness, overall physical performance, coordination, and muscular endurance. However, it is also associated with a high risk of injury, with approximately 40% of runners experiencing various sports-related injuries each year [1,2]. Most running-related injuries are classified as overuse injuries, arising from an imbalance between repetitive mechanical loading and the body’s capacity for recovery and adaptation [3]. Factors such as individual anthropometrics and structural deformities can substantially influence biomechanical loading patterns during running. For example, body mass index (BMI), limb alignment, leg length discrepancies, and joint morphology can all alter load distribution across tissues and contribute to injury risk [4,5].

Among various structural deformities, hallux valgus (HV) is particularly common in runners and is associated with altered first metatarsophalangeal joint alignment, increased foot pronation, and excessive external rotation of the lower extremity during gait and running [4–6]. Studies have shown that HV is associated with increased toe-out gait patterns, greater rearfoot eversion, and restricted dorsiflexion at both the ankle and first metatarsophalangeal joint (MTPJ), all of which may contribute to abnormal forefoot loading and increased abduction stress across the hallux during gait [4,7]. In addition, HV-related muscle imbalance around the first MTPJ impairs dynamic arch stability and healthy pronation, thereby increasing mechanical loading on the ankle and midfoot joints as well as tensile stress on the surrounding soft tissues during gait [8]. These biomechanical disturbances contribute to the development of plantar fasciitis, tibial stress fractures, and ankle sprains [6,9]. Despite these clinical implications, few studies have examined how HV alters tissue-level fatigue mechanisms during running [4,10].

Advancements in biomechanical modelling however now allow more accurate estimations of the biomechanical loading experienced by various tissues during movements such as running [11–14]. These models enable researchers to move beyond traditional kinematic and external kinetic analyses by facilitating the estimation of musculoskeletal loading at common injury sites. Furthermore, they can be refined to incorporate the non-linear mechanical properties of tendons and ligaments, thereby accounting for strain rate–dependent effects [15,16]. The load calculated on a per-step basis with these models can subsequently be aggregated to estimate cumulative tissue loading over a given running distance [17]. Further, the concept of cumulative load (CL) can be further extended by accounting for the nonlinear relationship between load magnitude and tissue fatigue life [18–20]. Specifically, ex vivo and in vitro studies have documented that higher loads contribute disproportionately more to damage accumulation and thus tissue fatigue life than lower loads [21–23]. This relationship is typically modelled by exponentially weighing the loading values, resulting in a cumulative damage (CD) estimate [24]. Further, due to variability in tissue failure at a given level of cumulative damage, probabilistic approaches such as Weibull-based models have been introduced to quantify the probability of fatigue failure (PFF) [25], thereby enabling a more robust characterization of injury risk by accounting for the stochastic nature of material strength and loading conditions. Collectively, the CL, CD, and PFF indices therefore provide a multi-dimensional representation of the biomechanical loading in relation to injury risk and may as such be used to better understand the effect of foot deformations on running injury risk [26–30].

While a better understanding of the effect of foot deformations on running biomechanical loading and injury risk is useful, the development of practical models that leverage such information, for example using wearable-derived data and simple anthropometric measures to estimate injury risk in real-world settings, would also be highly valuable [31–33]. Indeed, such models could facilitate accessible and individualized monitoring and prevention strategies. Deep learning methods offer a potential solution to achieve this as they can integrate heterogeneous inputs and model nonlinear fatigue dynamics beyond the capacity of traditional statistical techniques [34–36]. Once trained on high-fidelity laboratory datasets, such models can be deployed using wearable-derived signals, supporting practical, field-based monitoring of tissue loading [37]. However, current data-driven frameworks frequently rely on low-dimensional or large linear features, limiting their ability to represent the layered, multiscale behavior of musculoskeletal fatigue-failure processes and reducing sensitivity to subtle variations in tissue loading and structural deformity. A model that integrates deep learning with biomechanical modeling may however overcome these limitations. Specifically, a Swarm Search Optimization–Convolutional Neural Network–Bidirectional Long Short-Term Memory–Hybrid Attention Mechanism (SSO–CNN–BiLSTM–HAM) model may be combined with musculoskeletal modelling to enable real-time prediction of CL, CD, and PFF. This model is particularly well-suited for predicting tissue loading and damage during running because it combines complementary strengths in feature extraction, temporal learning, and optimization. Specifically, the CNN component effectively captures local biomechanical patterns in running data, while the BiLSTM layer models bidirectional temporal dependencies that are essential for understanding how loading changes over time. The hybrid attention mechanism further enhances performance by selectively weighing the most relevant biomechanical features and time steps, improving sensitivity to signals relevant for predicting loading and damage. In addition, swarm search optimization improves parameter tuning and model convergence, leading to more accurate and stable predictions.

Accordingly, this study aims to: (1) examine how lower-limb loading (CL, CD, PFF) at common injury locations—the Achilles tendon, plantar fascia, patellofemoral joint, and tibia—differs between individuals with and without HV; (2) assess how well these loads at common injury locations can be predicted from wearable sensor (IMU) data. From a practical perspective, a better understanding of how foot deformations may affect biomechanical loading at common injury sites can provide a mechanistic basis for further interventions. Further, a model that uses wearable data to estimate injury risk may support early identification of excessive tissue loading during running and provide quantitative biomechanical indicators for individualized training regulation, injury prevention, and rehabilitation monitoring in runners with foot deformities.

## 2. Materials and methods

**Fig 1:**
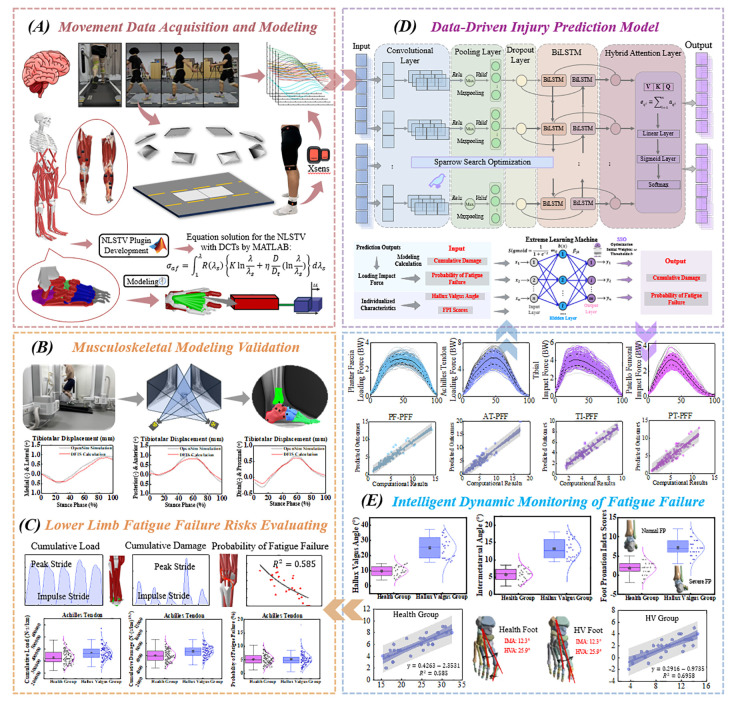
Overview of the overall workflow of the current study. **(A)** Movement Data Acquisition and Modeling. **(B)** Lower Limb Fatigue Failure Risks Evaluation. **(C)** Musculoskeletal Modeling Validation. **(D)** Data-Driven Injury Prediction Model. **(E)** Intelligent Dynamic Monitoring of Fatigue Failure.

The methodological framework of this study comprises six integrated components. Participants first performed standardized running trials on an instrumented treadmill to acquire experimental data (Fig 1A). Three-dimensional motion capture, and ground reaction forces were synchronously acquired, yielding multichannel kinematic–kinetic inputs that described stance-phase mechanics. A subject-specific foot–ankle musculoskeletal model was then constructed using MRI and CT data, incorporating 36 ligaments defined by dense connective tissue (DCT) properties **(**Fig 1A and Section A in S3 File). A nonlinear strain–time–viscoelastic constitutive framework characterized ligament behavior, allowing realistic simulation of tissue deformation and internal force transmission during running. Based on the simulated ligament and tendon loads, mechanical fatigue indices—including cumulative load (CL), cumulative damage (CD), and fatigue failure probability (PFF) for four common running injury locations: the Achilles tendon, plantar fascia, patellofemoral joint, and the tibia (Fig 1B). Model accuracy was validated by comparing predicted ligament elongations and joint displacements against dual fluoroscopic imaging system (DFIS) measurements, which showed strong spatial consistency with experimental results (Fig 1C). Finally, a data-driven injury prediction model integrating the SSO–CNN–BiLSTM–HAM architecture was established (Fig 1D). In the first stage, the model learned nonlinear spatiotemporal mappings between biomechanical inputs and ligament forces to output initial fatigue predictions. In the second stage, the Foot Posture Index (FPI) was introduced as a key structural factor to refine and optimize the predictive accuracy of fatigue indices, allowing individualized modeling of pronation-related variability. This adaptive integration enhances the model’s ability to monitor fatigue progression and predict overuse injury risk in runners with hallux valgus (Fig 1E).

### 2.1. Ethics statement

The study protocol was approved by the Ethics Committee of Ningbo University (Approval No. RAGH20231006), and all participants provided informed consent before participating in any study activities.

### 2.2. Participants

Sample size estimation was performed using G*Power 3.1.9.7 (Heinrich Heine University, Germany) based on an independent-sample t-test (effect size = 0.26, α = 0.05, power = 0.8, allocation ratio = 1), resulting in a required sample of 26 participants per group. The HV group included individuals with mild (Hallux Valgus Angle (HVA): 15°–20°; Intermetatarsal Angle (IMA): 9°–11°) or moderate (HVA: 20°–40°; IMA: 11°–18°) bilateral hallux valgus, confirmed by weight-bearing dorsoplantar radiographs [4]. The anatomical and demographic characteristics of all subjects are summarized in Table 1, and group differences in foot morphology are illustrated in Fig 1E. Participants in the Control group exhibited no neuromusculoskeletal disorders that could influence gait performance.

**Table 1:** Detailed results of anatomical characteristics and parameters for the Control and HV groups.

Characteristics	Control Group	HV Group	*P*	*Q*
Number of Participants	26	26	/	
Age (years)	26.6 ± 5.7	25.8 ± 4.7	0.4357	
Height (m)	1.71 ± 4.48	1.68 ± 5.35	0.7187	
Body mass (kg)	65.2 ± 11.8	67.3 ± 9.4	0.8966	
Weekly Distance (km)	18.5 ± 7.3	15.8 ± 6.8	0.1349	
Running Experience (years)	4.5 ± 4.1	4.4 ± 3.7	0.081	
Foot Parameters				
Foot length (cm)	25.5 ± 3.1	25.8 ± 3.7	0.319	
Foot width (cm)	10.1 ± 1.2	9.8 ± 1.5	0.516	
HVA (°)	9.9 ± 3.2	23.8 ± 5.2	0.001	0.001
IMA (°)	6.1 ± 2.4	13.4 ± 2.7	0.001	0.001
FPI scores	1.7 ± 1.6	5.9 ± 1.8	0.001	0.001

Note: “*” represents significance with p < 0.05. HVA: Hallux valgus angle; FPI: Foot posture index; IMA: Intermetatarsal angle

### 2.3. Experimental protocol and procedures

Running experiments were conducted in the Biomechanics Laboratory of Ningbo University. A subject-specific foot–ankle musculoskeletal model incorporating the plantar fascia and Achilles tendon was adapted from previous models to meet the present research objectives [17,18]. Detailed foot-ankle modeling descriptions are provided in Section 2.4. Motion data were collected using an eight-camera VICON system (Vicon Metrics Ltd., UK; 200 Hz) synchronized with two AMTI force plates (AMTI, Watertown, USA; 1000 Hz) to record kinematic and kinetic variables during the stance phase. Simultaneously, lower-limb muscle activities were acquired using a wireless EMG system (1000 Hz), with maximum voluntary contraction (MVC) data obtained for activation normalization [16,38]. In addition, five wearable inertial measurement units (IMUs; Xsens, Henderson, USA) were attached to the lower limbs to record multichannel acceleration signals. Sensors were placed on the foot and at the distal and proximal segments of the tibia and femur to capture segmental motion during running. The IMU system was synchronized with the laboratory biomechanical measurements to enable integrated analysis of wearable motion signals and laboratory-based biomechanical variables (Section B in S3 File) [39]. Prior to formal testing, participants completed a standardized 10-minute warm-up. Foot Posture Index (FPI) scores were determined following international guidelines based on talar palpation, heel alignment, medial arch height, and forefoot adduction (-2/-1/0/1/2) [40]. The FPI score classifies foot posture into five levels: excessive inversion (-12), inversion (-5), normal (0), pronation (5) and excessive pronation (12).

To estimate per-kilometer cumulative load (CL), participants first ran 1 km continuously at 12 km/h on a Zebris treadmill (Zebris Medical GmbH, Germany) before overground testing. Two AMTI force plates were embedded mid-runway (20 m) with infrared timing gates (4 m apart) to control speed (3.33 ± 10% m/s). Each participant completed ten successful trials, ensuring right-foot and left-foot steps on the first and second force plates, respectively.

### 2.4. Data initial processing and collection

The stance phase of running was defined as the interval between the initial foot contact with the force plate (vertical ground reaction force > 10 N) and the instant of toe-off. All motion capture data were processed using Vicon Nexus 2.1.0 (Vicon Metrics Ltd., UK). Preprocessing involved several key steps: (a) isolating valid gait cycles, (b) labeling reflective markers, (c) interpolating missing trajectories, (d) removing misidentified markers, and (e) verifying marker connectivity. After preprocessing, trials were exported as C3D files and imported into Visual 3D 6.7.3 (C-Motion Inc., Germantown, USA) for inverse kinematics and dynamics analyses. To minimize signal noise, a zero-phase fourth-order Butterworth filter was employed with cut-off frequencies of 10 Hz for kinematic and 20 Hz for kinetic signals [17,18]. The filtered data were then converted into “*.osim*” format and imported into OpenSim 4.4 (Stanford University, USA) for advanced musculoskeletal simulation. In OpenSim, joint contact forces for the patellofemoral and ankle regions were computed within the local coordinate frames of the femur and tibia by combining joint reaction forces with dynamically optimized muscle vectors. The tibial force was derived as the resultant of the ankle joint contact force and the gravitational component acting at the center of mass of the distal tibial segment [40].

Raw electromyographic (EMG) data were first band-pass filtered (10–400 Hz, fourth-order Butterworth), then rectified and low-pass filtered at a 6 Hz cut-off. Each EMG signal was normalized to the subject’s maximum voluntary contraction (MVC) value to allow inter-individual comparison. Muscle activation levels were subsequently estimated using a recursive second-order nonlinear model, capturing the dynamic behavior of neuromuscular excitation [13].

### 2.5. Subject-specific foot-ankle musculoskeletal model creation and ligament DCT property setting

A subject-specific foot–ankle musculoskeletal model containing 32 muscles and 36 ligaments was developed to quantify ligament loading mechanics during running. Medical imaging data (MRI/CT) were combined to reconstruct individualized skeletal geometries, ligament attachment sites, and soft tissue stiffness characteristics, allowing the model to accurately represent subject-specific mechanical responses. The workflow included body-segment extraction, coordinate system registration, model alignment, and marker-set calibration [41]. To capture the nonlinear viscoelastic behavior of dense connective tissues (DCTs), a nonlinear strain–time–viscoelastic (NLSTV) constitutive model was implemented for all ligaments [42,43]. Each ligament was modeled as a bundle spanning at least two anatomical segments, with origin, insertion, and path coordinates derived from MRI-based reconstructions. The ligament tension was expressed as a nonlinear function of the fiber stretch ratio$\;\lambda =\frac{L_{e}}{L_{r}}$ [44].


$$\begin{array}{c} \sigma_{af}=-p\lambda^{-\text{1}}+\sigma_{e}(\lambda )+\sigma_{v}\left(\lambda ,\dot{\lambda }\right) \end{array}$$ (1)

$$\begin{array}{c} \left\{ \begin{array}{c} \sigma_{e}(\lambda )=\int_{\text{1}}^{\lambda }R\left(\lambda_{s}\right)\overset{―}{\sigma_{e}}(\frac{\lambda }{\lambda_{s}})d\lambda_{s}\\ \overset{―}{\sigma_{e}}(\lambda )=K\text{ln}\lambda \end{array}\right. \end{array}$$ (2)

where *K* = 70 MPa represents the collagen elastic modulus, *η* = 20 MPa·s denotes the viscosity coefficient, and R(λ) is the probability density function describing the sequential straightening of collagen fibers [17,44]. The distribution function follows a two-parameter Weibull form: ·····


$$\begin{array}{c} R(\lambda )=\frac{\alpha }{\beta }{\left(\frac{\text{ln}\lambda -\gamma }{\beta }\right)}^{\alpha -\text{1}}\text{exp}\left[-{\left(\frac{\text{ln}\lambda -\gamma }{\beta }\right)}^{\alpha }\right] \end{array}$$ (3)

Where α=4.5 and β=0.3 are the shape and scale factors of the probability distribution function, respectively, for the sequential straightening of collagen fibers under stress loading in the 2-parameter Weibull model [14,45,44].

A customized graphical user interface (GUI) developed in MATLAB (App Designer) was linked with OpenSim to evaluate ligament elongation and DCT forces [46,47]. The model incorporated a nonlinear solver (NLSR.dll) and applied residual reduction algorithms (RRA) and computed muscle control (CMC) for dynamic stability. Passive strain limits were constrained to 2.5–3.0% at ligament insertion points to preserve physiological accuracy [41,48]. A detailed description of these steps is provided in Section C in S3 File.

### 2.6. Lower limb tissue mechanical fatigue and cumulative injury

**Fig 2:**
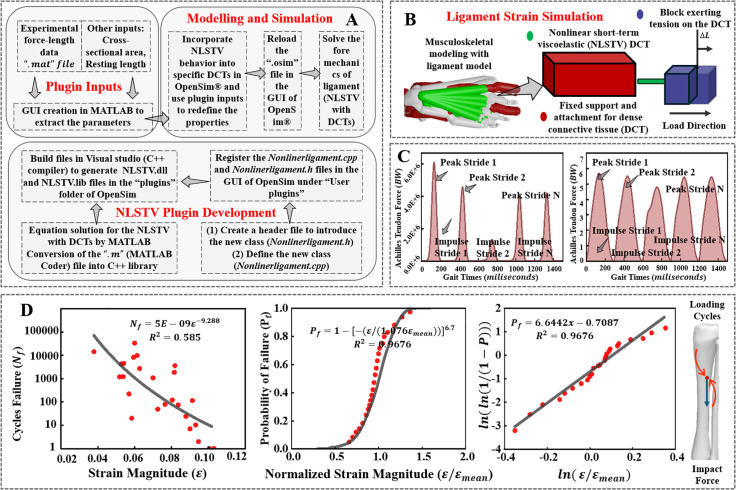
Integrated framework for structure-aware tissue load simulation and fatigue modeling in running. (A) Flow diagram of the NLSR plugin creation and DCT model simulation. (C) Representative Achilles tendon force profiles during the running stance phase, including stride-wise peak and impulse characteristics, as well as cumulative loading behavior. (D) Fatigue behavior modeling based on strain-dependent damage accumulation, including the relationship between strain magnitude and cycles to failure, probability of failure as a function of normalized strain, and the corresponding log-linear transformation for model fitting and parameter estimation.

As shown in **Fig 2**, this study quantified three fatigue-related biomechanical indices (CL, CD, and PFF) at four common injury sites—the Achilles tendon, plantar fascia, patellofemoral joint, and tibia—to evaluate tissue fatigue responses per kilometer of running [23,49]. The cumulative load was defined as the integral of tissue force over the stance phase multiplied by the number of steps per kilometer [50,51]:


$$\begin{array}{c} CL=n\int_{t_{i}}^{t_{f}}\;x_{s}dt \end{array}$$ (4)

where *n* is the step count and *xₛ* represents the peak load during stance. To account for the nonlinear relationship between load magnitude and fatigue life, a weighted impulse was introduced following the inverse power-law formulation [22,52].


$$\begin{array}{c} CD={\left[n\int_{t_{i}}^{t_{f}}{\left(x_{s}\right)}^{b}d_{t}\right]}^{\frac{\text{1}}{b}} \end{array}$$ (5)

where *b* denotes the tissue-specific weighting factor (9.3 for Achilles tendon and Plantar fascia, 7.0 for patellofemoral and tibial bones). This approach captures the nonlinear load–damage interaction and the influence of cyclic loading frequency on tissue deterioration [22,29].

Furthermore, to represent biological variability and fatigue life dispersion, a Weibull statistical model was applied to estimate the probability of fatigue failure:


$$\begin{array}{c} P_{f}=\text{1}-e^{-{\left(\frac{\varepsilon_{f}}{\varepsilon_{f},\text{0}}\right)}^{mW}} \end{array}$$ (6)

where $\varepsilon_{f\;}$is the tissue strain, ${(\varepsilon }_{f},\text{0})$ the reference strain at 63.2% failure probability, and mW the Weibull modulus (6.7 in this study). By combining CL, CD, and PFF analyses, the model characterizes how repetitive loading contributes to tissue fatigue and injury susceptibility in runners with hallux valgus. Full derivations and parameter calibration procedures are detailed in Section D in S3 File.

### 2.7. Model validation

**Fig 1C** illustrates the workflow and outcomes of the foot–ankle displacement analysis conducted with a high-speed dual fluoroscopic imaging system (DFIS). A personalized three-dimensional foot–ankle–knee model of the dominant limb was reconstructed from medical imaging datasets. To ensure numerical stability and spatial fidelity, mesh element sizes were defined as 2 mm for osseous and soft-tissue structures and 0.5 mm for cartilage and ligament components. The simulated X-ray sources were arranged to replicate the orthogonal configuration of the in-lab fluoroscopic setup, reproducing the relative orientation of the detectors and emitters. The displacement trajectories of the tibiotalar and talocalcaneal joints derived from DFIS recordings showed excellent concordance with the corresponding musculoskeletal simulation outputs across all anatomical planes, thereby validating the accuracy of the DCT-integrated foot–ankle model. Additional procedural details are available in Section E in S3 File.

### 2.8. Data-driven deep learning model estimates for lower extremity injury risk modeling

To evaluate ligament loading and fatigue-related injury risk across four common running injury sites, we developed a multimodal deep learning framework that integrates wearable-sensor data with subject-specific musculoskeletal simulation outputs. Time-normalized acceleration waveforms collected from foot, tibial, and femoral IMUs (${\text{1}\text{0}\text{1}}_{data\;points}$) served as the primary inputs, while the predicted outputs included tissue forces for the plantar fascia, Achilles tendon, tibia, and patellofemoral joint. Each waveform was resampled to 101 points to represent the complete gait cycle, forming multichannel time-series inputs for model training. All input channels were normalized prior to training to reduce scale-related bias.

The dataset consisted of 52 participants, including 26 individuals with hallux valgus and 26 healthy controls. All data were collected under controlled laboratory conditions during instrumented running experiments, where wearable IMU signals and laboratory-based biomechanical measurements were recorded simultaneously. Each participant completed ten running trials, resulting in a total of 520 biomechanical trials. For each trial, synchronized multichannel IMU signals were recorded from sensors placed on the foot, tibia, and femur. In parallel, laboratory biomechanical measurements were used to perform subject-specific musculoskeletal simulations to estimate tissue loading profiles. Based on these simulations, fatigue-related biomechanical indices (CL, CD, and PFF) were derived for four common running injury sites: the plantar fascia, Achilles tendon, tibia, and patellofemoral joint. These simulation-derived indices served as reference targets for supervised learning. The resulting dataset therefore represents a multimodal biomechanical dataset, combining wearable time-series sensor signals with biomechanical fatigue metrics derived from musculoskeletal simulations.

To prevent potential information leakage associated with repeated trials from the same individual, data partitioning was performed at the participant level rather than the trial level. All trials from a given participant were assigned exclusively to the same dataset split during model development. Consequently, no participant contributed data to more than one subset of the dataset. Model evaluation was conducted using participant-stratified cross-validation, ensuring balanced representation of the hallux valgus and control groups across folds. Because each participant contributed multiple trials, all trials from the same participant were assigned to the same fold. Hyperparameter optimization was performed using the training data and an internal validation subset, while the test data were reserved exclusively for final performance evaluation.

As illustrated in Fig 1D, the deep learning framework was developed through a sequential integration of multiple computational components for modeling biomechanical time-series data derived from wearable sensors and laboratory-based biomechanical analysis. The model architecture was progressively constructed through four configurations with increasing structural complexity. First, a BiLSTM model was implemented to capture temporal dependencies within multichannel biomechanical time-series signals. Next, a CNN–BiLSTM model was developed by introducing convolutional layers to extract local spatial features from the wearable-sensor signals before temporal modeling. Subsequently, a SSO–CNN–BiLSTM model was constructed by incorporating the Sparrow Search Optimization (SSO) algorithm to optimize key hyperparameters of the network during training. Finally, the SSO–CNN–BiLSTM–HAM model was implemented by integrating a Hybrid Attention Mechanism (HAM), enabling the network to adaptively weight temporally and spatially relevant features within the input signals.

In the final model, FPI scores and HVA were added as structural inputs, allowing the network to incorporate personalized workload anthropometrical data into the prediction of fatigue indices (**Fig 1D**). For clarity, the original SSO–CNN–BiLSTM–HAM architecture is referred to as Model 1, whereas the version integrating FPI and HVA is defined as Model 2. Compared with Model 1, Model 2 enables the network to explicitly account for FPI and HVA–driven loading patterns, thereby likely improving the precision of CL, CD, and PFF estimation across all tissues, particularly in runners with hallux valgus. Detailed configurations, training procedures, and validation metrics are provided in Section F in S3 File.

### 2.9. Statistical analysis

All statistical analyses were performed using MATLAB R2022a (MathWorks, Inc., USA) and SPSS 24.0 (IBM Corp., Chicago, IL, USA). In MATLAB, custom scripts were employed to interpolate waveform data into 101 evenly spaced points, corresponding to the normalized 100% stance phase. The Kolmogorov–Smirnov test was used to verify data normality. For time-series variables across the stance phase, statistical parametric mapping (SPM)-based independent-sample t-tests were conducted in MATLAB to examine temporal differences between the Control and HV groups [18]. The significance threshold for SPM analyses was determined using random field theory correction at α = 0.05. For discrete biomechanical parameters—including anatomical indices, foot morphology measures, peak joint angles, joint moments, joint contact forces, running-related joint range of motion (ROM), and fatigue-failure indicators (CL, CD, and PFF)—independent-sample t-tests were performed in SPSS to compare the Control and HV groups during running. To further investigate the associations between foot deformity and fatigue-failure related biomechanical indicators, Pearson correlation and linear regression analyses were conducted to examine the relationships between hallux valgus angle (HVA), Foot Posture Index (FPI), and the fatigue-failure indices CL, CD, and PFF [17]. The significance level was set at α = 0.05. When multiple comparisons were performed across related variables, the Benjamini–Hochberg false discovery rate (FDR) procedure was applied to correct for multiple comparisons. To this purpose, variables were grouped into three domains based on their biomechanical characteristics: anatomical indices (HVA, IMA, FPI; n = 3), joint kinematics and kinetics (n = 20), and fatigue-failure related indices (CL, CD, and PFF across four tissues; n = 12) for the correction. All comparisons within each domain were adjusted independently using the FDR procedure, with statistical significance defined as q < 0.05.

## 3. Results

### 3.1. Comparison of participant characteristics

Fig 1E presents the hallux valgus angle (HVA) results for the Control group (HVA: 9.9° ± 3.0°; intermetatarsal angle [IMA]: 5.7° ± 1.8°; FPI score: 1.8 ± 1.7) and the HV group (HVA: 23.8° ± 5.1°; IMA: 13.1° ± 2.8°; FPI score: 5.9 ± 1.8). A significant difference was observed in FPI scores between the two groups. Moreover, HVA exhibited a strong positive correlation with FPI in both the Control (r = 0.81, p < 0.001) and HV groups (r = 0.83, p < 0.001).

### 3.2. Musculoskeletal model accuracy validation

The comparison results of measured and modelled muscle activation in the lower limb muscles are shown in Fig B in S1 File. The results from the musculoskeletal model simulation and the EMG sensor collection were very similar for both the Control group (Fig B in S1 File) and the HV group (Fig B in S1 File). Furthermore, this work compares tibiotalar displacement results obtained based on musculoskeletal simulations and DFIS calculations. The tibiotalar displacement results in the three directions were consistent, which demonstrates the feasibility and accuracy of the foot-ankle model with DCTs.

### 3.3. Comparison of joint angle and moment during running

**Fig 3:**
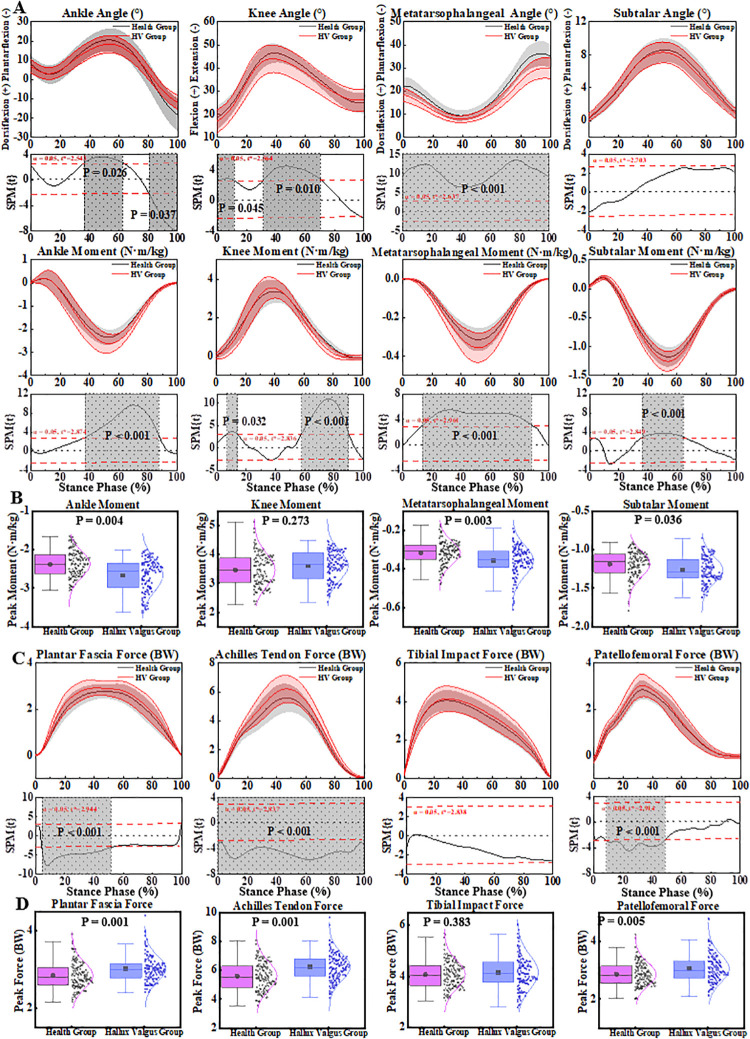
Detailed results of the joint angle, moment and impact loading force. (A) Results of statistical differences in sagittal plane joint angles and moments during the stance phase between the Control and HV groups. (B) Results of peak joint moment between the Control and HV groups. (C) Results of statistical differences in loading forces during the stance phase between the Control and HV groups. (D) Results of peak loading force between the Control and HV groups.

Results of the sagittal joint angle and moment comparisons are presented in Fig 3. Fig 3A displays the statistical results over the stance phase. For the ankle angle, the HV group exhibited significantly smaller than the Control group during 36.38-63.45% of the stance phase, and significantly greater plantar flexion than the control group during 81.60-100% of stance. The HV group also showed a greater ankle plantarflexion moment than the control group during 37.87-88.51% of stance.

For the knee angle, HV group showed significantly smaller than the control group during 1.41-11.60%, 31.72-71.39% of stance. For the Metatarsophalangeal angle, HV group was significantly smaller than the control group during the whole stance phase. For the Metatarsophalangeal moment, the HV group exhibited significantly greater than the Control group during 13.89-89.42% stance phase. For the subtalar angle and moment, there were no significant differences between the groups during the whole stance phase.

In the HV group, peak ankle (p = 0.004), and metatarsophalangeal (p = 0.003) moments were significantly greater, whereas ankle (p = 0.001), metatarsophalangeal (p = 0.001) range of motion (ROM) were significantly smaller compared to the Control group (Fig 1B, Table D in S2 File). There was no significant difference in peak knee (p = 0.273) moment, knee (p = 0.075) and subtalar (p = 0.22) ROM.

### 3.4. Loading at running-related injury regions

For the Plantar fascia force, the HV group was significantly greater than the Control group during 4.42-53.05% stance phase (**Fig 3C**), and HV group also showed greater peak Plantar fascia force (Fig 3D, Table D in S2 File). Similarly, the HV group showed significantly greater Achilles tendon force than the control group during the whole stance phase, and therefore, peak Achilles tendon force was also greater in the HV group (p = 0.001). For the Patellofemoral joint, the HV group showed significantly greater force than the control group during 8.95-48.8% of stance (p < 0.001), and HV also showed greater peak Patellofemoral force (p = 0.005). There was no significant difference in Tibial force (Fig 3).

### 3.5. Differences in the cumulative loadprobability of fatigu, damage and e failure between control and HV groups

**Fig 4:**
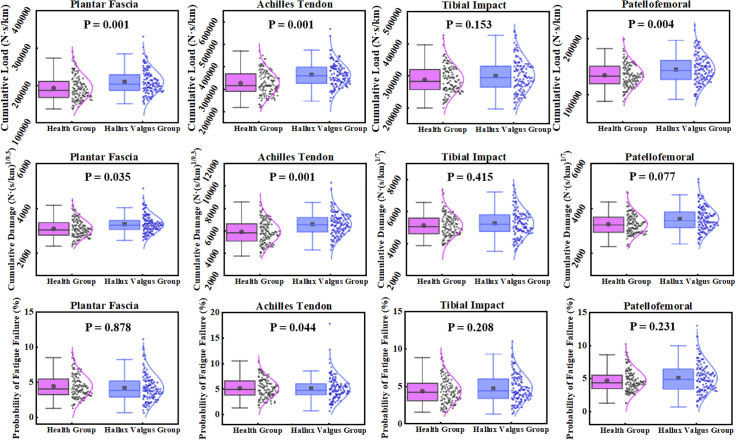
Detailed results of the cumulative load, cumulative damage and probability of fatigue failure in plantar fascia, Achilles tendon, tibial and patellofemoral joint in the Control and HV groups.

Detailed results of the CD and PFF are presented in Fig 4 and Table F in S2 File. For the Plantar fascia, the HV group showed higher CL (p = 0.001) and CD (p = 0.035) than the control group, but no difference in the PFF (p = 0.878). For the Achilles tendon, the HV group exhibited higher CL (p = 0.001), CD (p = 0.001), and PFF (p = 0.044) than the control group. For the patellofemoral joint, the HV group showed greater CL (p = 0.004) than the control group, but no difference in CD (p = 0.077) or PFF (p = 0.231). For the tibial loading, there was no significant difference in CL (p = 0.153), CD (p = 0.415), and PFF (p = 0.208) between the groups.

### 3.6. Relationship between the running injury risk and hallux valgus angle

**Fig 5:**
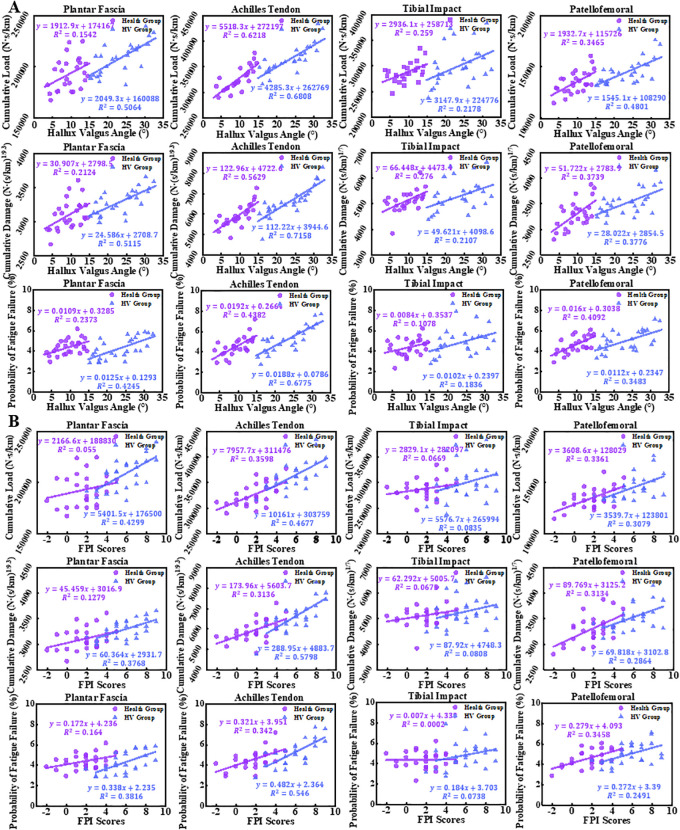
Visualization of the scatter distribution and linear relationship between HVA (A), FPI (B), cumulative load, cumulative damage, and probability of fatigue failure in Plantar fascia, Achilles tendon, Tibia, and Patellofemoral joint.

As shown in Fig 5A and Table E in S2 File, within the control group, the HVA was positively correlated with CL (r = 0.39), CD (r = 0.46), and PFF (r = 0.49) of the Plantar fascia; positively correlated with CL (r = 0.79), CD (r = 0.75), and PFF (r = 0.66) of the Achilles tendon; with CL (r = 0.51), CD (r = 0.53), and PFF (r = 0.33) of the tibia; and CL (r = 0.59), CD (r = 0.61), and PFF (r = 0.64) of the patellofemoral joint.

In the HV group, HVA was similarly positively correlated with CL (r = 0.71), CD (r = 0.72), and PFF (r = 0.65) of the Plantar fascia; CL (r = 0.83), CD (r = 0.85), and PFF (r = 0.82) of the Achilles tendon; CL (r = 0.47), CD (r = 0.46), and PFF (r = 0.43) of the tibia; and CL (r = 0.69), CD (r = 0.61), and PFF (r = 0.59) of the patellofemoral joint.

### 3.7. Relationship between the running injury risk and FPI scores

The correlations between FPI and CL, CD, and PFF are presented in Fig 5B and Table F in S2 File In the control group, FPI showed positive correlations with CL (r = 0.23), CD (r = 0.36), and PFF (r = 0.40) of the Plantar fascia; with CL (r = 0.60), CD (r = 0.56), and PFF (r = 0.58) of the Achilles tendon; with CL (r = 0.26), CD (r = 0.26), and PFF (r = 0.01) of the tibia; and with CL (r = 0.58), CD (r = 0.56), and PFF (r = 0.59) of the patellofemoral joint.

In the HV group, the correlations were generally stronger. FPI was positively correlated with CL (r = 0.66), CD (r = 0.61), and PFF (r = 0.62) of the Plantar fascia; with CL (r = 0.68), CD (r = 0.76), and PFF (r = 0.74) of the Achilles tendon; with CL (r = 0.29), CD (r = 0.28), and PFF (r = 0.27) of the tibia; and with CL (r = 0.55), CD (r = 0.54), and PFF (r = 0.50) of the patellofemoral joint.

### 3.8. Predicted results of CD and PFF based on SSO-CNN-BiLSTM-HAM model

Building upon this validated biomechanical framework, we further evaluated the effectiveness of the proposed deep learning architecture. Specifically, a series of baseline and progressively enhanced models were constructed, including BiLSTM, CNN–BiLSTM, SSO–CNN–BiLSTM, and SSO–CNN–BiLSTM–HAM. These models represent a hierarchical integration of temporal modeling, spatial–temporal feature extraction, parameter optimization, and attention-based feature refinement. Comparative results show a consistent reduction in prediction errors as model complexity increases, with the proposed SSO–CNN–BiLSTM–HAM achieving the lowest RMSE and NRMSE values. Detailed model configurations and extended comparative results are provided in Section F in S3 File (Figs E And F in S1 File). Based on these findings, the further results will focus on discussing the accuracy of this model in more detail.

**Fig 6:**
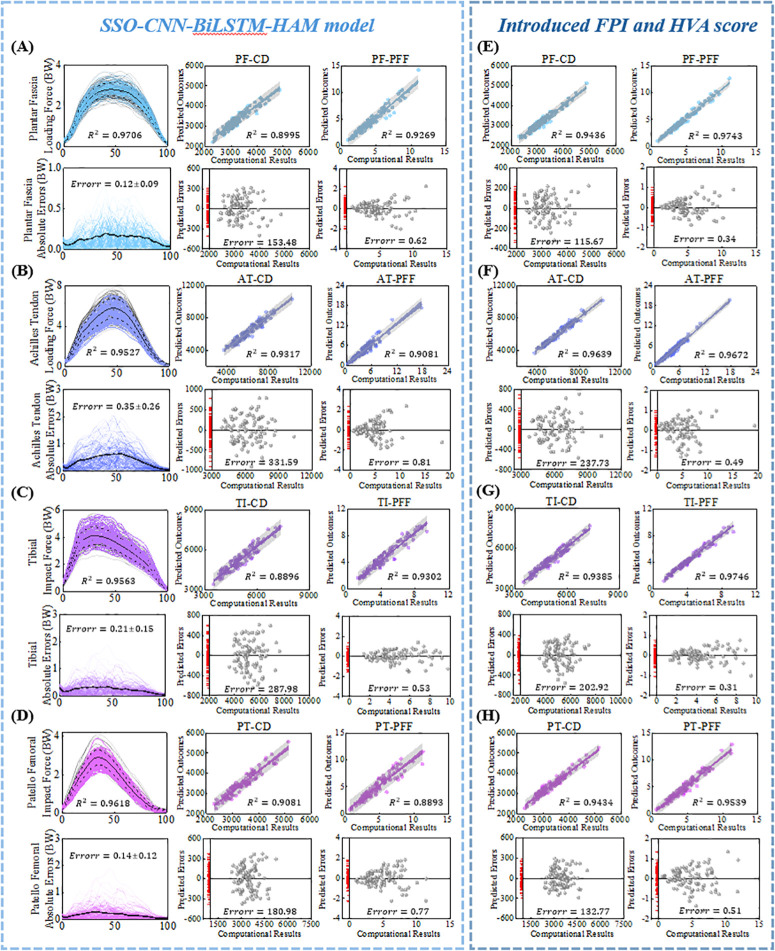
Visualization of the prediction errors of cumulative damage and probability of fatigue failure with different deep learning models relative to the cumulative damage and probability of fatigue failure obtained with the musculoskeletal model for the four common running injury locations. **(A–D)** Predicted and absolute errors of CD and PFF obtained from Model 1 for the plantar fascia, Achilles tendon, tibia, and patellofemoral joint, respectively. **(E–H)** Predicted and absolute errors of CD and PFF obtained from the Model 2 for the Plantar fascia, Achilles tendon, Tibia, and Patellofemoral joint, respectively.

Fig 6 illustrates the predictive performance of the proposed Model 1 across four key lower-limb structures, both before and after incorporating the FPI and HVA as adaptive input factors. The predicted loading forces demonstrated strong agreement with the reference data, with the plantar fascia (achieving an R² of 0.9706 and an absolute error of 0.12 ± 0.09 N·s/km, the Achilles tendon reaching an R² of 0.9527 with an error of 0.35 ± 0.36 N·s/km, the tibia showing an R² of 0.9563 and an error of 0.21 ± 0.15 N·s/km, and the patellofemoral joint achieving an R² of 0.9618 with an error of 0.14 ± 0.12 N·s/km. For the Plantar fascia, the cumulative damage (CD) prediction achieved an R² of 0.8995 with an absolute error of 153.48 N·(s/km)^1/7^, while the probability of fatigue failure (PFF) reached an R² of 0.9269 with an absolute error of 0.62% (Fig 6A). For the Achilles tendon, the model obtained R² values of 0.9317 (absolute error = 331.59 N·(s/km)^1/7^) for CD and 0.9081 (absolute error = 0.81%) for PFF (Fig 6B). In the Tibia joint, CD and PFF yielded R² values of 0.8896 (absolute error = 287.98 N·(s/km)^1/7^) and 0.9302 (absolute error = 0.53%), respectively  (Fig 6C). For the Patellofemoral joint, the CD prediction resulted in an R² of 0.9081 with an absolute error of 180.98 N·(s/km)^1/7^, and the PFF prediction achieved an R² of 0.8893 with an absolute error of 0.77% (Fig 6D).

After introducing the FPI and HVA as additional input factors, the Model 2 outputs showed improved fitting performance. In the Plantar fascia region, the R² values increased to 0.9436 (absolute error = 115.67 N·(s/km)^1/7^) for CD and 0.9743 (absolute error = 0.34%) for PFF (Fig 6E). The Achilles tendon region exhibited R² values of 0.9639 (absolute error = 237.73 N·(s/km)^1/7^) for CD and 0.9672 (absolute error = 0.49%) for PFF (Fig 6F). For the Tibia, the CD and PFF predictions achieved R² values of 0.9385 (absolute error = 202.92 N·(s/km)^1/7^) and 0.9746 (absolute error = 0.31%), respectively (Fig 6G). In the Patellofemoral joint, the corresponding R² values were 0.9434 (absolute error = 132.77 N·(s/km)^1/7^) for CD and 0.9539 (absolute error = 0.51%) for PFF (Fig 6H).

## 4. Discussion

This study aimed to explore how HV and foot pronation influence lower-limb biomechanical loading at four common running-related injury regions, including the Plantar fascia, Achilles tendon, Tibia, and patellofemoral joint. Specifically, we analyzed how structural characteristics of the feet in runners with HV are associated with pronation-related gait patterns, and how these variations may influence biomechanical characteristics during running. This work focuses on three key metrics to achieve this: CL, CD, and PFF. Changes in these indicators may reflect increased mechanical loading on tissues, particularly during prolonged running sessions. Furthermore, this study examines specific running-related injury regions, including the Plantar fascia, Achilles tendon, Tibia, and patellofemoral region, to deeply analyze the interaction of these biomechanical parameters and their influence on running performance. Preliminary findings partially support this hypothesis, showing a close link between the runners with HV and the occurrence of foot pronation.

This study found significant differences in joint angles during running between runners with and without HV, particularly in ROM of the ankle and metatarsophalangeal joints. Specifically, the HV group exhibited reduced ankle ROM during the mid-stance phase, along with a reduced metatarsophalangeal joint ROM (Fig 3). Additionally, HV patients generally exhibited reduced knee joint ROM. In line with the present findings, Kimura et al. reported increased subtalar dorsiflexion during gait tasks, suggesting compensatory rearfoot motion associated with structural deformity [53]. The alterations in ROM in the ankle and metatarsophalangeal joints observed in the present study may have consequences for performance and injury risk. Previous studies have reported associations between ankle ROM and running biomechanics, suggesting that altered ankle motion may influence gait stability and lower-limb loading patterns during running [4,5]. Changes in the ankle (p = 0.001) and metatarsophalangeal (p = 0.001) joint ROM may have led to the increased torque generation at both joints during running in HV individuals. The increased torques place greater stress on structures around the ankle joint and plantar tissues [54]. To explore this possibility, we employed musculoskeletal modelling to estimate tissue loading at four common running-related injury sites, enabling a more detailed assessment of internal load distribution beyond externally measured biomechanics.

The results of the musculoskeletal modelling demonstrate that the HV group had significantly higher plantar fascia force during the early to mid-stance phase (p < 0.001), increased Achilles tendon force throughout the complete stance phase (p = 0.001), and elevated Patellofemoral force from 8.95% to 48.8% of the stance phase (p < 0.001) compared to the control group (**Fig 4**). Simultaneously, to better understand how the biomechanical differences for a single step translate to a larger running distance, we therefore analyzed the cumulative load (i.e., load accumulated for one kilometer of running). In line with the per-step loading, the HV group experienced significantly higher CL of the Plantar fascia (p = 0.001), Achilles tendon (p = 0.001), and Patellofemoral joint (p = 0.004), but not the tibia. This progressive accumulation of load may be associated with an increased likelihood of fatigue-related tissue responses [55].

To assess the injury implications associated with these biomechanical changes, we also analyzed CD, which accounts for the non-linear relationship between loading and the damage incurred. This analysis showed significantly higher CD of the Achilles tendon and Patellofemoral force in the HV group. However, the plantar fascia did not show significant differences. This lack of significance may indicate that PFF is influenced not only by cumulative loading magnitude but also by nonlinear fatigue characteristics and inter-individual variability [56]. Mechanical fatigue reflects the progressive accumulation of tissue damage and fatigue-related mechanical burden during repetitive loading activities [24,51,52]. In this study, Model 1 demonstrated high predictive accuracy for the fatigue indices (R² = 0.89-0.9), with PFF showing superior fidelity compared to CL and CD. Moreover, adding FPI and HVA as an easy to obtain anthropometrical inputs significantly improved model performance. Compared to the Model 1, the Model 2 architecture exhibited consistently higher R² values and reduced absolute errors (R² = 0.94–0.97), demonstrating that personalized foot anthropometrical data adds further relevance to predict biomechanical loading. This improvement suggests that fatigue-related patterns in HV runners are not solely captured by mechanical loading variables, but are also associated with structural characteristics such as foot posture. These findings indicate that incorporating structural parameters may enhance the representation of individual variability in fatigue-related responses [23,57].

Another interesting finding of this study is the correlation between HV and foot pronation. Runners with HV typically exhibit more pronounced foot pronation. This may be because altered first metatarsophalangeal alignment can reduce medial forefoot stability and promote compensatory rearfoot eversion during stance. These alterations may influence gait mechanics and potentially contribute to increased lower-limb injury susceptibility. Previous studies have reported that individuals with HV often exhibit altered muscle activation and force balance around the first metatarsophalangeal joint. These muscles play an important role in maintaining dynamic arch stability during gait [58–60]. After incorporating FPI and HVA into the predictive framework, Model 2 revealed clearer associations between pronation magnitude and fatigue indices. In runners with hallux valgus, higher pronation levels were consistently accompanied by elevated fatigue metrics across all modeled tissues. For example, before introducing FPI and HVA, CD and PFF values of the Achilles tendon and patellofemoral joint showed moderate predictive performance (R² = 0.93 and 0.91), whereas FPI and HVA integration increased the predictive agreement to R² = 0.96 and 0.94, respectively, indicating that foot posture provides essential structural information for interpreting tissue loading pathways. Moreover, CD and PFF values of the Achilles tendon and patellofemoral region remained elevated in the HV group, reflecting the cumulative mechanical burden imposed by altered gait patterns [61,62]. The enhanced model performance after FPI and HVA integration not only validates the mechanical influence of pronation but also demonstrates the necessity of embedding structural deformity information into data-driven fatigue models. Through this dual-model comparison, our findings suggest that intelligent prediction frameworks benefit substantially from combining musculoskeletal simulation outputs with structure-aware deep learning features, providing a more mechanistic and individualized interpretation of fatigue progression [29].

In summary, this study integrates biomechanical modeling with deep learning–based fatigue-failure prediction to characterize associations between HV-related structural deformities and tissue-specific loading and fatigue patterns during running. By combining CL, CD, and PFF with FPI- and HVA-driven adaptive optimization, the framework provides a multiscale perspective linking foot deformity, mechanical stress, and fatigue-failure related progression. Nevertheless, several limitations should be acknowledged. First, the sample size was relatively small, which may have limited the generalizability of the results to populations with varying degrees of HV severity. Second, the musculoskeletal simulations relied on idealized boundary conditions and assumed consistent ligament properties, which may not fully capture individual variability in tissue material behavior. In addition, although marker placement was adapted to account for hallux valgus morphology, the OpenSim model was based on a scaled generic template rather than a fully subject-specific reconstruction. Third, although wearable IMU signals were used as the primary input features of the predictive framework, all wearable data in this study were collected under controlled laboratory conditions together with laboratory-grade biomechanical measurements, and at only one running speed. Therefore, the present framework should be interpreted as a laboratory-validated proof-of-concept rather than a fully field-deployed wearable monitoring system. In real-world running environments, additional variability may arise from factors such as sensor placement differences, environmental noise, and variations in running context (including speed, gradients, footwear, etc.), which may influence prediction stability. Future work will therefore focus on validating the framework using field-based wearable recordings and longitudinal monitoring to further enhance the ecological validity and practical applicability of the proposed fatigue prediction approach.

## 5. Conclusion

This study demonstrates that runners with HV exhibited larger loading and probability of fatigue-failure at the plantar fascia, Achilles tendon, and patellofemoral joint, with no differences in the tibia. Incorporating the FPI and HVA as a structural modifier further enhanced the model’s ability to capture individual variability, emphasizing the influence of foot morphology on fatigue progression. The refined framework effectively characterized the nonlinear degradation of soft tissues, showing that increased HV severity and pronation magnitude are accompanied by higher cumulative load and damage levels. By integrating biomechanical modeling with FPI and HVA-informed deep learning, this study establishes a structure-aware, individualized approach for predicting and preventing fatigue-related running injuries.

## Supporting information

S1 File Supplemenatry figures A-G. (DOCX)

S2 File Supplemetary tables A-G. (DOCX)

S3 File Supplementary Section A-F. (DOCX)
