# Correction: Structure-aware fatigue modeling in foot deformities: A digital health framework for tissue-specific running injury risk prediction using multi-modal data

**DOI:** 10.1371/journal.pdig.0001658

**Published:** 2026-08-11

**Authors:** Zhifeng Zhou, Huiyu Zhou, Datao Xu, Bas Van Hooren, Yi Yuan, Tianle Jie, Xiangli Gao, Xiuye Qu, Zanni Zhang, Zixiang Gao, Liangliang Xiang, Yaodong Gu

There are errors in the author affiliations. The correct affiliations are as follows:

Zhifeng Zhou^1^, Huiyu Zhou^1^, Datao Xu^1^, Bas Van Hooren^2^, Yi Yuan^3^, Tianle Jie^1,4^, Xiangli Gao^1,5^, Xiuye Qu^1^, Zanni Zhang^1^, Zixiang Gao^6^, Liangliang Xiang^7^, Yaodong Gu^1^

**1** Faculty of Sports Science, Ningbo University, Ningbo, China, **2** Department of Nutrition and Movement Sciences, NUTRIM Institute of Nutrition and Translational Research in Metabolism, Maastricht University Medical Centre + , Universiteitssingel 50, Maastricht, The Netherlands, **3** Research Academy of Medicine Combining Sports, Ningbo No. 2 Hospital, Ningbo, China, **4** The Doctoral School on Safety andSecurity Sciences, Óbuda University, Budapest, Hungary, **5** Research Center for Molecular ExerciseScience, Hungarian University of Sports Science, Budapest, Hungary, **6** Human Performance Laboratory, Faculty of Kinesiology, University of Calgary, Calgary, Alberta, Canada, **7** KTH Move Ability Lab,Department of Engineering Mechanics, KTH Royal Institute of Technology, Stockholm, Sweden.
