# Supplementary material for: Structure-aware fatigue modeling in foot deformities: A digital health framework for tissue-specific running injury risk prediction using multi-modal data
Source: PLOS Digit Health. 2026 Jul 10;5(7):e0001537. doi: 10.1371/journal.pdig.0001537 (PMC13354006; doi:10.1371/journal.pdig.0001537)
Supplement: S1 File — (DOCX) [file pdig.0001537.s001.docx]

**Fig A**

**Fig A.** Illustration of the musculoskeletal model creation for the subject-specific foot-ankle.

**Fig B**


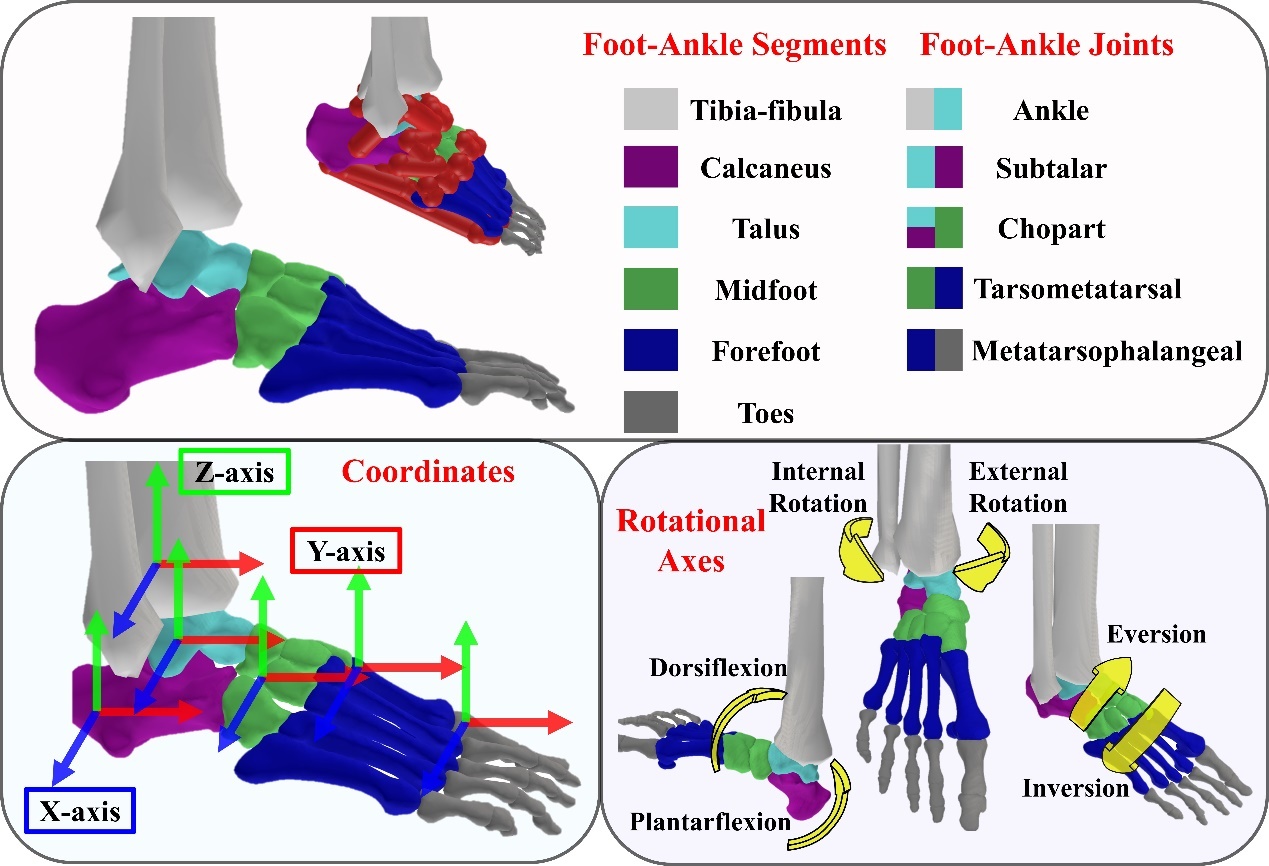


**Fig B.** Illustration of the foot-ankle model structure.

**Fig C**


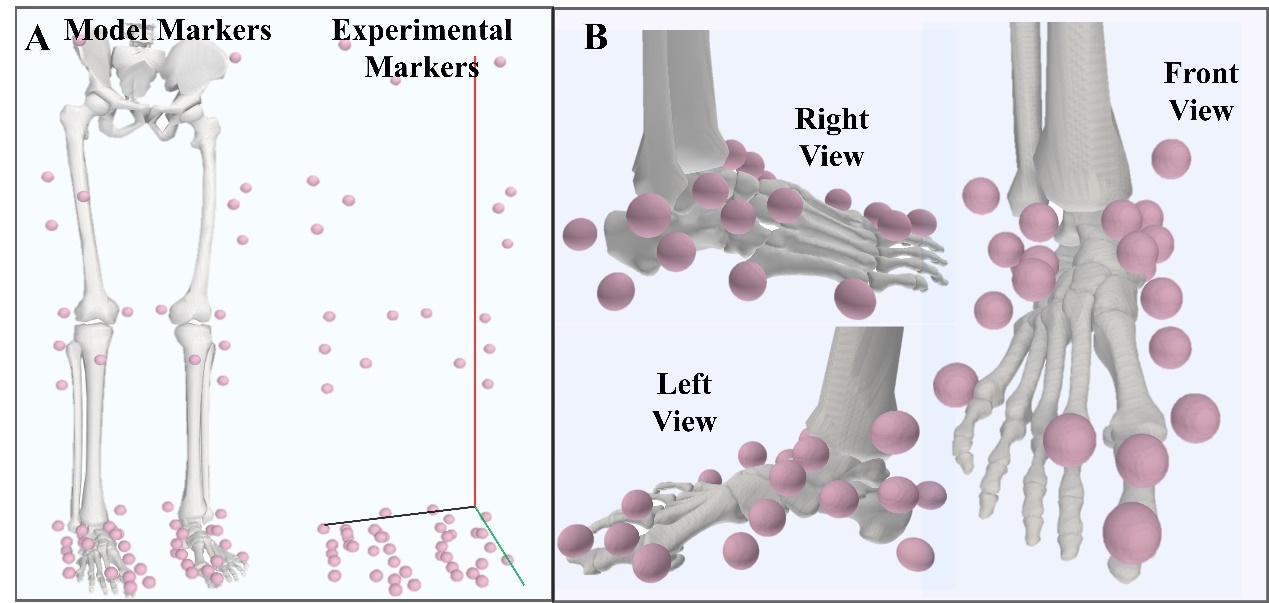


**Fig C.** Illustration of reflective marker point setup. (A) Illustration of the marker setup for the whole lower extremity. (B) Illustration of the marker setup for the foot-ankle model.

**Fig D**

**Fig D.** Detailed results of the IMD and corresponding weight attribution of marker points on the foot of subject 1 in one experiment. Marker points 1 to 20 are corresponding to MM, LM, PT, CT, LPC, MPC, TT, HT, TMT, TC, TN, CN1, CN3, SMB1, HMB1, SMB5, HMB5, FPP, FDP, and SPP, respectively.

**Fig E**

**Fig E.** Illustration of the muscle activation in lower limb muscles. (A) Muscle activation results in the healthy group. (B) Muscle activation results in the hallux valgus group.

**Fig F**

**Fig F.** Model prediction errors results. (A) Prediction errors of CD based on BiLSTM model. (B) Prediction errors of CD based on CNN-BiLSTM model. (C) Prediction errors of CD based on SSO–CNN-BiLSTM model. (D) Prediction errors of CD based on SSO–CNN-BiLSTM-HAM model. (E) Results of RMSE. (F) Results of NRMSE.

**Fig G**

**Fig G.** Model prediction errors results. (A) Prediction errors of PFF based on BiLSTM model. (B) Prediction errors of PFF based on CNN-BiLSTM model. (C) Prediction errors of PFF based on SSO–CNN-BiLSTM model. (D) Prediction errors of PFF based on SSO–CNN-BiLSTM-HAM model. (E) Results of RMSE. (F) Results of NRMSE.
