# Supplementary material for: Structure-aware fatigue modeling in foot deformities: A digital health framework for tissue-specific running injury risk prediction using multi-modal data
Source: PLOS Digit Health. 2026 Jul 10;5(7):e0001537. doi: 10.1371/journal.pdig.0001537 (PMC13354006; doi:10.1371/journal.pdig.0001537)
Supplement: S2 File — (DOCX) [file pdig.0001537.s002.docx]

**Table A**. Detailed results of anatomical characteristics and parameters for the healthy and HV groups.

| Characteristics | Health Group | HV Group | P |
| --- | --- | --- | --- |
| Demographics |  |  |  |
| Number of Participants | 26 | 26 | / |
| Age (years) | 25.8±4.5 | 26.7±4.8 | / |
| Height (m) | 1.76±0.53 | 1.75±0.47 | / |
| Weight (kg) | 70.1±5.7 | 68.8±6.8 | / |
| Investigated Foot (right/left) | 13/13 | 14/12 | / |
| BMI | 22.7±2.6 | 23.1±3.2 | 0.274 |
| Weekly Mileage (km) | 20.5±5.8 | 18.7±6.4 | 0.096 |
| Running Experience (years) | 4.7±3.1 | 4.3±2.8 | 0.105 |
| Foot Parameters |  |  |  |
| Foot length (cm) | 26.3±2.2 | 26.1±1.9 | 0.257 |
| Foot width (cm) | 11.3±0.5 | 11.4±0.4 | 0.338 |
| HVA (°) | 9.9±3.0 | 23.8±5.1 | 0.001 |
| IMA (°) | 5.7±1.8 | 13.1±2.8 | 0.001 |
| FPI scores | 1.8±1.7 | 5.9±1.8 | 0.001 |
| Gait Temporal Parameters |  |  |  |
| Stride Length (cm) | 199.6±7.2 | 200.4±6.9 | 0.867 |
| Stride Time (s) | 0.60±0.04 | 0.61±0.03 | 0.695 |
| Cadence (steps/min) | 182.8±6.6 | 181.1±5.7 | 0.731 |

Note: “*” represents significance with p < 0.05. HVA: Hallux valgus angle; FPI: Foot pronation index; IMA: Intermetatarsal angle

**Table B.** Foot muscle property settings.

|  | | Parameters of the Foot Intrinsic Muscles. | | | Geometry Path of the Intrinsic Muscles | | |
| --- | --- | --- | --- | --- | --- | --- | --- |
| Big Toe Group | | Max Isometric Force | Physiologic Cross-Sectional Area (PCSA) | Origin | Via Points | Insertion | Model Segment |
| ABDH | Abductor Hallucis | 301.76 | 6.68 | Calcaneus | Navicular, Metatarsal | Proximal Phalange1 | Calcaneus, Midfoot, Forefoot, Toes |
| FHBM | Flexor Hallucis Brevis Medialis | 81.31 | 1.8 | Navicular | Metatarsal 1, | Proximal Phalange 1 | Midfoot, Forefoot, Toes |
| FHBL | Flexor Hallucis Brevis Lateralis | 95.77 | 2.12 | Navicular | Metatarsal 1, | Proximal Phalange 1 | Midfoot, Forefoot, Toes |
| ADHT 1 | Adductor Hallucis Transverse 1 | 9.34 | 0.21 | Metatarsal 3 | Metatarsal 1 | Proximal Phalange 1 | Forefoot, Toes |
| ADHT 2 | Adductor Hallucis Transverse 2 | 9.34 | 0.21 | Metatarsal 4 | Metatarsal 1 | Proximal Phalange 1 | Forefoot, Toes |
| ADHO 1 | Adductor Hallucis Oblique 1 | 74.39 | 1.65 | Metatarsal 2 | Metatarsal 1 | Proximal Phalange 1 | Forefoot, Toes |
| ADHO 2 | Adductor Hallucis Oblique 2 | 74.39 | 1.65 | Metatarsal 3 | Metatarsal 1 | Proximal Phalange 1 | Forefoot, Toes |
| ADHO 3 | Adductor Hallucis Oblique 3 | 74.39 | 1.65 | Metatarsal 4 | Metatarsal 1 | Proximal Phalange 1 | Forefoot, Toes |
| EHB | Extensor Hallucis Brevis | 60.53 | 1.34 | Calcaneus | Intermediate Cuneiform, Metatarsal 1, | Proximal Phalange | Calcaneus, Midfoot, Forefoot, Toes |
| Dorsal Interosseous | | Max Isometric Force | Physiologic Cross-Sectional Area (PCSA) | Origin | Via Points | Insertion | Model Segment |
| DI 1 | Dorsal Interosseous 1 | 79.02 | 1.7 | Calcaneus | / | / | Calcaneus, Forefoot |
| DI 2 | Dorsal Interosseous 2 | 76.87 | 1.68 | Calcaneus | / | / | Calcaneus, Forefoot |
| DI 3 | Dorsal Interosseous 3 | 73.11 | 1.64 | Calcaneus | / | / | Calcaneus, Forefoot |
| DI 4 | Dorsal Interosseous 4 | 123.52 | 2.72 | Calcaneus | / | / | Calcaneus, Forefoot |
| EDB 2 | Extensor Digitorum Brevis 2 | 35.69 | 0.79 | Calcaneus | Metatarsal 3, Metatarsal 2 | / | Calcaneus, Forefoot |
| EDB 3 | Extensor Digitorum Brevis 3 | 23.04 | 0.51 | Calcaneus | Metatarsal 3, Metatarsal 3 | / | Calcaneus, Forefoot |
| EDB 4 | Extensor Digitorum Brevis 4 | 19.88 | 0.44 | Calcaneus | Metatarsal 4, Metatarsal 4 | / | Calcaneus, Forefoot |
| Plantar interosseous | | Max Isometric Force | Physiologic Cross-Sectional Area (PCSA) | Origin | Via Points | Insertion | Model Segment |
| PI 1 | Plantar interosseous 1 | 51.38 | 1.23 | Calcaneus | / | / | Calcaneus, Midfoot, Forefoot, Toes |
| PI 2 | Plantar Interosseous 2 | 61.24 | 1.41 | Calcaneus | / | / | Calcaneus, Midfoot, Forefoot, Toes |
| PI 3 | Plantar Interosseous 3 | 58.92 | 1.38 | Calcaneus | / | / | Calcaneus, Midfoot, Forefoot, Toes |
| FDB 2 | Flexor Digitorum Brevis 2 | 80.41 | 1.78 | Calcaneus | Cuboid, Metatarsal 2, Proximal Phalange 2 | Proximal Phalange 2 | Calcaneus, Midfoot, Forefoot, Toes |
| FDB 3 | Flexor Digitorum Brevis 3 | 67.31 | 1.49 | Calcaneus | Cuboid, Metatarsal 3, Proximal Phalange 3 | Proximal Phalange 3 | Calcaneus, Midfoot, Forefoot, Toes |
| FDB 4 | Flexor Digitorum Brevis 4 | 56.92 | 1.26 | Calcaneus | Cuboid, Metatarsal 4, Proximal Phalange 4 | Proximal Phalange 4 | Calcaneus, Midfoot, Forefoot, Toes |
| FDB 5 | Flexor Digitorum Brevis 5 | 15.81 | 0.35 | Calcaneus | Cuboid, Metatarsal 5, Proximal Phalange 5 | Proximal Phalange 5 | Calcaneus, Midfoot, Forefoot, Toes |
| QPM | Quadratus Plantar Medialis 1, 2, 3 | 29.51 | 0.65 | Calcaneus | / | Flexor Digitorum Longus Tendon | Midfoot, Forefoot |
| QPL | Quadratus Plantar Lateralis 1, 2, 3 | 15.06 | 0.33 | Calcaneus | / | Flexor Digitorum Longus Tendon | Midfoot, Forefoot |
| Lumbricals | | Max Isometric Force | Physiologic Cross-Sectional Area (PCSA) | Origin | Via Points | Insertion | Model Segment |
| LB 2 | Lumbricals 2 | 18.07 | 0.41 | Flexor Digitorum Longus Tendon | Metatarsal 2 | Proximal Phalange 2 | Midfoot, Forefoot, Toes |
| LB 3 | Lumbricals 3 | 18.07 | 0.41 | Flexor Digitorum Longus Tendon | Metatarsal 3 | Proximal Phalange 3 | Midfoot, Forefoot, Toes |
| LB 4 | Lumbricals 4 | 13.55 | 0.34 | Flexor Digitorum Longus Tendon | Metatarsal 4 | Proximal Phalange 4 | Midfoot, Forefoot, Toes |
| LB 5 | Lumbricals 5 | 8.13 | 0.18 | Flexor Digitorum Longus Tendon | Metatarsal 5 | Proximal Phalange 5 | Midfoot, Forefoot, Toes |
| Little toe group | | Max Isometric Force | Physiologic Cross-Sectional Area (PCSA) | Origin | Via Points | Insertion | Model Segment |
| ABDM | Abductor Digiti Minimi 1,2, 3 | 57.07 | 1.26 | Calcaneus | Metatarsal 5, Proximal Phalange 5, | Proximal Phalange 5 | Calcaneus, Midfoot, Forefoot, Toes |
| FDMB | Flexor Digiti Minimi Brevis | 90.35 | 2 | Metatarsal 5 | Metatarsal 5 | Proximal Phalange 5 | Forefoot, Toes |
| FDB 5 | Flexor Digitorum Brevis 5 | 15.81 | 0.35 | Calcaneus | Cuboid, Metatarsal 5, Proximal Phalange 5 | Proximal Phalange 5 | Calcaneus, Midfoot, Forefoot, Toes |
| Others | | Max Isometric Force | Physiologic Cross-Sectional Area (PCSA) | Origin | Via Points | Insertion | Model Segment |
| FHL | Flexor Hallucis Longus | 322.02 | 6.84 | Fibula | Tibia | Proximal Phalange 1 | Fibula, Tibia, Midfoot, Forefoot, Toes |
| EHL | Extensor Hallucis Longus | 162.57 | 3.53 | Fibula | Tibia, Medial cuneiform, Distal phalanx 1 | Proximal Phalange 5 | Fibula, Tibia, Midfoot, Forefoot, Toes |
| EHB | Extensor Hallucis Brevis | 60.53 | 1.34 | Fibula | Tibia, Metatarsal | Proximal Phalange 1 | Fibula, Tibia, Midfoot, Forefoot, Toes |
| EDL | Extensor Digitorum Longus | 512.92 | 10.06 | Tibia | Metatarsal | middle phalanx | Tibia, Midfoot, Forefoot, Toes |
| FFDL | Flexor Digitorum Longus | 310.46 | 6.71 | Tibia | Metatarsal | distal phalanges | Tibia, Midfoot, Forefoot, Toes |

**Table C.** Foot ligament property settings.

| Parameters of the Foot Ligaments | | | | Ligament Geometry Path | | |
| --- | --- | --- | --- | --- | --- | --- |
|  | Ligament | Origin | Via Points | Insertion | Resting Length (cm) | Model Segment |
| PTIFL | Posterior Tibiotalar | Tibia | / | Talus | 1.982 | Tibia, Talus |
| ATIFL | Anterior Tibiotalar | | / | Talus | 2.534 | Tibia, Talus |
| TCAL | Tibiocalcaneal | | / | Calcaneus | 3.015 | Tibia, Calcaneus |
| TNAL | Tibionavicular | | / | Navicular | 4.08 | Tibia, Midfoot |
| PTAFL | Posterior Talofibular | | / | Talus | 2.347 | Tibia, Talus |
| ATAFL | Anterior Talofibular | | / | Talus | 2.041 | Tibia, Talus |
| CFL | Calcaneofibular | | / | Calcaneus | 3.3 | Tibia, Calcaneus |
| PF 1 | Plantar Fascia 1 | Calcaneus | First metatarsal | First Proximal Phalange | 14.29 | Calcaneus, Forefoot, Toes |
| PF 2 | Plantar Fascia 2 | Calcaneus | Second metatarsal | Second Proximal Phalange | 14.404 | Calcaneus, Forefoot, Toes |
| PF 3 | Plantar Fascia 3 | Calcaneus | Third metatarsal | Third Proximal Phalange | 14.335 | Calcaneus, Forefoot, Toes |
| PF 4 | Plantar Fascia 4 | Calcaneus | Fourth metatarsal | Fourth Proximal Phalange | 13.612 | Calcaneus, Forefoot, Toes |
| PF 5 | Plantar Fascia 5 | Calcaneus | Fifth Metatarsal | Fifth Proximal Phalange | 12.815 | Calcaneus, Forefoot, Toes |
| LP 1 | Longitudinal Plantar 1 | Calcaneus | Cuboid | Second Metatarsal | 8.037 | Calcaneus, Midfoot, Forefoot |
| LP 2 | Longitudinal Plantar 2 | Calcaneus | Cuboid | Third Metatarsal | 7.665 | Calcaneus, Midfoot, Forefoot |
| LP 3 | Longitudinal Plantar 3 | Calcaneus | Cuboid | Fourth Metatarsal | 7.153 | Calcaneus, Midfoot, Forefoot |
| LP 4 | Longitudinal Plantar 4 | Calcaneus | Cuboid | Fifth Metatarsal | 6.389 | Calcaneus, Midfoot, Forefoot |
| CNP 1 | Calcaneonavicular Plantar 1 | Calcaneus | / | Navicular | 1.968 | Calcaneus, Midfoot |
| CNP 2 | Calcaneonavicular Plantar 2 | Calcaneus | / | Navicular | 1.214 | Calcaneus, Midfoot |
| CNP 3 | Calcaneonavicular Plantar 3 | Calcaneus | / | Navicular | 1.041 | Calcaneus, Midfoot |
| CCP 1 | Calcaneocuboid Plantar 1 | Calcaneus | / | Cuboid | 1.767 | Calcaneus, Midfoot |
| CCP 2 | Calcaneocuboid Plantar 2 | Calcaneus | / | Cuboid | 2.296 | Calcaneus, Midfoot |
| CCD | Calcaneocuboid Dorsal | Calcaneus | / | Cuboid | 2.543 | Calcaneus, Midfoot |
| TND 1 | Talonavicular Dorsal 1 | Talus | / | Navicular | 1.164 | Talus, Midfoot |
| TND 2 | Talonavicular Dorsal 2 | Talus | / | Navicular | 1.164 | Talus, Midfoot |
| CNB | Calcaneonavicular Bifurcate | Calcaneus | / | Navicular | 1.659 | Calcaneus, Midfoot |
| CCB | Calcaneocuboid Bifurcate | Calcaneus | / | Cuboid | 1.422 | Calcaneus, Midfoot |
| TTD 1 | Tarsometatarsal Dorsal 1 | Medial cuneiform | / | First Metatarsal | 1.271 | Midfoot, Forefoot |
| TTD 2 | Tarsometatarsal Dorsal 2 | Intermediate cuneiform | / | Second Metatarsal | 0.963 | Midfoot, Forefoot |
| TTD 3 | Tarsometatarsal Dorsal 3 | Lateral cuneiform | / | Third Metatarsal | 0.684 | Midfoot, Forefoot |
| TTD 4 | Tarsometatarsal Dorsal 4 | Cuboid | / | Fourth Metatarsal | 0.852 | Midfoot, Forefoot |
| TTD 5 | Tarsometatarsal Dorsal 5 | Cuboid | / | Fifth Metatarsal | 0.733 | Midfoot, Forefoot |
| TTP 1 | Tarsometatarsal Plantar 1 | Medial cuneiform | / | First Metatarsal | 0.903 | Midfoot, Forefoot |
| TTP 2 | Tarsometatarsal Plantar 2 | Intermediate Cuneiform | / | Second Metatarsal | 0.745 | Midfoot, Forefoot |
| TTP 3 | Tarsometatarsal Plantar 3 | Lateral cuneiform | / | Third Metatarsal | 0.697 | Midfoot, Forefoot |
| TTP 4 | Tarsometatarsal Plantar 4 | Cuboid | / | Fourth Metatarsal | 0.767 | Midfoot, Forefoot |
| TTP 5 | Tarsometatarsal Plantar 5 | Cuboid | / | Fifth Metatarsal | 0.897 | Midfoot, Forefoot |

**Table D**. Detailed results of the peak joint angle, moment, force and range of motion between the healthy and HV groups.

| Variables | | Health Group | HV Group | Difference 95% Confidence Interval | Mean Difference | Cohen’d Effect Size | P | Q |
| --- | --- | --- | --- | --- | --- | --- | --- | --- |
| Ankle | Maximal Dorsiflexion (°) | 20.52±5.87 | 18.22±4.51 | [1.02, 3.58] | 2.30 | 0.44 [-0.11, 0.99] | 0.011 | 0.020 |
|  | Maximal Plantarflexion (°) | -18.56±8.58 | -12.04±3.75 | [-8.14, -4.91] | -6.53 | 0.99 [0.41, 1.57] | 0.001 | 0.003 |
|  | Range of Motion (°) | 39.09±7.42 | 30.26±5.70 | [7.21, 10.44] | 8.83 | 1.34 [0.74, 1.94] | 0.001 | 0.003 |
|  | Peak Moment (N·m/kg) | -2.39±0.34 | -2.68±0.42 | [0.10, 0.48] | 0.29 | 0.83 [0.26, 1.40] | 0.004 | 0.009 |
| Knee | Maximal Flexion (°) | 46.65±4.08 | 44.10±5.87 | [-0.06, 5.17] | 2.55 | 0.50 [-0.05, 1.05] | 0.046 | 0.065 |
|  | Minimum Flexion (°) | 17.82±5.00 | 16.61±5.04 | [-1.35, 3.78] | 1.21 | 0.26 [-0.29, 0.81] | 0.073 | 0.093 |
|  | Range of Motion (°) | 28.82±3.22 | 27.49±3.54 | [-0.14, 2.82] | 1.34 | 0.51 [-0.04, 1.06] | 0.075 | 0.094 |
|  | Peak Moment (N·m/kg) | 3.44±0.56 | 3.59±0.51 | [-0.42, 0.12] | -0.15 | 0.31 [-0.24, 0.86] | 0.273 | 0.287 |
| Metatarsophalangeal | Maximal Dorsiflexion (°) | 36.30±5.38 | 29.92±4.49 | [5.17, 7.59] | 6.38 | 1.29 [0.69, 1.89] | 0.001 | 0.002 |
|  | Minimum Dorsiflexion (°) | 9.01±2.31 | 7.53±1.47 | [1.01, 1.96] | 1.49 | 0.77 [0.21, 1.33] | 0.001 | 0.003 |
|  | Range of Motion (°) | 27.28±5.42 | 22.39±3.94 | [3.74, 6.05] | 4.89 | 1.03 [0.45, 1.61] | 0.001 | 0.001 |
|  | Peak Moment (N·m/kg) | -0.32±0.06 | -0.36±0.08 | [0.02, 0.06] | 0.04 | 0.80 [0.24, 1.37] | 0.003 | 0.007 |
| Subtalar | Maximal Dorsiflexion (°) | 8.67±1.39 | 8.33±1.27 | [0.01, 0.66] | 0.34 | 0.26 [-0.29, 0.81] | 0.042 | 0.066 |
|  | Minimum Dorsiflexion (°) | 0.18±0.60 | 0.29±0.50 | [-0.25, 0.02] | -0.11 | 0.20 [-0.35, 0.75] | 0.097 | 0.114 |
|  | Range of Motion (°) | 8.49±1.67 | 8.34±1.44 | [0.07, 0.83] | 0.45 | 0.10 [-0.44, 0.64] | 0.22 | 0.249 |
|  | Peak Moment (N·m/kg) | -1.19±0.17 | -1.27±0.17 | [0.03, 0.12] | 0.08 | 0.57 [0.02, 1.12] | 0.036 | 0.061 |
| Plantar fascia force | Peak Force (BW) | 2.83±0.34 | 3.01±0.34 | [-0.26, -0.08] | -0.17 | 0.71 [0.15, 1.27] | 0.001 | 0.002 |
| Achilles tendon force | Peak Force (BW) | 5.58±0.98 | 6.21±0.94 | [-0.87, -0.40] | -0.63 | 0.66 [0.10, 1.22] | 0.001 | 0.002 |
| Tibial impact force | Peak Force (BW) | 4.05±0.54 | 4.14±0.67 | [-0.29, 0.11] | -0.09 | 0.25 [-0.29, 0.80] | 0.383 | 0.396 |
| Patellofemoral force | Peak Force (BW) | 2.85±0.41 | 3.05±0.49 | [-0.33, -0.06] | -0.20 | 0.83 [0.26, 1.40] | 0.005 | 0.012 |

Note: “*” represents significance with p < 0.05.

**Table E**. Detailed results of the CL, CD and PFF between the healthy and HV groups.

| Variables | | Health Group | HV Group | Difference 95% Confidence Interval | Mean Difference | Cohen’d Effect Size | P | | Q |
| --- | --- | --- | --- | --- | --- | --- | --- | --- | --- |
| Plantar fascia force | CL (N·s/km) | 192663.4±15645.6 | 208285.4±15212.3 | [6557.36, 25455.99] | -15622 | 0.92 [0.35, 1.49] | | **0.001** | 0.004 |
|  | CD (N·(s/km)^1/9.3^) | 3097.3±215.4 | 3286.9±181.6 | [12.83, 335.61] | -189.6 | 1.09 [0.50, 1.68] | | **0.035** | 0.060 |
|  | PFF (%) | 4.3±0.7 | 4.2±1.0 | [-0.063, 0.054] | 0.1 | 0.10 [-0.44, 0.64] | | 0.878 | 0.878 |
| Achilles tendon force | CL (N·s/km) | 325554.7±22473.9 | 363555.3±27435.2 | [63154.00, 89751.05] | -38000.6 | 1.59 [0.96, 2.22] | | **0.001** | 0.004 |
|  | CD (N·(s/km)^1/9.3^) | 5911.5±526.3 | 6584.0±700.7 | [310.72, 1065.11] | -672.5 | 0.99 [0.41, 1.57] | | **0.001** | 0.003 |
|  | PFF (%) | 4.5±0.9 | 5.2±1.2 | [0.0018, 0.1362] | -0.7 | 0.58 [0.03, 1.13] | | **0.044** | 0.066 |
| Tibial impact force | CL (N·s/km) | 287102.7±18526.3 | 298811.3±35630.6 | [-4423.93, 27840.95] | -11708.6 | 0.40 [-0.15, 0.95] | | 0.153 | 0.229 |
|  | CD (N·(s/km)^1/7^) | 5115.9±406.2 | 5265.7±571.0 | [-177.12, 422.93] | -149.8 | 0.28 [-0.27, 0.83] | | 0.415 | 0.498 |
|  | PFF (%) | 4.4±0.8 | 4.8±1.3 | [-0.02293, 0.10277] | -0.4 | 0.35 [-0.20, 0.90] | | 0.208 | 0.277 |
| Patellofemoral force | CL (N·s/km) | 134413.4±10544.2 | 144630.4±11779.9 | [3414.94, 16788.19] | -10217 | 0.85 [0.28, 1.42] | | **0.004** | 0.012 |
|  | CD (N·(s/km)^1/7^) | 3284.0±271.6 | 3513.6±240.9 | [-24.48, 460.59] | -229.6 | 0.53 [-0.02, 1.08] | | 0.077 | 0.103 |
|  | PFF (%) | 4.6±0.8 | 5.0±1.0 | [-0.0239, 0.0966] | -0.4 | 0.37 [-0.18, 0.92] | | 0.231 | 0.277 |

Note: CL: Cumulative load; CD: Cumulative damage; PFF: Probability of fatigue failure; “*” represents significance with p < 0.05.

**Table F**. Detailed results of the correlation coefficients between HVA, FPI and CL, CD, PFF.

|  |  |  | | Health Group | | | | | | |  |  |  | HV Group | | | | | | | | | |
| --- | --- | --- | --- | --- | --- | --- | --- | --- | --- | --- | --- | --- | --- | --- | --- | --- | --- | --- | --- | --- | --- | --- | --- |
|  |  | HVA (9.9° ± 3.0°) | | | |  | FPI (1.8° ± 1.7°) | | | |  |  | HVA (23.8°±5.1°) | | | | |  | | FPI (5.9° ± 1.8°) | | | |
| Variables | | r | R^2^ | | P | Q | r | | R^2^ | P | Q | r | R^2^ | P | Q | r | | | R^2^ | | P | Q |  |
| Plantar fascia force | CL (N·s/km) | 0.39 | 0.1542 | | 0.0472 | 0.078 | | 0.23 | 0.055 | 0.2487 | 0.312 | 0.71 | 0.5064 | 0.0001 | <0.001 | | 0.66 | | 0.4299 | | 0.0001 | <0.001 |  |
|  | CD (N·(s/km)^1/9.3^) | 0.46 | 0.2124 | | 0.0178 | 0.034 | | 0.36 | 0.1279 | 0.0729 | 0.102 | 0.72 | 0.5115 | 0.0001 | <0.001 | | 0.61 | | 0.3768 | | 0.0002 | <0.001 |  |
|  | PFF (%) | 0.49 | 0.2373 | | 0.0116 | 0.023 | | 0.40 | 0.164 | 0.0401 | 0.061 | 0.65 | 0.4245 | 0.0002 | <0.001 | | 0.62 | | 0.3816 | | 0.0001 | <0.001 |  |
| Achilles tendon force | CL (N·s/km) | **0.79** | 0.6218 | | 0.0001 | <0.001 | | 0.60 | 0.3598 | 0.0012 | 0.003 | 0.83 | 0.6808 | 0.0001 | <0.001 | | 0.68 | | 0.4677 | | 0.0001 | <0.001 |  |
|  | CD (N·(s/km)^1/9.3^) | **0.75** | 0.5629 | | 0.0001 | <0.001 | | 0.56 | 0.3136 | 0.0029 | 0.006 | 0.85 | 0.7158 | 0.0001 | <0.001 | | 0.76 | | 0.5798 | | 0.0001 | <0.001 |  |
|  | PFF (%) | 0.66 | 0.4382 | | 0.0002 | <0.001 | | 0.58 | 0.342 | 0.0017 | 0.004 | 0.82 | 0.6775 | 0.0001 | <0.001 | | 0.74 | | 0.546 | | 0.0001 | <0.001 |  |
| Tibial impact force | CL (N·s/km) | 0.51 | 0.259 | | 0.0079 | 0.015 | | 0.26 | 0.0669 | 0.2019 | 0.236 | 0.47 | 0.2178 | 0.0089 | 0.017 | | 0.29 | | 0.0835 | | 0.227 | 0.259 |  |
|  | CD (N·(s/km)^1/7^) | 0.53 | 0.276 | | 0.0059 | 0.012 | | 0.26 | 0.0675 | 0.1999 | 0.234 | 0.46 | 0.2107 | 0.0094 | 0.018 | | 0.28 | | 0.0808 | | 0.219 | 0.251 |  |
|  | PFF (%) | 0.33 | 0.1078 | | 0.1014 | 0.129 | | 0.01 | 0.0002 | 0.9483 | 0.948 | 0.43 | 0.1836 | 0.0239 | 0.041 | | 0.27 | | 0.0738 | | 0.206 | 0.238 |  |
| Patellofemoral force | CL (N·s/km) | 0.59 | 0.3465 | | 0.0016 | 0.004 | | 0.58 | 0.3361 | 0.0019 | 0.004 | 0.69 | 0.4801 | 0.0001 | <0.001 | | 0.55 | | 0.3079 | | 0.0028 | 0.006 |  |
|  | CD (N·(s/km)^1/7^) | 0.61 | 0.3739 | | 0.0009 | 0.002 | | 0.56 | 0.3134 | 0.0029 | 0.006 | 0.61 | 0.3776 | 0.0003 | <0.001 | | 0.54 | | 0.2864 | | 0.0062 | 0.012 |  |
|  | PFF (%) | 0.64 | 0.4092 | | 0.0004 | 0.001 | | 0.59 | 0.3458 | 0.0016 | 0.004 | 0.59 | 0.3483 | 0.0007 | 0.002 | | 0.50 | | 0.2491 | | 0.0074 | 0.015 |  |

Note: PFF: CL: Cumulative load; CD: Cumulative damage; PFF: Probability of fatigue failure; “*” represents significance with p < 0

**Table G**. The mean value and standard deviation of R^2^, RMSE, NRMSE from each model

| Models | | R^2^ | RMSE | NRMSE |
| --- | --- | --- | --- | --- |
| BiLSTM | Plantar fascia CD | 0.8247 (0.005) | 195.74 (109.01) | 0.45 (0.25) |
|  | Plantar fascia PFF | 0.8783 (0.006) | 0.97 (0.81) | 0.26 (0.22) |
|  | Achilles tendon CD | 0.8759 (0.008) | 433.34 (350.82) | 0.27 (0.22) |
|  | Achilles tendon PFF | 0.8417 (0.007) | 1.33 (1.25) | 0.26 (0.25) |
|  | Tibial CD | 0.8306 (0.004) | 340.74 (244.18) | 0.35 (0.24) |
|  | Tibial PFF | 0.8222 (0.006) | 1.12 (1.07) | 0.19 (0.18) |
|  | Patellofemoral CD | 0.8510 (0.007) | 236.17 (164.88) | 0.31 (0.21) |
|  | Patellofemoral PFF | 0.8370 (0.006) | 1.05 (1.04) | 0.23 (0.20) |
| CNN–BiLSTM | Plantar fascia CD | 0.8435 (0.005) | 167.45 (115.07) | 0.39 (0.27) |
|  | Plantar fascia PFF | 0.8565 (0.004) | 0.78 (0.57) | 0.21 (0.15) |
|  | Achilles tendon CD | 0.8894 (0.006) | 384.25 (273.79) | 0.24 (0.17) |
|  | Achilles tendon PFF | 0.8547 (0.005) | 1.06 (0.82) | 0.21 (0.16) |
|  | Tibial CD | 0.8465 (0.004) | 360.40 (285.55) | 0.37 (0.29) |
|  | Tibial PFF | 0.8540 (0.007) | 0.68 (0.49) | 0.11 (0.08) |
|  | Patellofemoral CD | 0.8767 (0.006) | 231.03 (175.68) | 0.31 (0.23) |
|  | Patellofemoral PFF | 0.8538 (0.005) | 1.11 (0.88) | 0.25 (0.20) |
| SSO–CNN–BiLSTM | Plantar fascia CD | 0.8768 (0.004) | 140.71 (110.01) | 0.33 (0.25) |
|  | Plantar fascia PFF | 0.8932 (0.002) | 0.72 (0.56) | 0.20 (0.15) |
|  | Achilles tendon CD | 0.9054 (0.005) | 375.51 (277.54) | 0.24 (0.18) |
|  | Achilles tendon PFF | 0.8757 (0.003) | 1.03 (0.91) | 0.20 (0.18) |
|  | Tibial CD | 0.8649 (0.005) | 261.67 (180.73) | 0.27 (0.19) |
|  | Tibial PFF | 0.9172 (0.005) | 0.52 (0.45) | 0.08 (0.07) |
|  | Patellofemoral CD | 0.8664 (0.002) | 215.82 (158.66) | 0.29 (0.21) |
|  | Patellofemoral PFF | 0.8891 (0.004) | 1.08 (0.81) | 0.24 (0.18) |
| SSO–CNN–BiLSTM-HAM | Plantar fascia CD | 0.8995 (0.003) | 133.47 (91.02) | 0.30 (0.21) |
|  | Plantar fascia PFF | 0.9269 (0.002) | 0.48 (0.45) | 0.13 (0.12) |
|  | Achilles tendon CD | 0.9324 (0.002) | 301.12 (213.98) | 0.19 (0.13) |
|  | Achilles tendon PFF | 0.9080 (0.003) | 0.64 (0.57) | 0.13 (0.11) |
|  | Tibial CD | 0.8896 (0.004) | 286.69 (197.23) | 0.30 (0.20) |
|  | Tibial PFF | 0.9302 (0.002) | 0.46 (0.37) | 0.08 (0.06) |
|  | Patellofemoral CD | 0.9081 (0.003) | 166.77 (109.21) | 0.22 (0.15) |
|  | Patellofemoral PFF | 0.8893 (0.004) | 0.61 (0.53) | 0.14 (0.12) |

Note: CD: Cumulative damage; PFF: Probability of fatigue failure
