# Supplementary material for: Structure-aware fatigue modeling in foot deformities: A digital health framework for tissue-specific running injury risk prediction using multi-modal data
Source: PLOS Digit Health. 2026 Jul 10;5(7):e0001537. doi: 10.1371/journal.pdig.0001537 (PMC13354006; doi:10.1371/journal.pdig.0001537)
Supplement: S3 File — (DOCX) [file pdig.0001537.s003.docx]

**Supplementary Materials**

**Structure-Aware Fatigue Modeling in Foot Deformities: A Digital Health Framework for Tissue-Specific Running Injury Risk Prediction Using Multi-Modal Data**

Zhifeng Zhou^a, #^, Huiyu Zhou^a, #^, Datao Xu^a*^, Bas Van Hooren^b^, Yi Yuan^c^**^*^**, Tianle Jie^a,d^ Xiangli Gao^a,e^, Xiuye Qu^a^, Zanni Zhang^a^, Zixiang Gao^f^, Liangliang Xiang^g^, Yaodong Gu^a*^

^a^ Faculty of Sports Science, Ningbo University, Ningbo 315211, China

^b^ Department of Nutrition and Movement Sciences, NUTRIM Institute of Nutrition and Translational Research in Metabolism, Maastricht University Medical Centre+, Universiteitssingel 50, Maastricht, NL 6229 ER, The Netherlands

^c^ Research Academy of Medicine Combining Sports, Ningbo No. 2 Hospital, Ningbo 315010, China

^d^ Human Performance Laboratory, Faculty of Kinesiology, University of Calgary, Calgary, AB, Canada

^e^ The Doctoral School on Safety and Security Sciences, Óbuda University, Budapest, Hungary

^f^ Research Center for Molecular Exercise Science, Hungarian University of Sports Science, Budapest

^g^ KTH Move Ability Lab, Department of Engineering Mechanics, KTH Royal Institute of Technology, SE-100 44 Stockholm, Sweden

***Correspondence:**

Datao Xu (xudatao@nbu.edu.cn); Yaodong Gu (guyaodong@nbu.edu.cn).

Address: Faculty of Sports Science, Ningbo University, No. 818, Fenghua Road, Jiangbei District, Ningbo, Zhejiang, China. 315211

Tel: +86(0)574 87609369

**This Supporting Material file includes:**

**1.** **Section A: Subject-Specific Foot-Ankle Musculoskeletal Model Creation**

**2. Section B: Experimental Protocol and Data Collection**

**3. Section C:** **Achilles Tendon and Plantar Fascia Model Creation and Property Setting**

**4. Section D: Lower Limb Tissue Mechanical Fatigue and Cumulative Injury**

**5. Section E: Model Validation**

**6. Section F: Data-Driven Deep Learning Model Estimates for Lower Extremity Injury Risk Modeling**

**7. SI References**

**1. Section A:** **Subject-Specific Foot-Ankle Musculoskeletal Model Creation**

**1.1 Overall Process of Modeling**

This work developed a subject-specific foot-ankle musculoskeletal model containing 32 muscles and 36 ligaments to calculate the load mechanics of the lateral collateral ligaments. The whole process of model construction is shown in **Fig S1**. The current study combines medical imaging data to construct the subject-specific ankle musculoskeletal model, which takes into account the subject's individualized stiffness characteristics as well as ligamentous attributes to more closely match the real situation of the human body. Furthermore, when considering the loading mechanism of the ligament, we constructed a SCM to restore the NLSTV properties of the dense connective tissue of the ligament. This can more realistically revert the LLM and reveal the foot-ankle mechanical properties during running [1, 2].

This work captured MRI and CT data of the subjects' right foot-ankle in the neutral position (NP) state. During foot-ankle medical imaging data acquisition, subjects were asked to lie supine on the device in a naturally relaxed state, and an ankle immobilization brace was used to make the foot perpendicular to the tibial shaft, and the position was identified as neutral [3]. The scanning area was determined as the whole foot from 5 cm of the distal tibia near the ankle joint. For the MRI scan (3.0 T magnet, Siemens, Germany), the MRI imaging of the foot-ankle on an MRI scanner using a surface coil and a 3D spoiled phase gradient recalled acquisition in steady-state (3D SPGR) sequence (T1-weighted turbo spin echo; 0.5 mm slice thickness; 3.3 mm slice spacing; resolution of 1024×1024 pixels). For the CT scan (Siemens ltd, Somatom, Berlin, Germany), the scan was done with 0.5 mm in thickness and no gap between the scans (tube voltage 120 kV; tube current 125 mA; image matrix 512×512 voxels).

**1.2 Body Segments Extraction and Anthropometric Properties Computation**

The acquired foot and ankle CT and MRI data were first imported into Mimics (version 21.0, Materialise, Leuven, Belgium) to segment and extract the body segments (bones and associated soft tissues) and to create a baseline model. In this procedure, the current work segments the approximate range of bones and associated soft tissues by adjusting the threshold in the range of 1250 ~ 3120 Hounsfield units (HU) [4]. After that, the more difficult contours (the bone close to the articular surface and the relatively thin muscles and soft tissues attached to the bone) were determined manually by an experienced experimenter. Meanwhile, this study used the corresponding Mimics Innovation Suite to determine the grayscale values (HU) of each body segment, and based on the linear relationship between them to calculate the density values $\rho$ of each volume grid element [5]. Each segment $i$ was set to 10 different density gradients, each associated with equally spaced gray values:

$$\rho_{n,i}=b_{i}+a_{i}\times HU_{n} (1)$$

Where the slope $a$ and the constant term $b$ in the linear relationship equation are solved from the density values of bone trabeculae and bone cortex. Based on previous studies, we assumed that the bone trabeculae and the bone cortex were the minimum and maximum gray values for each segment, respectively [5], so there are:

$$\left\{ \begin{aligned} \rho_{Trabecular}=b_{i}+a_{i}\times HU_{{min}_{i}} \\ \rho_{Cortical}=b_{i}+a_{i}\times HU_{{max}_{i}} \end{aligned} \right. (2)$$

Where the $\rho_{Trabecular}$ takes 0.05×10^3^ kg/m^3^, and the $\rho_{Cortical}$ takes 1.75×10^3^ kg/m^3^ [36, 4]. The bone density value $\rho_{B}$ was solved for each body segment based on the determined $b$ and $a$. For the soft tissue density values of each body segment, we used a uniform density based on average muscle density and fat density: $\rho_{ST}$ 1.016×10^3^ kg/m^3^ [6, 7]. Based on the solved density data, we used an explicit formulation of the rotational inertia of a 3D tetrahedron as a polynomial in the vertex coordinates to compute the inertia tensor [5, 8]. The inertia tensor was calculated using density information using explicit formulas for the moments of inertia of three-dimensional tetrahedrons as polynomials of their vertex coordinates. The center of the body segment $D$ was set to $Q$. Define the inertia tensor $E_{Q}$ in the x, y, and z-axes of the center $Q$ to be:

$$E_{Q}=\left( \begin{matrix} a & -b^{'} & -c^{'} \\ -b^{'} & b & -a^{'} \\ -c^{'} & -a^{'} & c \end{matrix} \right) (3)$$

where $a$, $b$, and $c$ represent the moments of inertia concerning the x, y, and z-axes, respectively, as follows:

$$\left\{ \begin{matrix} a=\int\rho_{B}(y^{2}+z^{2})dD \\ b=\int\rho_{B}(x^{2}+z^{2})dD \\ c=\int\rho_{B}(x^{2}+y^{2})dD \end{matrix} \right. (4)$$

$a^{'}$, $b^{'}$, and $c'$ are the inertial products with respect to the x, y, and z axes, respectively:

$$\left\{ \begin{matrix} a^{'}=\int\rho_{B}yzdD \\ b^{'}=\int\rho_{B}xzdD \\ c^{'}=\int\rho_{B}xydD \end{matrix} \right. (5)$$

Where the principal directions are the eigenvectors of the inertia tensor $E_{Q}$ and the eigenvalues $E_{Q}$ are the principal moments of inertia [8]. The x-axis direction was set as the principal direction according to previous studies, and the principal direction of the inertia tensor $E_{Q}$ was taken to be 0.001 kg/m^2^ [5, 8]. This can effectively avoid the problems of computational efficiency, numerical stability, and physical reasonableness during the dynamic simulation of the musculoskeletal model [9, 10].

**1.3 Subject-Specific Ankle-Foot Model Development**

The extracted foot segments were imported into NMSBuilder (Version 2.1, Rizzoli Orthopedic Institute, Bologna, Italy) in STL file format, and the baseline model data was organized into a hierarchical structure [11]. The uniform density of bone and soft tissue was assigned to each segment as a metadata attribute to calculate the inertial properties (mass, centroid, and moment of inertia) of each segment [12, 13]. The calculated inertial characteristics data were stored in the OpenSim (Version 4.4, Stanford University, Stanford, USA) model VME. The anatomical landmark reference system of the foot was created based on the existing landmark set in NMSBuilder, and each joint of the foot contained two reference coordinate systems [11]. Based on the definition of the foot coordinate system proposed by the International Society of Biomechanics, the link reference coordinate system for each individual segment was established (**Section 1.4**) [5, 14].

The input muscle landmark cloud was initially registered to the target bone landmark cloud via affine registration [11]. For the few landmarks that were not successfully paired, they were paired to the corresponding muscle volumes using the centroid method [15]. The paired completed muscle landmarks were given coherent names and subsequently OpenSim muscle objects were created. In order to initially define the geometric path (origin, insertion, and intermediate passage points) of the muscle ligament unit, the current work is based on the automatically created path landmarks (**Table S2**). The import landmark refers to the coordinate position of the KU Leuven foot model [5, 16].

According to the previous study by Delp et al., the current work assumes that muscle fibers are proportional to tendon length [10, 17], and the maximum isometric force $F_{\max i}$ of each tendon unit $i$ is estimated according to the following equation:

$$M_{PCAS}=\frac{M_{Vol}}{M_{l_{0}}}=\frac{M_{Vol}}{M_{l_{0}^{(gen)}}\times\frac{M_{l_{mt}}}{M_{l_{mt}^{(gen)}}}} (6)$$

Where the $M_{Vol}$ represents the corresponding muscle volume, which is calculated from the MRI data. The $M_{l_{0}}$ and $M_{l_{mt}}$ represent the optimal length of muscle fiber and muscle tendon for the developed subject-specific foot model, respectively. The $M_{l_{0}^{(gen)}}$ and $M_{l_{mt}^{(gen)}}$ represent the corresponding quantities for the general model of the reference study [10, 17]. Therefore, the final formula for $F_{\max i}$ is:

$$F_{\max i}=\left( \frac{M_{Vol}}{M_{l_{0}^{(gen)}}\times\frac{M_{l_{mt}}}{M_{l_{mt}^{(gen)}}}} \right)_{i}\times\sigma(7)$$

The calculated maximum isometric force values were then assigned to each muscle-tendon unit as a metadata attribute. Finally, we generated and compiled C++ commands for the OpenSim application programming interface (API) to create a baseline OpenSim model (*.osim file*).

**1.4 Link Reference Coordinate System Establishment**

Based on the definition rules of the foot coordinate system proposed by the International Society of Biomechanics, this study divides the foot into five regions (**Fig S2**): calcaneus, talus, midfoot (scaphoid, cuboid, cuneiform), forefoot (first to fifth metatarsal bones), and toes (toe bones) [5, 14]. The distal tibial-fibular segments were determined based on the KU Leuven foot model [5, 16]. Therefore, the current study defines six-coordinate systems to simulate foot motion: tibia-fibula coordinate system, talus coordinate system, heel coordinate system, midfoot coordinate system, forefoot coordinate system, and toe coordinate system [5, 14]. These six-coordinate systems define the 5 foot-ankle joints: the ankle, subtalar, transverse tarsal, metatarsal tarsal, and metatarsophalangeal joints. Each joint defines three degrees of freedom, and the definition of these three rotational axes was modeled and analyzed in MATLAB (Visual R2023a, MathWorks, USA) and developed in OpenSim.

The ankle plantar dorsiflexion $A_{D-P}$ (Z-axis) is set at the angle of 80° relative to the sagittal plane and 84° to the foot midline [18-20]. The ankle internal rotation-external rotation $A_{Ir-Er}$ (Y-axis) is set at an angle of 38° relative to the horizontal plane, and the medial inclination of 21° to the midline of the foot [18-20]. The ankle inversion-eversion $A_{Iv-Ev}$) (X-axis) is set to be perpendicular to the tibial axis [18, 19]. The three rotation axes are defined in the form of unit vectors:

$$\left\{ \begin{aligned} A_{D-P}=(0.1045, 0.1387, -0.9848) \\ A_{Ir-Er}=(0, -1, 0) \\ A_{Iv-Ev}=(-0.7018, -0.6157, 0.3584) \end{aligned} \right. (8)$$

For the subtalar joint, its spatial positioning was 16°, 42° and 48° from the sagittal, frontal and transverse plane respectively [21, 22]. The middle tarsal joint is mainly connected with the calcaneocuboid joint and talonavicular joint, which point to the oblique and longitudinal axes of the foot in space. The oblique axis is aligned at 57° from the sagittal plane and 53° from the cross-section, respectively. The longitudinal axis is aligned with 11° from the sagittal plane and 16° from the cross-section, respectively [4, 5]. The tarsometatarsal joint mainly connects the tarsal bones and metatarsal bones, and is spatially aligned with the first and fifth ray axes of the foot. The first ray is located at 15°, 5°, and 10° from the sagittal, frontal, and transverse planes, respectively. The fifth ray is located at 35°, 20° from the sagittal, and transverse planes, respectively [4, 23]. The metatarsophalangeal joint mainly connects the phalanx and metatarsal bones. Meanwhile, the hinge joints were constructed to simulate interphalangeal rotation. The semi-cylindrical structure was used to construct the inner and outer foot arcs, and its spatial positioning was 25°, 0° and 89° from the sagittal, frontal and transverse planes, respectively [4].

Take the transfer transformation of the tibia-fibula coordinate system relative to the talus coordinate system and the calcaneus coordinate system as an example. In the ankle joint construction, the three rotation axes for ankle dorsiflexion, plantarflexion (sagittal), inversion, eversion (frontal), internal rotation and external rotation (horizontal) are $A_{D-P}$, $A_{Iv-Ev}$, $A_{Ir-Er}$. The three independent position variables are denoted as $\theta_{D-P}, \theta_{Ir-Er}, \theta_{Iv-Ev}$. The tibia-fibula coordinate system is denoted as ${X-Y-Z}_{Tib}$, the talus coordinate system is denoted as ${X-Y-Z}_{Tal}$, and the calcaneus coordinate system is denoted as ${X-Y-Z}_{Cal}$. Taking the tibia-fibula coordinate system ${X-Y-Z}_{Tib}$ as the main coordinate system, the origin of ${X-Y-Z}_{Tal}$ relative to ${X-Y-Z}_{Tib}$ and ${X-Y-Z}_{Cal}$ relative to ${X-Y-Z}_{Tal}$ are obtained through two anatomical translations of the ankle joint. For the translation matrix 1 $M_{3\times1}^{Tib-Tal}=(0, 430, 0)$, the coordinates of any point in this coordinate system are expressed as:

$$P_{3\times1}^{Tib}=R_{3\times3}\left( \theta_{D-P}, \theta_{Ir-Er}, \theta_{Iv-Ev} \right)\times P_{3\times1}^{Tal}+M_{3\times1}^{Tib-Tal}$$

For the translation matrix 2 $M_{3\times1}^{Tal-Cal}=(-48.77, -41.95, 7.92)$, the coordinates of any point in this coordinate system are expressed as:

$$P_{3\times1}^{Tib}=R_{3\times3}\left( \theta_{D-P}, \theta_{Ir-Er}, \theta_{Iv-Ev} \right)\times P_{3\times1}^{Cal}+M_{3\times1}^{Tib-Tal}+M_{3\times1}^{Tal-Cal}$$

Where $R$ represents the rotation matrix of the foot at any point:

$$R={(X_{1}+\cos\theta_{D-P}\times\left( I_{3\times3}-X_{1} \right)+\sin\theta_{D-P}\times X_{2})}^{T}$$

Where $I_{3\times3}$, $X_{1}$, $X_{2}$ are defined as

$$\left\{ \begin{aligned} I_{3\times3}=\binom{A_{D-P}}{\begin{aligned} A_{Ir-Er} \\ A_{Iv-Ev} \end{aligned}} \\ X_{1}=\left( {A_{D-P}(1)A_{D-P}(1) \atop\begin{aligned} A_{D-P}(2)A_{D-P}(1) \\ A_{D-P}(3)A_{D-P}(1) \end{aligned}} {{A_{D-P}(1)A}_{D-P}(2) \atop\begin{aligned} {A_{D-P}(2)A}_{D-P}(2) \\ {A_{D-P}(3)A}_{D-P}(2) \end{aligned}} {{A_{D-P}(1)A}_{D-P}(3) \atop\begin{aligned} {A_{D-P}(2)A}_{D-P}(3) \\ {A_{D-P}(3)A}_{D-P}(3) \end{aligned}} \right) \\ X_{2}=\left( {0 \atop\begin{aligned} A_{D-P}(3) \\ -A_{D-P}(2) \end{aligned}} {-A_{D-P}(3) \atop\begin{aligned} 0 \\ A_{D-P}(1) \end{aligned}} {A_{D-P}(2) \atop\begin{aligned} {-A}_{D-P}(1) \\ 0 \end{aligned}} \right) \end{aligned} \right. (9)$$

**1.5 Spatial Coordinate System Alignment of Model**

To make sure that the calculations were correct, this study converted the coordinate system of the foot body segments exported by NMSbuilder through the MATLAB-OpenSim application programming interface (API) into a coordinate system alignment suitable for use under OpenSim. We adopted the Euclidean transform in 3-D space based on singular value decomposition (SVD) to realize the alignment [24, 25]. As an effective matrix decomposition algorithm, SVD can significantly reduce the dimension of the feature space, obtain unique and stable feature descriptions, and improve the accuracy of the spatial transformation of the coordinate system [24]. We determined four markers of the foot body segments for coordinate system alignment: the tip of the medial and lateral malleolus, and the tip of the 1st and 5th distal metatarsals. The alignment transformation between two coordinate systems consists of a rotation plus a translation.

Taking one of the markers as an example, the vector consisting of that marker point and the coordinate system is defined as $p$. The original coordinate system of this marked point is defined as $O_{m}$, and its unit orthogonal basis is ${(e}_{1m}, e_{2m},e_{3m})$, and the coordinate under this coordinate system is $p_{m}=\left[ \begin{matrix} p_{1m} & p_{2m} & p_{3m} \end{matrix} \right]^{T}$. The original coordinate system based on the foot model in OpenSim is defined as $O_{o}$, and its unit orthogonal basis is ${(e}_{1o}, e_{2o},e_{3o})$, and the coordinate under this coordinate system is ${p_{o}=\left[ \begin{matrix} p_{1o} & p_{2o} & p_{3o} \end{matrix} \right]}^{T}$, and $p_{m}=R_{mo}p_{o}$.

The process of rotating the coordinate system $O_{m}$ alignment to the coordinate system $O_{o}$ is executed by first solving the $O_{m}$ to $O_{o}$ rotation matrix $R_{mo}$:

$$R_{mo}=\left[ \begin{matrix} e_{1m}^{T}e_{1o} & e_{1m}^{T}e_{2o} & e_{1m}^{T}e_{3o} \\ e_{2m}^{T}e_{1o} & e_{2m}^{T}e_{2o} & e_{2m}^{T}e_{3o} \\ e_{3m}^{T}e_{1o} & e_{3m}^{T}e_{2o} & e_{3m}^{T}e_{3o} \end{matrix} \right] (10)$$

Where the $e_{1m}^{T}e_{1o}$ is the cosine angle between the vectors $e_{1m}$and $e_{1o}$, and $R_{mo}$ is the orthogonal rotation matrix with determinant 1. The set of 3-D rotation matrices is called the special orthogonal group:

$$SO\left( 3 \right)=\left\{ R\in\mathbb{R}^{3\times3} | RR^{T}=I,\det R)=1 \right\} (11)$$

Then, the translation process of the coordinate system $O_{m}$ registration to coordinate the system $O_{o}$ is performed:

$$p_{m}=R_{mo}p_{o}+t_{mo}$$

Where $t_{mo}$ is the coordinate under the coordinate system $O_{m}$ of the vector from the origin of the coordinate system $O_{m}$ to the origin of the coordinate system $O_{o}$. Convert the above Euclidean transformation formula into homogeneous form:

$$\left[ \begin{matrix} p_{m} \\ 1 \end{matrix} \right]=\left[ \begin{matrix} R_{mo} & t_{mo} \\ 0^{T} & 1 \end{matrix} \right]\left[ \begin{matrix} p_{c} \\ 1 \end{matrix} \right]+\left[ \begin{matrix} t_{mo} \\ 1 \end{matrix} \right]=T_{mo}\left[ \begin{matrix} p_{c} \\ 1 \end{matrix} \right]+\left[ \begin{matrix} t_{mo} \\ 1 \end{matrix} \right] (12)$$

Where $T_{mo}$ is the transformation matrix from the coordinate system $O_{m}$ to $O_{o}$. The set formed by such transformation matrices becomes the special Euclidean group $SE\left( 3 \right)$:

$$SE\left( 3 \right)=\left\{ T=\left[ \begin{matrix} R & t \\ 0^{T} & 1 \end{matrix} \right]\in\mathbb{R}^{4\times4}|R\in SO\left( 3 \right),t\in\mathbb{R}^{3} \right\} (13)$$

Therefore, solving the registration transformation $(R_{mo},t_{mo})$ as:

$$\left( R_{mo},t_{mo} \right)=\min_{(R,t)\in SE\left( 3 \right)} \left\| p_{m}^{'}-{(R_{mo}p_{o}^{'}+t_{mo}E)}^{T} \right\|_{F} (14)$$

Where $E$ is the unit matrix and $p_{m}^{'}$ and $p_{o}^{'}$ are the sets of coordinates of $p_{m}$ and $p_{o}$ transformed by rotation, respectively. For the above equation, the SVD method is used to expand, then:

$$p_{m}^{'}{p_{o}^{'}}^{T}=U\Sigma V^{T}\in\mathbb{R}^{3\times3} (15)$$

Where $U$ is the left singular matrix, $V$ is the right singular matrix, and $\Sigma$ is the diagonal matrix containing the singular values. Finally, the matching matrix is defined based on the set matching standard error $\varepsilon^{(j)}$ solves the transformation matrix $R_{mo}$:

$$\varepsilon^{(j)}=\left\| \bar{p}_{m}^{'}-\bar{p}_{o}^{'} \right\|_{F}/N_{4} (16)$$

$$R_{mo}=Vdiag(\left\| p_{m}^{'}-{(R_{mo}p_{o}^{'}+t_{mo}E)}^{T} \right\|_{F})U^{T} (17)$$

**1.6 Marker Set Protocol and Acquisition**

As shown in **Fig S3**, this study captured the subject's motion through reflective marker points. Except for the foot, the other marker points refer to the lower limb model developed in our previous study (**Fig S3A**) [26, 27]. As shown in **Fig S3B**, a total of 20 markers were used on the unilateral foot-ankle to capture foot motion. Segment of tibiofibular: medial malleolus (MM), lateral malleolus (LM), peroneal tubercle (PT); Segment of Calcaneus: calcaneal tuberosity (CT), Lateral Process of Calcaneus (LPC), Medial Process of Calcaneus (MPC); Talus: tubercle of the talus (TT), head of talus (HT), medial tubercle of the talus (TMT); Midfoot: tuberosity of cuboid (TC), tuberosity of navicular (TN), first cuneiform (CN1), third cuneiform (CN3); Forefoot: the styloid process of the first metatarsal bone (SMB1), head of the first metatarsal bone (HMB1), styloid process of the fifth metatarsal bone (SMB5), head of the fifth metatarsal bone (HMB5); Toes: first proximal phalanx (FPP), first distal phalanx (FDP), second proximal phalanx (SPP).

As a way to make the marker points more accurate at estimating joint motion, the present study uses the Kalman smoothing algorithm on their spatial coordinate system [28, 29]. The method can take into account the complete marker point trajectories and locate the model in the pose that best matches the experimental marker and coordinate error information. The final goal of minimizing the marker point trajectory error is achieved through weighted least squares [28]. First, the joint kinematics during motion time $t$ are estimated based on the Kalman filter. Then it is recursed backward based on the coordinates of the marker point trajectories to estimate the available information derived under the generalized coordinate system: the complete marker point trajectories, the process model, and the measurement model [30, 31]. The generalized coordinate system $q$ of the joint kinematics and its corresponding $k-th$ derivatives are represented by the vector $X$:

$$X=\left[ \begin{aligned} q_{1}q_{1}^{\left( 1 \right)}\ldots q_{1}^{\left( K \right)} \\ q_{2}q_{2}^{\left( 1 \right)}\ldots q_{2}^{\left( K \right)} \\ \vdots\\ q_{J}q_{J}^{\left( 1 \right)}\ldots q_{J}^{\left( K \right)} \end{aligned} \right]^{T} (18)$$

Where the $q_{j}^{\left( K \right)} (j=1, 2, \ldots,J)$ represents the $k-th$ time derivative of $q_{j}$ at the $j-th$ degree of freedom.

The process model consists of $J$ sub-models that describe the motion of the degrees of freedom, and it is used to solve for the desired time evolution of the joint kinematics $X$ [28, 30]. The $k-th$ derivative of the generalized coordinates, $q_{j}^{\left( K \right)}$, as a constant, and the resulting noise term from the constant term is [28]:

$$\left[ \begin{aligned} q_{j}(t_{i}+\Delta t) \\ q_{j}^{\left( 1 \right)}(t_{i}+\Delta t) \\ \vdots\\ q_{j}^{\left( K \right)}(t_{i}+\Delta t) \end{aligned} \right]=\left[ \begin{aligned} \begin{matrix} 1 & \Delta t \end{matrix} \begin{matrix} \frac{{\Delta t}^{2}}{2} & \frac{{\Delta t}^{K}}{K!} \end{matrix} \\ \begin{matrix} 0 & 1 \end{matrix} \begin{matrix} \Delta t & \frac{{\Delta t}^{K-1}}{\left( K-1 \right)!} \end{matrix} \\ \vdots\\ \begin{matrix} 0 & \end{matrix} \begin{matrix} & 1 \end{matrix} \end{aligned} \right]\left[ \begin{aligned} q_{j}(t_{i}) \\ q_{j}^{\left( 1 \right)}(t_{i}) \\ \vdots\\ q_{j}^{\left( K \right)}(t_{i}) \end{aligned} \right]+n_{j}(t_{i}) (19)$$

Where $t$ is the motion time, $i$ is the time point, $\Delta t$ represents the sample time. The generalized coordinate system with $(K+1)-th$ order derivatives was modeled as zero-averaged Gaussian noise with covariance $\sigma_{K+1, j}^{2}$. After that, the process noise $n_{j}\left( t \right)$ is given by:

$$\left\{ \begin{aligned} n_{j}\left( t \right)=N(0, Q_{j}) \\ Q_{j}=\sigma_{K+1, j}^{2}G^{T}G \\ G=\left[ \frac{{\Delta t}^{K+1}}{\left( K+1 \right)!}\times\frac{{\Delta t}^{K}}{K!}\times\Delta t \right] \end{aligned} \right. (20)$$

Also, the error in the measured position of the marker point in the joint kinematics $X(t)$ is defined as the combination of the noise-free measurement model $h\left( x\left( t \right) \right)$ and the measurement noise $v\left( t \right)$ [28]. Among them, the noise-free measurement model $h\left( x\left( t \right) \right)$ is mainly linked to the proposed biomechanical model. The measurement noise $v\left( t \right)$, which results from the deviation of the marker point's actual measurement value, and the Gaussian distribution, represents the degree of deviation of the measurement position of the marker point.

Meanwhile, considering the high number of marker points placed on the foot, we limit the range of weights assigned to these markers based on the linear assignment method to around 1. Among them, the weight assignment of the foot is mainly based on the relative distance between each marker point in each body segment at each time point. The inter-distance between marker points (IMD) in the same body segment was obtained by subtracting the minimum distance from the calculated maximum distance between marker points, and the average IMD was further calculated [32]. As shown in **Fig S4**, taking the IMD and weight attribution results of the labeled points on the biped of subject 1 in one of the tests as an example, their IMDs were mainly distributed in the range of 2–6 mm, and their weights were mainly distributed in the range of 0.8–1.2. Due to the greater distance between each marker in the upper body and legs, making it easier to capture, they were given a higher weight attribution compared to the foot-related markers.

**2. Section B: Experimental Protocol and Data Collection**

This work used the VICON motion capture device (Vicon Metrics Ltd., UK) with eight infrared cameras and two AMTI force plates (AMTI, Watertown, USA) to capture kinematic and kinetic during the running stance phase, setting the acquisition frequencies at 200 Hz and 1000 Hz, respectively. A wireless EMG device was used to synchronously acquire lower limb muscle activity (1000 Hz). For ten selected muscles, the maximum voluntary contraction (MVC) EMG signal data were acquired to be used as a standard signal to solve for muscle activation [26]. Subjects were familiarized with the experimental procedures, and the formal experiment was conducted after a 10-minute warm-up run. According to the internationally accepted Foot Posture Index guidelines, FPI scores were obtained from talar palpation, lateral ankle aponeurosis, internal and external heel turn, talonavicular synchondrosis, medial longitudinal foot arch, and forefoot adduction (-2/-1/0/1/2) [33]. The FPI score classifies foot posture into five levels: excessive inversion (-12), inversion (-5), normal (0), pronation (5) and excessive pronation (12).

To assess the CL per-kilometer experienced by subjects during running, the current study first required subjects to run 1km continuously at 12 Km/h on a Zebris (Zebris Medical GmbH, Isny, Germany) running platform, followed by gait biomechanics testing. Based on Zebris derived steps per kilometer run by the subjects, they were used to assess the CL. Two force plates were placed in the center of a 20 m runway, and infrared timers were placed in front of and behind the force plates (4 m distance) to measure the subject's running speed. Subjects ran naturally across the track (3.33±10% m/s), in a natural and unconscious manner, stepped their right foot on the first force plate and their left foot on the second force plate [34, 35]. Five successful gait data acquisitions were performed for each subject.

The running stance phase was defined as the foot stepping onto the force plate (VGRF > 10 N) to leaving the force plate [34]. First, the data were processed in the Vicon Nexus 2.1.0 software: (a) intercepting the gait cycle; (b) naming the captured successful reflective markers; (c) repairing the missing markers; (d) deleting the incorrect markers; and (e) connecting the correct markers. Next, we exported the C3D file from Vicon Nexus and imported it into Visual 3D 6.7.3 (C-Motion Inc., Germantown, US) software for inverse kinematics/dynamics modeling. By finding an appropriate signal-to-noise ratio, it was determined that a fourth-order zero-phase lag Butterworth low-pass filter with frequencies of 10 and 20 Hz was used to filter the kinematic and kinetic data, respectively [1, 2]. The processed data were saved in Visual 3D as “*.osim*” format, and then imported into OpenSim 4.4 (Stanford University, Stanford, USA) for further modeling computation. Patellofemoral and ankle contact forces (expressed in the local coordinate system of the proximal tibia and femur) were computed in OpenSim by summing joint forces with dynamically optimized muscle force vectors. Tibial impact (TI) force is defined as the vectorial summation of the ankle contact force and the force of gravity at the center of mass of the distal third of the tibia [33].

For the EMG signal, the raw signals were processed firstly by band-pass filtering with a Butterworth fourth-order filter (frequency range of 10-400 Hz). Then, the full-wave rectification was conducted. Finally, based on the determined most appropriate signal-to-noise ratio, the low-pass filtered with a 6 Hz cut-off frequency was performed [26, 36]. The raw EMG signals were processed firstly by band-pass filtering with a Butterworth fourth-order filter (frequency range of 10-400 Hz). Then, the full-wave rectification was conducted. Based on the determined most appropriate signal-to-noise ratio, the low-pass filtered with a 6 Hz cut-off frequency was performed [36].

EMG signal was normalized by dividing the maximum EMG amplitude of maximal voluntary contraction (MVC) by the maximum root mean square amplitude to obtain the normalized signal $e_{i}(t)$. Muscle activation mirrors the degree of active force generated by the muscle. This study used a recursive model (second-order differential equation) to solve the muscle activation $a_{i}(t)$ by the obtained normalized signal $e_{i}(t)$ [37, 38]. First, the neural activation $u_{i}(t)$ was solved by $e_{i}(t)$:

$u_{i}\left( t \right)=\alpha e_{i}\left( t-d \right)-\beta_{1}u_{i}\left( t-1 \right)-\beta_{2}u_{i}\left( t-2 \right)$ (21)

Where the $t$ means the activation at time $t$, and the single neural activation $u_{i}(t)$ was related to the previous two activations $u_{i}\left( t-1 \right)$ and $u_{i}\left( t-2 \right)$. The $d=10 ms$ is the electromechanical delay, and $\alpha$, $\beta_{1}=C_{1}+C_{2}$, $\beta_{2}=C_{1}\times C_{2}$ are the coefficients defining the second-order dynamics. These parameters map $e_{i}(t)$ to $u_{i}\left( t \right)$, which is the key to forming a stable equation, and must satisfy the following conditions:

$\alpha=1+\beta_{1}+\beta_{2}$ (22)

The $C_{1}$, $C_{2}$ satisfy $\left| C_{1} \right|<1$, $\left| C_{2} \right|<1$. Then, the muscle activation $a_{i}\left( t \right)$ was solved by the non-linear model [37]:

$a_{i}\left( t \right)=\frac{e^{A_{i}u_{i}(t)}-1}{e^{A_{i}}-1}$ (23)

Where the $A_{i}$ is the nonlinear shape coefficient and represents the degree of nonlinearity of the neural activation $u_{i}(t)$ and muscle activation $a_{i}(t)$. The range of $A_{i}$ is allowed from -3 (highly exponential) to 0 (linear relationship), and the current study takes $A_{i}$ to be 1.5 [38].

All data were imported into MATLAB (Visual R2022a, MathWorks, United States) to expand into 101 data point curves (0%-100% landing phase) by self-written MATLAB scripts.

**3. Section C:** **Achilles Tendon and Plantar Fascia Model Creation and Property Setting**

To accurately assess loading variations in the foot Achilles tendon and plantar fascia during running, this study developed the subject-specific foot-ankle musculoskeletal model that accompanied by ligament-dense connective tissue. The foot-ankle musculoskeletal model for the current study included 36 ligaments, each ligament spanning at least two of the defined segments. The realization of these structures requires a geometric path, described by a set of anatomically fundamental points (origin, insertion and optionally passage points) [5]. In assessing the injury mechanism of ligaments, the conventional CLM model primarily characterizes ligaments as possessing linear force-length properties and regards them as SRTD [39, 40]. However, ankle ligament DCT is characterized by NLSTV strain, which affects their force-length characteristics [41]. Therefore, to more realistically revert and simulate the ALLI mechanics, we developed a CLM model (**Fig 2**) that reverts the ligament to a NLSTV mechanical mechanism (SRTD) generated by the DCT as a function of strain. Based on the MRI data, we calculated the path along the bundle in the resting state of the ligament DCT using the Euclidean distance formula [41, 42]. Detailed descriptions of ligament parameters and geometry paths are provided in the **Table S3**. By constructing the SCM, this study calculated the ligament loading force at different strains [43].

As presented in **Fig 2**, we developed a graphical user interface (GUI) for OpenSim with App Designer in MATLAB to evaluate the ligament material parameters. Meanwhile, ligament elongation and DCT forces were computed utilizing the muscle and force analysis tools in the OpenSim analysis toolbox (**Fig 2A**). In light of the short-term viscoelastic properties of ligament DCTs, the current study employs the sixth-order polynomial equation within the GUI to determine the material parameters [1, 41, 43]. The coefficient matrix $\bar{p}_{m,n}$ of this sixth-order polynomial equation is delineated as follows:

$$\bar{p}_{m,n}=\frac{\sum_{m=1}^{K} p_{m,n}}{L} \left( n=1, 2, \ldots m \right) (24)$$

The $K$ represents the sample size for this coefficient matrix equation, whereas $L$ denotes the number of coefficients. The coefficient matrix result is calculated from the coefficient term $p_{m,n}$ for each sample $n$. To simulate the intrinsic ligament model, we conducted a *C++* library (containing the $equation.h$ and $equation.cpp$ files) using the Win64 *C++* compiler, employing an adaptive product algorithm for numerical integration [1, 44]. The NNL class was created in Visual Studio (Microsoft Ltd. 2017), and the NLSR.dll and NLSR.lib folders were established in the OpenSim plugin to register the NLL class within the OpenSim GUI. This study inputs the material and mechanical parameters of the ligament DCT in the GUI based on the constructed SCM to define the properties of the DCT bundle [1] (**Fig 2B**). The ankle ligament model constructed in OpenSim and MATLAB is shown in **Fig 2C**. Finally, the nonlinear characteristic strains and internal loading forces of the DCT under different strains were calculated based on the analysis tools in OpenSim.

The ligament DCT length was defined as the resting length $L_{r}$ based on the state when the body was in the neutral position, and the extended length of the DCT when the body was moved during running was defined as $L_{e}$, and the ligament strain was calculated by the percentage of $L_{e}$ to $L_{r}$: $\varepsilon_{s}=\frac{L_{e}-L_{r}}{L_{r}}$. Among them, there is a "ligament-class" property in OpenSim, which means that there is a defect in that the extension length cannot be calculated during the dynamic simulation of SL [1, 10]. Therefore, in this study, the instantaneous strain rate (ISR) $\dot{\varepsilon}$ was calculated by differentiating the strain with respect to time [4]: $\dot{\varepsilon}=\frac{d\varepsilon_{s}}{dt}$.

Then, by assuming that the mean axial direction of ligament collagen fibers is parallel to the loading direction of the stress ($F_{a}$), the probability density function defining the fiber orientation under the orientation coefficient $m$ of the collagen fibers is $R\left( m \right)=\delta\left( m-F_{a} \right)$. Where $\delta$ is the Dirac delta function. Constructing computational functions for nominal axial stress based on strain and strain rate [1, 4, 43]：

$$\sigma_{af}=-p\lambda^{-1}+\sigma_{e}\left( \lambda\right)+\sigma_{v}\left( \lambda,\dot{\lambda} \right) (25)$$

The boundary conditions are set to no traction, that is, the pressure term $p$ is equal to 0 [1, 43]. The $\lambda$ represents the ratio of ligament extension length $L_{e}$ relative to resting length $L_{r}$: $\lambda=\frac{L_{e}}{L_{r}}$. Then, the $\sigma_{e}$ is the elastic stress generated in the collagen fibers of the ligament:

$$\left\{ \begin{aligned} \sigma_{e}\left( \lambda\right)=\int_{1}^{\lambda} R\left( \lambda_{s} \right)\bar{\sigma_{e}}(\frac{\lambda}{\lambda_{s}})d\lambda_{s} \\ \bar{\sigma_{e}}\left( \lambda\right)=K\ln\lambda\end{aligned} \right. (26)$$

Where the $\bar{\sigma_{e}}$ is the elastic stress generated in each collagen fiber of the ligament. $R\left( \lambda_{s} \right)$ represents the probability density function of the fiber activity density of the ligament in the stretched state ($\lambda_{s}$). $K$ is a constant, taken as 70 MPa, which is used to represent the collagen fiber’s elastic modulus [1, 41]. The $\sigma_{v}$ is the viscous stress generated in the collagen fibers of the ligament:

$$\left\{ \begin{aligned} \sigma_{v}\left( \lambda\right)=\int_{1}^{\lambda} R\left( \lambda_{s} \right)\bar{\sigma_{v}}(\frac{\lambda}{\lambda_{s}},\frac{\dot{\lambda}}{\lambda_{s}})d\lambda_{s} \\ \bar{\sigma_{v}}\left( \lambda_{r},\dot{\lambda_{r}} \right)=\eta\frac{D}{D_{t}}(ln \lambda_{r}) \end{aligned} \right. (27)$$

Where $\lambda_{r}$ is the stretch ratio of $\lambda$ and $\lambda_{s}$, the $\eta$ represents the absolute viscosity coefficient, taken as 20 MPa/S [1, 41]. The term $\frac{D}{D_{t}}$ represents the time derivative of matter. Therefore, combining ***Eqs. 25, 26, and 27***, the following equation can be obtained:

$$\sigma_{af}=\int_{1}^{\lambda} R\left( \lambda_{s} \right)\left\{ K\ln\frac{\lambda}{\lambda_{s}}+\eta\frac{D}{D_{t}}(\ln\frac{\lambda}{\lambda_{s}}) \right\}d\lambda_{s} (28)$$

Based on previous studies [4, 41, 43, 45], distribution functions were used to solve for the uncoiled stretch of collagen fibers to obtain the probability distribution of their unwinding $R\left( \lambda\right)$:

$$R\left( \lambda\right)=\left( \frac{ln\lambda-\gamma}{\beta} \right)\frac{\alpha}{\beta}\left( \frac{ln\lambda-\gamma}{\beta} \right)^{\alpha-1}exp\left( -\left( \frac{ln\lambda-\gamma}{\beta} \right)^{\alpha} \right) (29)$$

Where $\alpha, \beta>0$, are the shape, and scale with boundary conditions, respectively. The $\gamma$ is the location parameter, when it converges to zero based on the Weibull model [1, 43] $\lambda_{s}=e^{\varepsilon_{s}}$. So, the final calculation function of the nominal axial stresses developed based on the DCT is:

$$\sigma_{af}=\int_{0}^{\varepsilon} \alpha\beta^{-\alpha}\varepsilon_{S}^{\left( \alpha-1 \right)}e^{-\left( \frac{\varepsilon_{s}}{\beta} \right)^{\alpha}}\left[ K\left( \varepsilon-\varepsilon_{s} \right)+\eta\dot{\varepsilon} \right]d\varepsilon_{s} (30)$$

Where $\alpha=4.5$ and $\beta=0.3$ are the shape and scale factors of the probability distribution function, respectively, for the sequential straightening of collagen fibers under stress loading in the 2-parameter Weibull model [4, 41, 43].

Based on the mechanical and material properties of ligaments, the ligament fiber bundle model is defined as a nonlinear viscoelastic unit [1, 4, 26]. When the AT and PFS is in a resting or slack state, that is, the strain is equal to or less than zero, then the compressive stress within the ligament is zero [1]. When the DCT is in tension, the strain is greater than 0, the internal loading force is $F_{af}$:

$$F_{af}=\sigma_{af}A (31)$$

Where $A$ is the average physiologic cross-sectional area of the DCTs. The AT and PSF were modeled using the DCTs (**Fig 2C**). For the AT, the resting length is set to 20.4 cm, the cross-sectional area is set to 55.8 mm^2^, and the max isometric force is set to 2800 N [46, 47]. For the PFS, the PFS1, PFS2, PFS3, PFS4 and PFS5 resting length are set to 14.3 cm, 14.4 cm, 14.3 cm, 13.6 cm and 12.8 cm, the cross-sectional area is set to 69.2 mm^2^, and the max isometric force is set to 600 N [5, 48].

In addition, the lower limbs will dramatically change the acceleration and GRF of each segment during the stance phase, particularly in the early running stage, which will prevent the optimization process from being convergent. Therefore, during the whole modeling and simulation process, many attempts were performed to simulate the running using the computed muscle control (CMC) and reduce residuals algorithm (RRA) for each segment [1]. The strain of the AT and PFS varies with the foot-ankle angle under different running conditions. The AT and PFS strain can be seen as a function of foot-ankle kinematics according to the simulation results after CMC, which is the function of the muscle optimization process [2].

This work adjusts DCT material properties for passive fiber strain at the maximum isometric force, and if a passive DCT strain characteristic is specified as less than 1% in an individual model, additional adjustments are made to the models to ensure that the strain limit can be accurately controlled [2, 10]. For the contact area between ligament and bone (junction of ligament together with bones), the maximum strain was limited to 2.5-3.0% to ensure that the isometric force could start from the lowest value in the simulation [1, 2]. The length of bony osteoligamentous (tendons) and ligaments (muscles) will be examined during CMC based on the determined DCT strain when the presence of deformation of the entire muscle-tendon unit [10, 26].

**4. Section D: Lower Limb Tissue Mechanical Fatigue and Cumulative Injury**

Lower limb overuse injuries during running can be modeled as a mechanical fatigue phenomenon [49, 50]. Under the impact of CLs, the damage causes degradation of the material properties of human tissues, which will eventually lead to tissue failure [50, 51]. Based on lower extremity joint kinetics and tissue loads over a certain running distance or duration, the CLs were calculated [49]: $CL=n\int_{t_{i}}^{t_{f}} x_{s}d_{t}$. Where $n$ is the number of steps over a given running distance or duration, and $x_{s}$ is the peak load under the stance phase of that gait ($t_{i}$ is the start phase, $t_{f}$ is the end phase). Lower limb overuse injuries during running can be modeled as a mechanical fatigue phenomenon [49, 50]. Under the impact of CLs, the damage causes degradation of the material properties of human tissues, which will eventually lead to tissue failure [50, 51]. As presented in the **Fig 3**, overuse AT injuries, which are common in running-related injuries [49, 52], are characterized clinically by swelling and pain in the AT accompanied by inflammation, and mechanically by decreased AT Young's modulus and stiffness [53, 54]. Also, based on evidence from in vitro fatigue testing of human tendons, there is a highly nonlinear correlation between the degree of tissue fatigue and the magnitude of tendon strain [55, 56]. Prior to converting load shocks to empirically derived exponential powers (**Fig 3A and Fig 3B**), calculate CD. Explained by the inverse power law, the degree of loading had a greater effect on the cumulative tissue damage than the cumulative number of loads. Therefore, substituting the weighting factor $b$ in the equation can effectively explain the nonlinear relationship in the CD:

$$CD=\left[ n\int_{t_{i}}^{t_{f}} {(x_{s})}^{b}d_{t} \right]^{\frac{1}{b}} (32)$$

The corrected CD based on the weighting factors effectively reflects the CD behavior of ligaments, soft tissues, and bones [49, 55]. As seen in **Fig 3C**, for the AT, the weighting factor b represents the slope of the power function of the fatigue life calculated from experimental data with respect to the applied loading force/strain [49, 55]. For soft tissues, such as PFS ligaments, b is taken as 9.3; for tibial and patellar bones, etc., b is taken as 7 [50, 57]. With general engineering materials, the relationship between the peak stress magnitude $\varepsilon_{f}$, and the number of loads $N_{f}$, is interpreted based on the inverse power law:

$$N_{f}=A\varepsilon_{f}^{\frac{1}{b}} (33)$$

Where A is a constant of proportionality. When the human body receives load cycle impacts, biological tissue materials exhibit similar properties, and the logarithmic or exponential decay can be derived from the fatigue life of the body tissues [55, 58]. During running exercise, an increase in cyclic loading cycles also contributes to an increased risk of lower extremity musculoskeletal injuries in loading tasks. Mechanical fatigue CD modeling is effective in capturing the complex interrelationship between load magnitude and load cycles. Based on the Palmgren-Miner rule [59], the damage $D$ under a specific cyclic load is given by:

$$D=\sum_{i=1}^{m} \frac{n_{i}}{N_{fi}} (34)$$

Substituting $N_{f}$ into this equation:

$$D\propto\sum_{i=1}^{m} \frac{n_{i}}{A\varepsilon_{f}^{\frac{1}{b}}}=\sum_{i=1}^{m} n_{i}A\varepsilon_{f}^{b} (35)$$

Among them, for $m$ different load impact cases (running, jumping, landing, etc.), the damage risk induced by the $i-th$ load impact ${A\varepsilon_{f}}_{i}$ is the number of cycles at ${\varepsilon_{f}}_{i}$, $n_{i}$ divided by the number of destructive cycles ${A\varepsilon_{f}^{\frac{1}{b}}}_{i}$. However, the CD model constructed based on the Palmgren-Miner rule has inherent variability in tissue microstructure, which causes dispersed characteristics in fatigue behavior, thus affecting model accuracy [50]. Therefore, to solve the scattering problem that exists in material fatigue, we further bring in the Weibull analysis technique to the model [50, 55]. For a moving human body, the calculated fatigue strength of a tissue with a macrostructure that is subjected to cyclic impact until fatigue failure at a load-strain magnitude $\varepsilon_{f}$ is varied as:

$$P_{f}=1-e^{-\left( \frac{\varepsilon_{f}}{\varepsilon_{f},0} \right)^{mW}} (36)$$

Where $P_{f}$ is the fatigue damage probability of a given body tissue, $\varepsilon_{f}$ is the external strain, ${(\varepsilon}_{f},0)$ is the reference strain, and $mW$ is the Weibull modulus. The reference strain scale parameter is the applied strain magnitude when the fatigue damage probability is 63.2% ($P_{f}=1-e^{-1}$). The Weibull modulus explains the variability of the fatigue life data (**Fig 3C**), and the optimized estimation of distribution data based on normalized strain relations: the mean fatigue strength $\varepsilon_{mean}=0.1{(N_{s})}^{-0.063}$，$mW=6.7$，${(\varepsilon}_{f},0)=1.076\times\varepsilon_{f}$ (**Fig 3D** and **Fig 3E**) [50]. For running exercise, $\varepsilon_{mean}$ is a function of the number of gait stance phases at a given distance, and $N_{s}$ is the number of running steps per unit distance. Based on these parameters, the model explained almost 97% of the variance in experimentally measured fatigue intensity. However, the above equation $P_{f}$ fails to consider that the tendon is affected by both fatigue (cycle-related damage) and creep (time-related damage) [49, 60]. Therefore, the calibration factor was determined by vertically shifting the best-fit line in **Fig 3C**:

$$c=-0.010\left( \frac{1}{{2t}_{stance}} \right)+1.021 (37)$$

Where the $t_{stance}$ is the mean time of stance phase. The PF considering creep damage is obtained by substituting the correction factor into the above equation:

$$P_{f}=1-e^{-\left( \frac{\varepsilon_{f}}{1.076\times{(\varepsilon}_{f},c)} \right)^{6.7}} (38)$$

To summarize, besides calculating the impact load forces at the four common injury sites during running, we further calculated the CL, CD, and PF.

**5. Section E: Model Validation**

This work collected the foot-ankle fluoroscopic image data of the subjects in the running state by the high-speed dual fluoroscopic imaging system (DFIS) (Ti-WISH-Ⅱ, Ti-Motion ltd., Shanghai City, CN), and compared the calculated tibiotalar displacement results with the musculoskeletal modeling simulations results to verify the validity of foot-ankle model with DCTs (AT and PFS). **Fig 4** illustrates the procedure and outcomes of foot-ankle displacement calculation based on DFIS. By generating high voltage through a high voltage generator, the DFIS can accelerate the free electrons inside the X-ray tube towards the target surface to emit X-rays. Afterward, based on the principle that the X-rays emitted pass through different tissues and organs of the human body with different levels of attenuation, the X-rays that pass through the human body and carry enough information are projected onto a flat-panel detector. The computer processes the analog signals into digital signals through photoelectric conversion and analog-to-digital conversion, thereby providing reliable gray-scale medical image data for clinical diagnosis. The parameters setting of DFIS are as follows: source image distance 1350 mm, device voltage 60 kV, device current 500 mA, exposure time 3 seconds, laser wavelength 650 nm, dose range for the automatic exposure control 45 μGy, flat panel detector size 3072×3072 pixel, X-ray area 427×427 mm, nominal voltage of the ray tube 150 kV, laser power 3 mW, and sector angle 90°.

We constructed a 3-D model of the foot-ankle-knee integration according to the medical imaging data of the subject’s dominant limb (**Fig 4A**). First, the 3-D model generated in Mimics, utilizing previously obtained foot MRI/CT data was used as a standard framework for model correction and alignment. The segmentation of the two-dimensional image was performed using Mimics 21.0 (Materialise, Leuven, Belgium), while the creation and refinement of a three-dimensional model of the bone, ligaments, and bulk soft tissue was carried out using Geomagic Studio 2021 (Geomagic, Inc., Research Triangle Park, NC, United States). The SolidWorks 2017 software was utilized to convert the components into solids subsequent to their importing (SolidWorks Corporation, Waltham, MA, United States). The structure of cartilage was replicated through the construction of a solid material between the surfaces of two bones that are in contact with each other. The contacts of all three models were meshed and established utilizing Workbench 2021 software (ANSYS, Inc., Canonsburg, PA, USA). Hexahedral meshes were employed to decompose each solid. The mesh sizes for the bulk soft tissue, bone, cartilage, and ACL were adjusted to 2 mm, 2 mm, 0.5 mm, and 0.5 mm, respectively (**Fig 4B**). Furthermore, the process of refining at the local level was conducted with careful consideration of the geometric characteristics of the contact zone. The Workbench software facilitated the automated detection of component contacts. Possible contact pairings were generated using an algorithm that relies on surface proximity. The physical interaction between the surface of the foot bone and the cartilage was simulated through face-to-face contact. The coefficient of friction, defined as 0.04, was used to describe the sliding friction that occurs between the bones and cartilages [61, 62].

The musculoskeletal model was driven by kinematic and kinetic data collected during the running via VICON and force platform equipment to calculate the degree of displacement in all three directions (anterior-posterior, proximal-distal, medial-lateral). The two fluoroscopes were placed in planes orthogonal to each other by 90° to enable simultaneous capture of foot-ankle fluoroscopy image data from two orthogonal projections (anterolateral and anteromedial). A cubic calibration frame spatially calibrated the shooting area, and the XMAlab software (Version 2.1.0, Brown University, Prov, USA) calculated the relative positions of the X-ray tube and the flat panel detector in space [63]. Subsequently, the environment calibration file generated by XMAlab was imported into the 3-D modeling software Rhinoceros (Version 7.4, Robert McNeel Ltd., WA, USA) to create a virtual bi-orthogonal perspective system [63, 64]. Two virtual cameras were created to represent the x-ray sources of the two fluoroscopes, based on the placement of orthogonal fluoroscopic images to restore the position of the fluoroscopic enhancers (**Fig 4C**). After that, the real foot-ankle model was imported into the virtual space, and the bone position was adjusted by rotation and translation in Rhinoceros to align the projected contour of the knee model with the actual bone position on the X-ray fluoroscopic images [3]. In this, the reconstructed 3-D foot-ankle model was used as a standard framework for model correction and alignment. Finally, the tibia-fibula, talus, and calcaneus coordinate systems were established, and tibiotalar displacements were calculated using the coordinate system calculator plug-in in Rhinoceros [64]. As shown in **Fig 4D**, the tibiotalar displacement results obtained based on musculoskeletal simulations and DFIS calculations in the three directions are basically consistent, which demonstrates the feasibility of the foot-ankle model with DCTs constructed in the current study.

**6. Section F: Data-Driven Deep Learning Model Estimates for Lower Extremity Injury Risk Modeling**

To evaluate ligament loading and fatigue-related injury risk across four common running injury sites, we developed a multimodal deep learning framework that integrates wearable-sensor data with subject-specific musculoskeletal simulation outputs. Time-normalized acceleration waveforms collected from foot, tibial, and femoral IMUs (${101}_{data points}$) served as the primary inputs, while the predicted outputs included tissue forces for the plantar fascia, Achilles tendon, tibia, and patellofemoral joint. All input channels were normalized before training to minimize scale-related biases. The predicted forces were subsequently used to derive CL, CD, and PFF.

As illustrated in **Fig 1D**, the architecture integrates four core computational components—Sparrow Search Optimization (SSO), Convolutional Neural Network (CNN), Bidirectional Long Short-Term Memory (BiLSTM), and a Hybrid Attention Mechanism (HAM)—organized in a hierarchical configuration. In this architecture, the CNN extracts local spatial features from biomechanical signals, while the BiLSTM captures temporal dependencies reflecting fatigue progression. The HAM dynamically adjusts the weight of each feature channel, improving interpretability and sensitivity to fatigue-related variations. The SSO algorithm optimizes network parameters, enhancing convergence efficiency and predictive accuracy.

In the extended model, FPI scores and HVA were added as structural inputs, allowing the network to incorporate pronation-related variability and cumulative workload into the prediction of fatigue indices (**Fig 1D)**. For clarity, the original SSO–CNN–BiLSTM–HAM architecture is referred to as Model 1, whereas the enhanced version integrating FPI and HVA is defined as Model 2. Compared with Model 1, Model 2 enables the network to explicitly account for FPI and HVA–driven loading patterns, thereby improving the precision of CL, CD, and PFF estimation across all tissues. This structure-aware extension strengthens the model’s ability to capture individualized fatigue trajectories, particularly in runners with hallux valgus.

**4.1 Sparrow Search Optimization Model to Optimize Hyperparameters**

SSA is novel and has been proved to have the advantages of strong searching ability and fast convergence speed [65]. By using virtual sparrows to search for food, a population of $n$ sparrows is represented as follows:

$$S= \left[ \begin{matrix} \begin{matrix} s_{1}^{1} & s_{1}^{2} & \cdots\\ s_{2}^{1} & s_{2}^{2} & \ldots\\ \vdots& \ddots& \ddots\end{matrix} & \begin{matrix} s_{1}^{d} \\ s_{2}^{d} \\ \vdots\end{matrix} \\ \begin{matrix} s_{n}^{1} & s_{n}^{2} & \cdots\end{matrix} & s_{n}^{d} \end{matrix} \right] (39)$$

Where the $d$ is the dimension of the problem variable to be optimized, $n$ is the population of sparrows, and the fitness value $F_{s}$ is arranged as follows:

$$F_{s}=\left[ \begin{matrix} \begin{matrix} f(s_{1}^{1} & s_{1}^{2} & \cdots\\ f(s_{2}^{1} & s_{2}^{2} & \ldots\\ \vdots& \ddots& \ddots\end{matrix} & \begin{matrix} s_{1}^{d}) \\ s_{2}^{d}) \\ \vdots\end{matrix} \\ f(\begin{matrix} s_{n}^{1} & s_{n}^{2} & \cdots\end{matrix} & s_{n}^{d}) \end{matrix} \right] (40)$$

Where the $f$ is the fitness value of a single sparrow, and the finder with a better fitness value will preferentially obtain food during the search process. In addition, the finders are responsible for finding food for the entire sparrow population and providing foraging directions for all entrants, so the finder can obtain a larger foraging search range than the entrant. The position update speed of the finder in each iteration is as follows:

$$S_{i,j}^{t+1}=\left\{ \begin{aligned} S_{i,j}.\exp\left( -\frac{i}{\alpha.itermax} \right), if R_{2}<ST \\ S_{i,j}+Q.L, if R_{2}\geq ST \end{aligned} \right. (41)$$

Where the $t$ is the number of current iterations, $j=1, 2, 3, \ldots, d$, the $itermax$ is a constant representing the maximum number of iterations. The $S_{i,j}$ represents the position information of the $i$-th sparrow in the $j$-th dimension, and the $\alpha\in(0,1]$ is a random number. The $R_{2}(R_{2}\in\left[ 0,1 \right])$ represents the early warning value, and the $ST(ST\in\left[ 0.5, 1 \right])$ represents the safety value. $Q$ is a random number and follows a normal distribution. $L$ is a matrix with elements equal to 1 and dimensions of $1\times d$.

When $R_{2}<ST$ means that there are no predators around the foraging environment at this time, and the finder can conduct the search operation. If $R_{2}\geq ST$, it means that the predator has been spotted by sparrows and alerted other sparrows, and all sparrows need to quickly fly to other safe places to feed. For entrants, they will always pay attention to the finder, once the finder finds a better food, the entrant will immediately fight for the food. If the entrant loses the battle for food, its position is updated according to the following formula:

$$S_{i,j}^{t+1}=\left\{ \begin{aligned} Q.\exp\left( \frac{S_{worst}-S_{i，j}^{t}}{\alpha.itermax} \right), if i>n/2 \\ S_{P}^{t+1}+\left| S_{i,j}-S_{P}^{t+1} \right|.A^{+}.L, otherwise \end{aligned} \right. (42)$$

Where the $S_{p}$ is the current optimal position occupied by the finder, and the $S_{worst}$ is the current global worst position. $A$ represents a $1\times d$ matrix in which each element is randomly assigned 1 or -1, and $A^{+}=A^{T}{(AA^{T})}^{-1}$. When $i>n/2$, it indicates that the $i$-th entrant with a lower fitness value does not get food, and at this time, the entrant needs to fly to other places for foraging to replenish more energy.

At the time of modeling, the sparrow that is aware of the danger is assumed to be from 10% to 20% of the total population. The initial position of the sparrow is randomly generated in the population, which can be expressed as follows:

$$S_{i,j}^{t+1}=\left\{ \begin{aligned} S_{best}^{t}+\beta.\left| S_{i,j}^{t}-S_{best}^{t} \right|, if f_{i}>f_{g} \\ S_{i,j}^{t}+K.\left（ \frac{\left| S_{i,j}^{t}-S_{worst}^{t} \right|}{\left( f_{i}-f_{w} \right)+\varepsilon} \right）, if f_{i}=f_{g} \end{aligned} \right. (43)$$

Where the $S_{best}$ is the current global optimal position, indicating that sparrows in this position are the best position in the population and very safe. The $\beta$ is a step control parameter, which follows the normal distribution of random numbers with mean 0 and variance 1. The $K\in[-1,1]$ is a random number, and the $f_{i}$ is the fitness value of the current sparrow individual. The $f_{g}$ and $f_{w}$ are the current global best and worst fitness values, respectively. $\varepsilon$ is the constant that avoids zero in the denominator. When $f_{i}>f_{g}$, indicates that the sparrow is at the edge of the population and is vulnerable to predators, and $f_{i}=f_{g}$ indicates that sparrows are aware of the danger and need to approach other sparrows to reduce the risk of predation [26, 35, 65]. The $K$ represents the step control parameter, which is also the direction of sparrow movement.

**4.2 Convolutional Neural Network Model** **to Extract Local Correlation Features**

CNN is a feed-forward neural network, mainly inspired by natural biological visual cognitive mechanisms, which consists of the convolutional layer, pooling layer, and fully connected layer [66]. The current study processes the input signal by composing three successive convolutional and pooling layers, and then implements a mapping to the output target in a fully connected layer [66, 67]. Each convolutional layer contains multiple feature maps, each of which is a 'plane' of multiple neurons that extract a feature of the input through a convolutional filter. The formula for the one-dimensional convolutional operation is as follows:

$$y^{m}=\sum_{i=1}^{c^{m-1}} x_{i}^{m-1}*w_{i}^{m}+b_{i}^{m} (44)$$

Where the $y^{m}$ represents the output value of the local region of layer $m$, $c^{m-1}$ represents the $c-th$ channel of layer $m-1$, $x_{i}^{m-1}$is the output value of the $i-th$ channel of layer $m-1$ (input value of layer $m$), $*$ represents the 1-D convolution operation, $w_{i}^{m}$ represents the weight coefficient of the $i-th$ channel of layer $m$, $b_{i}^{m}$ represents the bias of the $i-th$ channel of layer $m$. After that, the data is pooled through the pooling layer, whose main role is to refine the feature vector obtained from the convolution operation by means of a downsampling operation, as a way to reduce the feature dimension and retain the most important features.

At this point, different features are generated within different channels. To get the most out of these features' information, we combine them through a fully connected layer to get a 1-D feature vector [34, 68, 69]. This can make the nonlinear representation of the features better. The output value of the fully connected layer $k$ is $y^{k}$:

$$y^{k}=\sum_{i=1}^{n} x_{i}^{k-1}w_{i}^{k}+b^{k} (45)$$

Where the $n$ represents the number of neurons in layer $k-1$, $x_{i}^{k-1}$ denotes the output value obtained by the $i-th$ neuron in layer $k-1$, $w_{i}^{k}$denotes the weight coefficient of the $i-th$ neuron in layer $k$, and $b^{k}$ denotes the bias of the $i-th$ neuron in layer $k$.

The Rectified Linear Unit (Relu) was used as the activation function ($f\left( x \right)=\max\left( 0,x \right), x\in(0,+\infty)$), the top of the fully connected layers activated using a SoftMax output, with the filter size and stride configuration of 1-4, and the number of filters in the convolutional layer was set to 32. Meanwhile, we applied the regularization technique Dropout to the output of the CNN to avoid model overfitting [70], where the neuron dropout probability was set to 0.5 [71]. In each training iteration, certain hidden layer units in the neural network are randomly dropped with a set drop probability. Dropped neurons are not involved in the computation during both forward and backward propagation. During the testing phase, all neurons are retained, and the output of each neuron is adjusted to remain consistent with the training phase. Dropout randomly drops neurons and forces the network to learn more robust features, thereby reducing the risk of overfitting. In addition, it can make the network more generalized and better adapted to the input of new samples [70].

**4.3 Bidirectional Long Short‑Term Memory Networks Model to Capture Feature Dependencies**

LSTM is a special structure of the recurrent neural network (RNN) proposed by Hochreater and Schmidhuber, which can learn long-term dependent information and successfully solve the gradient explosion and gradient disappearance problems of traditional RNN in backpropagation algorithm [72, 73]. For the LSTM detailed internal structure, which introduces four interacting layers: three $\sigma\left( z \right)=\frac{1}{1+e^{-z}}$, one $tanh\left( z \right)=\frac{e^{z}-e^{-z}}{e^{z}+e^{-z}}$. Meanwhile, the LSTM introduces the “Gate” structure to implement the filtering of information from the input neurons, finally to run through the horizontal line from $c_{t-1}$ to $c_{t}$ and to ensure selective updating of information. There are three gate structures: Forget Gate Layer (FGL), Input Gate Layer (IGL) and Output Gate Layer (OGL), which are mainly accomplished by a sigmoid ($\sigma\left( z \right)$) neural layer and a point-by-point multiplication operation [72].

For the FGL, it mainly determines whether the $s_{t-1}$ and $c_{t-1}$ states of the previous moment are retained in the $c_{t}$ of the current moment. Its input is the output $s_{t-1}$ at the previous moment and the input $x_{t}$ at the current moment, which is transformed by the sigmoid function to obtain the current moment internal output $f_{t}$ [72, 73]:

$$f_{t}=\sigma(W_{f}\left[ s_{t-1},x_{t} \right]+b_{f}) (46)$$

Where the $W_{f}$ represents the weight matrix of the FGL, $\left[ s_{t-1},x_{t} \right]$ represents the two-vector longitudinal join operation, and $b_{f}$ represents the bias term of the FGL.

The IGL determines how much of the input $x_{t}$, at the current moment, needs to be saved to the current cell state $c_{t}$. The implementation of the function consists of two layers: a sigmoid layer to decide which inputs are updated, and a $tanh$ layer to generate a vector as alternative update information. The outputs of these two layers are multiplied point by point so that the unit state $c_{t}$ is updated to calculate $i_{t}$ and $\tilde{c_{t}}$:

$$i_{t}=\sigma\left( W_{i}\left[ s_{t-1},x_{t} \right]+b_{i} \right); \tilde{c_{t}}=tanh\left( W_{c}\left[ s_{t-1},x_{t} \right]+b_{c} \right) (47)$$

Where the $W_{i}$ represents the weight matrix of the IGL, the $b_{i}$ represents the bias term of the IGL, $W_{c}$ represents the weight matrix of the unit state, the $b_{c}$ represents the bias term of the unit state. Then, the unit state $c_{t}$ can be calculated:

$$c_{t}=f_{t}\times c_{t-1}+i_{t}\times\tilde{c_{t}} (48)$$

Where the $\times$ represents the element-by-element multiplication.

The OGL mainly controls how much of the unit state $c_{t}$ can be output to the current output value $s_{t}$ of the LSTM. Specifically, this layer performs an elemental multiplication operation on the data after the unit state has been processed by the $tanh$ layer, and then the resulting $s_{t}$ is selectively output to the next moment and the external output. The calculation formula is as follows:

$$o_{t}=\sigma\left( W_{o}\left[ s_{t-1},x_{t} \right]+b_{o} \right); s_{t}=o_{t}\times tanh(c_{t}) (49)$$

In general, the training process has three main steps [26, 65]: 1) Calculated the output value of each neuron ($f_{t}, i_{t}, c_{t}, o_{t}, s_{t}$) by forward calculation; 2) Calculated the error term value of each neuron by backpropagation, which involves two directions: backpropagation according to the time series, that is, calculation of the value of the error term at the previous moment $t-1$ from the current moment $t$; back propagation according to the spatial structure, that is, calculation of the value of the error term from the current layer $l$ up one layer $l-1$; 3) Calculated the gradient value of each weight matrix and performed the update operation based on the two-directional error term values.

BiLSTM has two LSTM layers with the same number of neurons [74]. It combines the forward and backward information patterns of the time series data to give a more complete understanding of the sequence data. Forward LSTM deals with the order of the input sequences, while backward LSTM deals with the reverse order of the input sequences, and its expression is as follows:

$$\vec{s_{t}}=\sigma(w_{t}^{\vec{s}}x_{t}+w_{t-1}^{\vec{s}}\vec{s_{t-1}}+b^{\vec{s}}) (50)$$

$$s_{t}=\sigma(w_{t}^{s}x_{t}+w_{t-1}^{s}s_{t+1}+b^{s}) (51)$$

Where the $\vec{s_{t}}$ is the output of the hidden layer in the forward network at the current moment $t$, $s_{t}$ is the output of the hidden layer in the backward network at the current moment $t$, $\vec{s_{t-1}}$ is the output of the hidden layer in the forward network at the previous moment $t-1$, $s_{t+1}$ is the output of the hidden layer in the backward network at the later moment $t+1$, $x_{t}$ is the input of the current moment $t$, $w_{t}^{\vec{s}}$, $w_{t}^{s}$, $w_{t-1}^{\vec{s}}$, $w_{t-1}^{s}$are the corresponding weight coefficients in the network, $b^{\vec{s}}$, $b^{s}$are the corresponding biases in the network.

Finally, the outputs of the forward and backward LSTMs are linearly superimposed according to certain weights to obtain the final BiLSTM output:

$$S_{t}=w_{t}^{\vec{s}}\vec{s_{t}}+w_{t}^{s}s_{t}+b (52)$$

The setting of some hyperparameters in traditional LSTM for time series prediction is likely to cause problems such as underfitting, overfitting, slow convergence, or even failure to converge, thus affecting the model prediction performance. These hyperparameters include the number of hidden units, max epochs, initial learn rate, and L2 regularization [26]. For the number of hidden units, when their number in the neural network is too small it can lead to underfitting. When the quantity is excessively big, the finite information inside the training set is insufficient to adequately train all the neurons in the hidden layer, resulting in overfitting. Excessive neurons in the hidden layer, despite sufficient training data, will prolong training time and hinder the attainment of optimal outcomes. For the max epochs, a suitable number is the key to ensuring a better degree of convergence and improving the performance of the model. The initial learning rate is a hyperparameter that controls the learning rate of the neural network. A small initial learning rate will result in slow convergence of the model, while a large one will result in oscillation and failure of the network to converge. The L2 regularization can effectively prevent the overfitting of neural networks in backpropagation. Therefore, the current study used the prediction error of the BiLSTM network as a fitness function to find an optimal set of hyperparameters to minimize the prediction error through the SSO model, and ultimately to accurately predict the ankle ligament force during the whole movement phase [26, 65].

**4.4 Hybrid Attention Mechanisms to Optimize Prediction Models**

As shown in **Fig 1**, HAM can extract features in the input series that are more relevant and important to the output results [75, 76]. By focusing on important time steps and localized, detailed features in the time series, the model can better understand the patterns and trends in the time series. The current study developed a self-attention with scaled dot-product attention as the core layer of HAM, which is then combined with a multi-head attention mechanism on the extracted key features and weighted aggregation. The HAM model primarily utilizes the BiLSTM model to align the last cell state in the implicit state with the cell state of the input at the current step. After that, the correlation between the output state and the candidate intermediate states is calculated. In this process, the model extracts important and relevant information as much as possible and suppresses irrelevant and redundant information, to improve the performance of the prediction models [68, 77].

Attention is a mechanism used to enhance the performance outcomes of BiLSTM-based encoder and decoder models [75]. Attention mechanisms have been widely used in research fields such as machine translation, image capture, and engineering prediction due to their ability to enhance model distinction discrimination [76]. The attention method enables the model to allocate varying weights to distinct segments of the input data, thereby extracting more pertinent information for the resultant judgment, thus making the prediction result of the model more accurate [78]. It is also believed that this process does not increase the load of model computation and storage [78]. The attention mechanism can be divided mainly into soft attention and hard attention. Soft attention is to keep all the components for weighting, and hard attention is to select some of the components with some strategy [76]. Soft attention is parameterization, which enable directly derived and embedded into the model for direct training. Also, the gradient in soft attention can be back-propagated throughout the model via the attention mechanism module, making it more preferred in most research and applications [76]. Therefore, the current study determined to use the soft attention module to build HAM.

The core of soft attention is the sequence-to-sequence learning model, that is, the problem of mapping an input$X$ to an output $Y$. The encoder encodes the input sequence $x_{1}, x_{2}, \ldots, x_{n}$ into a fixed-length hidden vector. Among them, this hidden vector serves two roles: (1) as an initial vector to initialize the decoder model to predict $y_{1}$; (2) as a context vector to guide the output of $y$ at each step in the $y$ sequence. Therefore, the decoder decodes the output of that moment based mainly on the hidden vector and the output of the previous step until the end of the run [78]. The self-attention allows the model to consider both past and future information when processing each time step, helping to better capture long-range dependencies in sequences [75, 76]. However, the following shortcomings exist in traditional soft attention: (1) The sequence-to-sequence model lacks differentiation of the input sequence $X$; (2) the dependency between the features at the input end and the features at the output end is often ignored. Therefore, this study further introduced self-attention with scaled dot-product attention as the core layer of HAM. For scaled dot-product attention, it is mainly scaled by a scaling factor when calculating the weights, which can effectively avoid the problem of gradient explosion [75]. By adaptively giving weights to different time steps in a learning manner, it is well suited for longer force-time curve data prediction in this study.

The attention function is defined as the mapping of a query and a collection of key-value pairs to an output, with the query, keys, values, and output all represented as vectors [75]. A compatibility function between the query and the associated key dictates the weights allocated to each value during the computation of the output as a weighted sum of the values. Among them, the query and key of the feature dimension $d_{k}$ of the input data, and the learned value comprises the model inputs. By calculating the dot products of the query with each key, and dividing by $\sqrt{d_{k}}$, the final weight values are obtained based on the softmax function. The attention function for each set of queries is computed synchronously and packed into matrix $Q$. The keys and values are also packed together into matrices $K$ and $V$. The output matrix is given below:

$$Attention\left( Q,K,V \right)=softmax\left( \frac{QK^{T}}{\sqrt{d_{k}}} \right)V (53)$$

The $q$ and $k (0\pm1)$ are independent random variables. The dot product between them is: $q\cdot k=\sum_{i=1}^{d_{k}} q_{i}\cdot k_{i}$. To capture the different relationships and features in the input sequences more comprehensively, we further introduced a multi-head attention mechanism to enhance and improve the generalization of the model [75, 76, 78]. The model can examine information from various representation subspaces at distinct locations. This has the same total computational cost as full-dimensional single-head attention. For the input time series data, assuming its sequence length is $i$, there are $i$ nodes $x_{1}, x_{2},\ldots, x_{i}$. Then, through the input embedding layer, each discrete input symbol is converted into a continuous vector and mapped to higher dimensions by the corresponding feature number $H (h_{1}, h_{2}, \ldots,h_{i})$ of the input data. These embedded vectors are used as inputs to the encoder for capturing timing information in the input data. The query $Q$, key $K$, and $V$ (information values obtained through learning) are based on the result of the dot product of the transformation matrices $W_{q}$, $W_{k}$, and $W_{v}$ obtained from the training share with the $H$ vector, respectively, to obtain $q^{i}$, $k^{i}$, and $v^{i}$:

$$\left\{ \begin{aligned} q^{i}=W_{q}\cdot h^{i} \\ k^{i}=W_{k}\cdot h^{i} \\ v^{i}=W_{v}\cdot h^{i} \end{aligned} \right. (54)$$

The $q$ and $k$ will be matched to each other to compute the correlation between them, and then based on softmax the attention weights $a_{q^{i}}$:

$$a_{q^{i}}=\frac{exp(q^{i}\cdot k^{i})}{\sum_{i=1}^{n} exp(q^{i}\cdot k^{i})} (55)$$

The strength of the association is specified by the magnitude of the weight. Subsequently, all value vectors are weighted and aggregated according to the calculated attention weights to produce the context vector: $e_{q^{i}}=\sum_{i=1}^{n} a_{q^{i}}$. For each ${head}_{h}$ corresponding to $Q_{h}$, $K_{h}$, $V_{h}$ parameters, the corresponding weight results are calculated based on the above process:

$${head}_{h}=Attention(Q_{h}W_{i}^{Q},K_{h}W_{i}^{K},V_{h}W_{i}^{V}) (56)$$

The results obtained under each ${head}_{h}$ are concatenated to get the final weight result:

$$MultiHead\left( Q,K,V \right)=Concat({head}_{1},{head}_{2},\ldots,{head}_{h})W (57)$$

Within this, the context vector of the encoder output is used to initialize the decoder part (output embedding of the encoder), and it is used as the input to the decoder (input embedding of the decoder) along with the output of the predicted previous time step. The self-attention layer is contained in the encoder, whose corresponding $Q$, $K$, and $V$ all come from the previous layer’s output. Each encoder position can focus on all positions in the encoder upper layer. Each decoder position in the self-attention layer can focus on decoder all positions. In the decoder stage, the continuous output generated by the model (predicted tissue impact and loading force values for future time steps) is converted into discrete symbols to match the actual target values through the output embedding layer.

To evaluate the necessity and effectiveness of the proposed architecture, we first conducted systematic comparisons with several baseline models, including standalone CNN, BiLSTM, and their simplified hybrid variants. These baseline models were designed to represent commonly used structures for handling spatial or temporal features in biomechanical time-series data. Performance was evaluated using RMSE and NRMSE to ensure both absolute and normalized error assessment.

Building upon these baseline configurations, the proposed SSO–CNN–BiLSTM–HAM framework was designed to progressively integrate spatial feature extraction, temporal sequence modeling, and adaptive feature weighting. Specifically, the CNN module was employed to capture local spatial patterns in the input signals and to learn feature-level importance weights. These weights reflect the relative contribution of different biomechanical variables. The extracted features were then passed to the BiLSTM module, which models bidirectional temporal dependencies across the gait cycle. Compared to unidirectional recurrent structures, BiLSTM enables a more comprehensive representation of time-dependent biomechanical dynamics. The outputs of the BiLSTM were further combined with the CNN-derived feature weights via a dot-product operation, allowing interaction between spatial importance and temporal evolution. Subsequently, a hybrid attention mechanism (HAM) was introduced to adaptively reweight the fused features, enhancing the model’s ability to focus on critical gait phases and key fatigue-related variables. This attention-based refinement improves feature discrimination under complex and noisy biomechanical conditions. Finally, the integrated features were processed through a linear layer and a Sigmoid activation function to introduce nonlinearity, followed by a Softmax function for output normalization and prediction.

The comparative results demonstrated that the proposed model consistently outperformed all baseline models, achieving lower RMSE and NRMSE values. This indicates that the hierarchical integration of spatial and temporal features, combined with attention-based weighting, provides a measurable advantage in predicting fatigue-related biomechanical indicators. These findings support the rationale for adopting the proposed architecture, particularly under limited sample conditions where efficient feature utilization is critical **(Figs S6–S7)**.

**7. SI References**

[1] D. Xu, H. Zhou, W. Quan, X. Ma, T.-E. Chon, J. Fernandez, F. Gusztav, A. Kovács, J. S. Baker, and Y. Gu, “New insights optimize landing strategies to reduce lower limb injury risk,” *Cyborg and Bionic Systems,* vol. 5, pp. 0126, 2024.

[2] D. Xu, H. Zhou, M. Wang, X. Ma, F. Gusztav, T.-E. Chon, J. Fernandez, J. S. Baker, and Y. Gu, “Contribution of ankle motion pattern during landing to reduce the knee-related injury risk,” *Computers in Biology and Medicine,* vol. 180, pp. 108965, 2024.

[3] R. J. De Asla, M. Kozánek, L. Wan, H. E. Rubash, and G. Li, “Function of anterior talofibular and calcaneofibular ligaments during in-vivo motion of the ankle joint complex,” *Journal of Orthopaedic Surgery and Research,* vol. 4, pp. 1-6, 2009.

[4] A. Sikidar, and D. Kalyanasundaram, “An open-source OpenSim® ankle-foot musculoskeletal model for assessment of strains and forces in dense connective tissues,” *Computer Methods and Programs in Biomedicine,* vol. 224, pp. 106994, 2022.

[5] T. M. Malaquias, C. Silveira, W. Aerts, F. De Groote, G. Dereymaeker, J. Vander Sloten, and I. Jonkers, “Extended foot-ankle musculoskeletal models for application in movement analysis,” *Computer methods in biomechanics and biomedical engineering,* vol. 20, no. 2, pp. 153-159, 2017.

[6] S. R. Ward, and R. L. Lieber, “Density and hydration of fresh and fixed human skeletal muscle,” *Journal of biomechanics,* vol. 38, no. 11, pp. 2317-2320, 2005.

[7] M. Farvid, T. Ng, D. Chan, P. Barrett, and G. Watts, “Association of adiponectin and resistin with adipose tissue compartments, insulin resistance and dyslipidaemia,” *Diabetes, obesity and metabolism,* vol. 7, no. 4, pp. 406-413, 2005.

[8] F. Tonon, “Explicit exact formulas for the 3-D tetrahedron inertia tensor in terms of its vertex coordinates,” *Journal of Mathematics and Statistics,* vol. 1, no. 1, pp. 8-11, 2005.

[9] C. R. Allen, E. K. Wong, G. A. Livesay, M. Sakane, F. H. Fu, and S. L. Y. Woo, “Importance of the medial meniscus in the anterior cruciate ligament‐deficient knee,” *Journal of Orthopaedic Research,* vol. 18, no. 1, pp. 109-115, 2000.

[10] S. L. Delp, F. C. Anderson, A. S. Arnold, P. Loan, A. Habib, C. T. John, E. Guendelman, and D. G. Thelen, “OpenSim: open-source software to create and analyze dynamic simulations of movement,” *IEEE transactions on biomedical engineering,* vol. 54, no. 11, pp. 1940-1950, 2007.

[11] G. Valente, G. Crimi, N. Vanella, E. Schileo, and F. Taddei, “nmsBuilder: Freeware to create subject-specific musculoskeletal models for OpenSim,” *Computer methods and programs in biomedicine,* vol. 152, pp. 85-92, 2017.

[12] R. Dumas, R. Aissaoui, D. Mitton, W. Skalli, and J. A. de Guise, “Personalized body segment parameters from biplanar low-dose radiography,” *IEEE Transactions on Biomedical engineering,* vol. 52, no. 10, pp. 1756-1763, 2005.

[13] G. Valente, L. Pitto, D. Testi, A. Seth, S. L. Delp, R. Stagni, M. Viceconti, and F. Taddei, “Are subject-specific musculoskeletal models robust to the uncertainties in parameter identification?,” *PloS one,* vol. 9, no. 11, pp. e112625, 2014.

[14] G. Wu, S. Siegler, P. Allard, C. Kirtley, A. Leardini, D. Rosenbaum, M. Whittle, D. D D’Lima, L. Cristofolini, and H. Witte, “ISB recommendation on definitions of joint coordinate system of various joints for the reporting of human joint motion—part I: ankle, hip, and spine,” *Journal of biomechanics,* vol. 35, no. 4, pp. 543-548, 2002.

[15] L. Scheys, A. Van Campenhout, A. Spaepen, P. Suetens, and I. Jonkers, “Personalized MR-based musculoskeletal models compared to rescaled generic models in the presence of increased femoral anteversion: effect on hip moment arm lengths,” *Gait & posture,* vol. 28, no. 3, pp. 358-365, 2008.

[16] H. Boey, S. van Rossom, S. Verfaillie, J. Vander Sloten, and I. Jonkers, “Maximal lateral ligament strain and loading during functional activities: Model-based insights for ankle sprain prevention and rehabilitation,” *Clinical Biomechanics,* vol. 94, pp. 105623, 2022.

[17] T. A. Correa, and M. G. Pandy, “A mass–length scaling law for modeling muscle strength in the lower limb,” *Journal of biomechanics,* vol. 44, no. 16, pp. 2782-2789, 2011.

[18] R. E. Isman, V. T. Inman, and P. Poor, “Anthropometric studies of the human foot and ankle,” *Bull Prosthet Res,* vol. 11, no. 10, pp. 97-129, 1969.

[19] M. Zhang, T. C. Davies, Y. Zhang, and S. Q. Xie, “A real-time computational model for estimating kinematics of ankle ligaments,” *Computer Methods in Biomechanics and Biomedical Engineering,* vol. 19, no. 8, pp. 835-844, 2016.

[20] G. S. Lewis, K. A. Kirby, and S. J. Piazza, “Determination of subtalar joint axis location by restriction of talocrural joint motion,” *Gait & posture,* vol. 25, no. 1, pp. 63-69, 2007.

[21] C. L. Brockett, and G. J. Chapman, “Biomechanics of the ankle,” *Orthopaedics and trauma,* vol. 30, no. 3, pp. 232-238, 2016.

[22] S. Weindel, R. Schmidt, S. Rammelt, L. Claes, A. v. Campe, and S. Rein, “Subtalar instability: a biomechanical cadaver study,” *Archives of orthopaedic and trauma surgery,* vol. 130, pp. 313-319, 2010.

[23] T.-Y. Chen, T. Kawakami, N. Ogihara, and K. Hosoda, “Free moment induced by oblique transverse tarsal joint: investigation by constructive approach,” *Royal Society Open Science,* vol. 8, no. 4, pp. 201947, 2021.

[24] V. Klema, and A. Laub, “The singular value decomposition: Its computation and some applications,” *IEEE Transactions on automatic control,* vol. 25, no. 2, pp. 164-176, 1980.

[25] P. J. Besl, and N. D. McKay, "Method for registration of 3-D shapes." pp. 586-606.

[26] D. Xu, H. Zhou, W. Quan, F. Gusztav, M. Wang, J. S. Baker, and Y. Gu, “Accurately and effectively predict the ACL force: Utilizing biomechanical landing pattern before and after-fatigue,” *Computer Methods and Programs in Biomedicine,* vol. 241, pp. 107761, 2023.

[27] D. Xu, H. Zhou, W. Quan, F. Gusztav, J. S. Baker, and Y. Gu, “Adaptive neuro-fuzzy inference system model driven by the non-negative matrix factorization-extracted muscle synergy patterns to estimate lower limb joint movements,” *Computer Methods and Programs in Biomedicine,* vol. 242, pp. 107848, 2023.

[28] F. De Groote, T. De Laet, I. Jonkers, and J. De Schutter, “Kalman smoothing improves the estimation of joint kinematics and kinetics in marker-based human gait analysis,” *Journal of biomechanics,* vol. 41, no. 16, pp. 3390-3398, 2008.

[29] R. E. Kalman, “A new approach to linear filtering and prediction problems,” 1960.

[30] Y. Bar-Shalom, X. R. Li, and T. Kirubarajan, *Estimation with applications to tracking and navigation: theory algorithms and software*: John Wiley & Sons, 2004.

[31] H. E. Rauch, F. Tung, and C. T. Striebel, “Maximum likelihood estimates of linear dynamic systems,” *AIAA journal,* vol. 3, no. 8, pp. 1445-1450, 1965.

[32] A. C. P. da Silveira, “Extended Biomechanical Model of the Ankle-Foot Complex: Incorporation of Muscles and Ligaments,” Universidade de Coimbra (Portugal), 2015.

[33] A. C. Redmond, J. Crosbie, and R. A. Ouvrier, “Development and validation of a novel rating system for scoring standing foot posture: the Foot Posture Index,” *Clinical biomechanics,* vol. 21, no. 1, pp. 89-98, 2006.

[34] D. Xu, W. Quan, H. Zhou, D. Sun, J. S. Baker, and Y. Gu, “Explaining the differences of gait patterns between high and low-mileage runners with machine learning,” *Scientific reports,* vol. 12, no. 1, pp. 2981, 2022.

[35] D. Xu, H. Zhou, W. Quan, X. Jiang, M. Liang, S. Li, U. C. Ugbolue, J. S. Baker, F. Gusztav, and X. Ma, “A new method proposed for realizing human gait pattern recognition: Inspirations for the application of sports and clinical gait analysis,” *Gait & Posture,* vol. 107, pp. 293-305, 2024.

[36] H. Zhou, D. Xu, W. Quan, U. C. Ugbolue, N. F. Sculthorpe, J. S. Baker, and Y. Gu, “A foot joint and muscle force assessment of the running stance phase whilst wearing normal shoes and bionic shoes,” *Acta Bioeng. Biomech,* vol. 24, pp. 191-202, 2022.

[37] D. G. Lloyd, and T. F. Besier, “An EMG-driven musculoskeletal model to estimate muscle forces and knee joint moments in vivo,” *Journal of biomechanics,* vol. 36, no. 6, pp. 765-776, 2003.

[38] T. S. Buchanan, D. G. Lloyd, K. Manal, and T. F. Besier, “Neuromusculoskeletal modeling: estimation of muscle forces and joint moments and movements from measurements of neural command,” *Journal of applied biomechanics,* vol. 20, no. 4, pp. 367-395, 2004.

[39] H. Xu, D. Bloswick, and A. Merryweather, “An improved OpenSim gait model with multiple degrees of freedom knee joint and knee ligaments,” *Computer methods in biomechanics and biomedical engineering,* vol. 18, no. 11, pp. 1217-1224, 2015.

[40] A. Sikidar, M. Marieswaran, and D. Kalyanasundaram, “Estimation of forces on anterior cruciate ligament in dynamic activities,” *Biomechanics and Modeling in Mechanobiology,* vol. 20, pp. 1533-1546, 2021.

[41] A. Sikidar, and D. Kalyanasundaram, “An open-source plugin for OpenSim® to model the non-linear behaviour of dense connective tissues of the human knee at variable strain rates,” *Computers in Biology and Medicine,* vol. 110, pp. 186-195, 2019.

[42] S. Standring, H. Ellis, J. Healy, D. Johnson, A. Williams, P. Collins, and C. Wigley, “Gray's anatomy: the anatomical basis of clinical practice,” *American journal of neuroradiology,* vol. 26, no. 10, pp. 2703, 2005.

[43] R. De Vita, and W. S. Slaughter, “A structural constitutive model for the strain rate-dependent behavior of anterior cruciate ligaments,” *International Journal of Solids and Structures,* vol. 43, no. 6, pp. 1561-1570, 2006.

[44] L. F. Shampine, “Vectorized adaptive quadrature in MATLAB,” *Journal of Computational and Applied Mathematics,* vol. 211, no. 2, pp. 131-140, 2008.

[45] R. Sopakayang, and R. De Vita, “A mathematical model for creep, relaxation and strain stiffening in parallel-fibered collagenous tissues,” *Medical engineering & physics,* vol. 33, no. 9, pp. 1056-1063, 2011.

[46] L. Li, K. Wu, L. Deng, C. Liu, and W. Fu, “The Effects of Habitual Foot Strike Patterns on the Morphology and Mechanical Function of the Medial Gastrocnemius–Achilles Tendon Unit,” *Bioengineering,* vol. 10, no. 2, pp. 264, 2023.

[47] S. M. Morrison, T. J. Dick, and J. M. Wakeling, “Structural and mechanical properties of the human Achilles tendon: Sex and strength effects,” *Journal of Biomechanics,* vol. 48, no. 12, pp. 3530-3533, 2015.

[48] A. Bisi-Balogun, and M. Rector, “Clinical utility of ultrasound measurements of plantar fascia width and cross-sectional area: a novel technique,” *Journal of the American Podiatric Medical Association,* vol. 107, no. 5, pp. 375-381, 2017.

[49] C. R. Firminger, M. J. Asmussen, S. Cigoja, J. R. Fletcher, B. M. Nigg, and W. B. Edwards, “Cumulative metrics of tendon load and damage vary discordantly with running speed,” *Medicine & Science in Sports & Exercise,* vol. 52, no. 7, pp. 1549-1556, 2020.

[50] W. B. Edwards, “Modeling overuse injuries in sport as a mechanical fatigue phenomenon,” *Exercise and sport sciences reviews,* vol. 46, no. 4, pp. 224-231, 2018.

[51] S. Gallagher, and M. C. Schall, "Musculoskeletal disorders as a fatigue failure process: evidence, implications and research needs," *New Paradigms in Ergonomics*, pp. 105-119: Routledge, 2020.

[52] I. Janssen, H. van der Worp, S. Hensing, and J. Zwerver, “Investigating Achilles and patellar tendinopathy prevalence in elite athletics,” *Research in Sports Medicine,* vol. 26, no. 1, pp. 1-12, 2018.

[53] N. Maffulli, “Overuse tendon conditions: time to change a confusing terminology,” *Arthroscopy: The Journal of Arthroscopic & Related Surgery,* vol. 14, no. 8, pp. 840-843, 1998.

[54] S. Arya, and K. Kulig, “Tendinopathy alters mechanical and material properties of the Achilles tendon,” *Journal of applied physiology,* vol. 108, no. 3, pp. 670-675, 2010.

[55] T. A. Wren, D. P. Lindsey, G. S. Beaupré, and D. R. Carter, “Effects of creep and cyclic loading on the mechanical properties and failure of human Achilles tendons,” *Annals of biomedical engineering,* vol. 31, pp. 710-717, 2003.

[56] H. Schechtman, and D. Bader, “In vitro fatigue of human tendons,” *Journal of biomechanics,* vol. 30, no. 8, pp. 829-835, 1997.

[57] B. Van Hooren, L. van Rengs, and K. Meijer, “Per‐step and cumulative load at three common running injury locations: The effect of speed, surface gradient, and cadence,” *Scandinavian Journal of Medicine & Science in Sports,* vol. 34, no. 2, pp. e14570, 2024.

[58] D. Carter, and W. Caler, “A cumulative damage model for bone fracture,” *Journal of Orthopaedic Research,* vol. 3, no. 1, pp. 84-90, 1985.

[59] N.-Z. Chen, G. Wang, and C. G. Soares, “Palmgren–Miner’s rule and fracture mechanics-based inspection planning,” *Engineering Fracture Mechanics,* vol. 78, no. 18, pp. 3166-3182, 2011.

[60] X. T. Wang, R. F. Ker, and R. M. Alexander, “Fatigue rupture of wallaby tail tendons,” *Journal of experimental biology,* vol. 198, no. 3, pp. 847-852, 1995.

[61] A. Godest, M. Beaugonin, E. Haug, M. Taylor, and P. Gregson, “Simulation of a knee joint replacement during a gait cycle using explicit finite element analysis,” *Journal of biomechanics,* vol. 35, no. 2, pp. 267-275, 2002.

[62] J. P. Halloran, A. J. Petrella, and P. J. Rullkoetter, “Explicit finite element modeling of total knee replacement mechanics,” *Journal of biomechanics,* vol. 38, no. 2, pp. 323-331, 2005.

[63] B. J. Knörlein, D. B. Baier, S. M. Gatesy, J. Laurence-Chasen, and E. L. Brainerd, “Validation of XMALab software for marker-based XROMM,” *Journal of Experimental Biology,* vol. 219, no. 23, pp. 3701-3711, 2016.

[64] G. Li, S. K. Van de Velde, and J. T. Bingham, “Validation of a non-invasive fluoroscopic imaging technique for the measurement of dynamic knee joint motion,” *Journal of biomechanics,* vol. 41, no. 7, pp. 1616-1622, 2008.

[65] J. Xue, and B. Shen, “A novel swarm intelligence optimization approach: sparrow search algorithm,” *Systems science & control engineering,* vol. 8, no. 1, pp. 22-34, 2020.

[66] Y. LeCun, and Y. Bengio, “Convolutional networks for images, speech, and time series,” *The handbook of brain theory and neural networks,* vol. 3361, no. 10, pp. 1995, 1995.

[67] Y. LeCun, L. Bottou, Y. Bengio, and P. Haffner, “Gradient-based learning applied to document recognition,” *Proceedings of the IEEE,* vol. 86, no. 11, pp. 2278-2324, 1998.

[68] T. Ma, G. Xiang, Y. Shi, and Y. Liu, “Horizontal in situ stresses prediction using a CNN-BiLSTM-attention hybrid neural network,” *Geomechanics and Geophysics for Geo-Energy and Geo-Resources,* vol. 8, no. 5, pp. 152, 2022.

[69] D. Xu, H. Zhou, W. Quan, U. C. Ugbolue, F. Gusztav, and Y. Gu, “A new method applied for explaining the landing patterns: Interpretability analysis of machine learning,” *Heliyon,* vol. 10, no. 4, 2024.

[70] H. Wu, and X. Gu, “Towards dropout training for convolutional neural networks,” *Neural Networks,* vol. 71, pp. 1-10, 2015.

[71] S. Wager, S. Wang, and P. S. Liang, “Dropout training as adaptive regularization,” *Advances in neural information processing systems,* vol. 26, 2013.

[72] S. Hochreiter, “Long Short-term Memory,” *Neural Computation MIT-Press*, 1997.

[73] F. A. Gers, J. Schmidhuber, and F. Cummins, “Learning to forget: Continual prediction with LSTM,” *Neural computation,* vol. 12, no. 10, pp. 2451-2471, 2000.

[74] S. Siami-Namini, N. Tavakoli, and A. S. Namin, "The performance of LSTM and BiLSTM in forecasting time series." pp. 3285-3292.

[75] A. Vaswani, “Attention is all you need,” *Advances in Neural Information Processing Systems*, 2017.

[76] Z. Niu, G. Zhong, and H. Yu, “A review on the attention mechanism of deep learning,” *Neurocomputing,* vol. 452, pp. 48-62, 2021.

[77] L. Shan, Y. Liu, M. Tang, M. Yang, and X. Bai, “CNN-BiLSTM hybrid neural networks with attention mechanism for well log prediction,” *Journal of Petroleum Science and Engineering,* vol. 205, pp. 108838, 2021.

[78] I. Sutskever, “Sequence to Sequence Learning with Neural Networks,” *arXiv preprint arXiv:1409.3215*, 2014.
