# Supplementary material for: Structure-aware fatigue modeling in foot deformities: A digital health framework for tissue-specific running injury risk prediction using multi-modal data
Source: PLOS Digit Health. 2026 Jul 10;5(7):e0001537. doi: 10.1371/journal.pdig.0001537 (PMC13354006; doi:10.1371/journal.pdig.0001537)
Supplement: Attachment — Submitted filename: PDIG-D-25-01162_reviewer_JULIAN_BORGES.pdf [file pdig.0001537.s004.pdf]

Structure Aware Fatigue Modeling in Foot Deformities A Digital Health Framework for Tissue Specific Risk Prediction Using Wearable Data

PDIG-D-25-01162_reviewer_JULIAN_BORGES

Summary

This manuscript presents a structure aware digital health framework linking hallux valgus related morphology and foot posture to tissue specific mechanical fatigue during running. The study integrates subject specific musculoskeletal simulation incorporating dense connective tissue mechanics with probabilistic fatigue modeling to derive cumulative load, cumulative damage, and probability of fatigue failure across four injury relevant regions. A deep learning model is then trained to predict simulated tissue forces and derived fatigue indices from wearable inertial signals, with further performance gains reported when hallux valgus angle and Foot Posture Index are incorporated as structural inputs.

The work is conceptually strong and aligned with the aims of digital health and biomechanics, particularly in its attempt to translate laboratory grade mechanistic understanding into a wearable enabled prediction framework. However, substantive concerns related to validation rigor, model complexity, reproducibility, and inferential framing limit confidence in generalizable predictive performance and real world applicability.

Major Issues

1. Validation rigor and control of information leakage

The manuscript does not clearly specify whether model training, validation, and testing were conducted using strict participant level separation. Given the use of repeated trials and multichannel time series data, any non participant level splitting could introduce information leakage and inflate performance estimates. The reported performance metrics necessitate explicit confirmation that leakage was prevented.

Recommendation:

- Clearly state the unit of data splitting and ensure all reported results are based on strict participant level separation.
- If cross validation is used, implement participant stratified folds and report variability across folds.
- Separate hyperparameter tuning from final performance estimation using a nested validation approach or a locked test set.
- Provide a sensitivity analysis demonstrating the impact of different splitting strategies on performance.

---

2. Model complexity relative to sample size

The proposed architecture combines multiple high capacity components and an optimization routine, yet the cohort size is limited. This raises substantial concern regarding overfitting and model stability. Performance is reported without uncertainty measures, further limiting interpretability.

Recommendation:

- Report confidence intervals or variability estimates for all predictive metrics.
- Provide learning curves or performance as a function of training size to assess data sufficiency.
- Report the number of trainable parameters and describe regularization strategies.
- Include an ablation study evaluating the incremental contribution of major architectural components and structural inputs.

---

3. Absence of baseline model comparisons

The manuscript does not demonstrate that the proposed model materially outperforms simpler alternatives. Without appropriate baselines, the added complexity of the model cannot be adequately justified.

Recommendation:

- Compare predictive performance against simpler baselines, including linear or penalized regression, tree based methods, and compact neural models.
- Evaluate all models using identical participant level validation procedures.
- Discuss tradeoffs between performance gains, complexity, interpretability, and deployment feasibility.

---

4. Overstatement of real world wearable deployment readiness

Although framed as wearable driven, model training and evaluation rely on wearable signals collected under controlled laboratory conditions, with targets derived from simulations informed by laboratory grade measurements. No field based validation is provided.

Recommendation:

- Reframe claims to reflect a laboratory based proof of concept unless additional evidence is provided.

- If possible, include robustness analyses simulating real world noise, sensor misplacement, or variable running contexts.
- Discuss expected degradation and recalibration needs in uncontrolled environments.

---

5. Data availability and reproducibility

The data availability statement does not clearly demonstrate that all numerical data and code required to reproduce analyses, figures, and model training are publicly accessible in a reusable format.

Recommendation:

- Provide access to raw or minimally processed data, derived features, labels, and metadata sufficient to reproduce all reported results.
- Release preprocessing, training, evaluation, and visualization code with fixed random seeds and environment specifications.
- Ensure the availability statement accurately reflects actual access conditions.

---

6. Statistical reporting and multiplicity

The manuscript reports numerous hypothesis tests and correlations without explicit treatment of multiple comparisons or uncertainty. Effect sizes and confidence intervals are inconsistently reported.

Recommendation:

- Address multiple testing explicitly, either through correction or clear designation of exploratory analyses.
- Report effect sizes and confidence intervals for key comparisons.
- Provide robustness checks for correlation and regression analyses, including assessment of influential observations and potential confounding.

Minor Issues

- Streamline terminology related to fatigue indices and avoid repetitive definitions.
- Reduce visual density in multi panel figures and strengthen captions to guide interpretation.
- Clarify the distinction between prediction of simulated quantities, derived fatigue indices, and clinically observable outcomes.

Other Comments

The biomechanical and fatigue modeling components are well developed and constitute the strongest aspect of the manuscript. The predictive modeling component would be substantially strengthened by improved evaluation rigor, clearer scoping of claims, and a complete reproducibility package.

Figures and Tables: Clearly presented and correctly labeled

Yes. Figures and tables are correctly labeled and generally clear, though several composite figures are overly dense.

Methods: Sufficiently detailed?

Partially. Biomechanical modeling and fatigue derivations are well described. The machine learning validation protocol, data partitioning, and hyperparameter selection procedures require greater clarity.

Study Design: Appropriate for the question?

Partially. The comparative biomechanical design is appropriate. The predictive evaluation design requires strengthening to support claims of generalizable performance.

Any experiments missing?

Yes. External validation, baseline comparisons, ablation studies, and field robustness assessments are missing.

Statistics: Statistical analyses sound?

Partially. Standard methods are used, but multiplicity control, uncertainty quantification, and dependency considerations are insufficiently addressed.

Results and data: Support conclusions?

The results support conclusions regarding altered biomechanics and elevated fatigue indices in runners with hallux valgus. They only partially support conclusions regarding scalable, wearable enabled prediction.

All data accessible?

Unclear. Data and code access should be clarified and expanded to meet reproducibility expectations.

References: Anything missing?

The reference list is appropriate for biomechanics. Additional references addressing robust predictive modeling evaluation and external validation would strengthen the manuscript.

Title: Appropriate and informative?

Yes. The title accurately reflects the scope and content of the study.
