# Supplementary material for: Structure-aware fatigue modeling in foot deformities: A digital health framework for tissue-specific running injury risk prediction using multi-modal data
Source: PLOS Digit Health. 2026 Jul 10;5(7):e0001537. doi: 10.1371/journal.pdig.0001537 (PMC13354006; doi:10.1371/journal.pdig.0001537)
Supplement: Attachment — Submitted filename: PDIG-D-25-01162R1_reviewer_JULIAN_BORGES.pdf [file pdig.0001537.s006.pdf]

Peer Review Report

Field	Value
Manuscript ID	PDIG-D-25-01162R1
Journal	PLOS Digital Health
Article Type	Research Article
Title	Structure-Aware Fatigue Modeling in Foot Deformities: A Digital Health Framework for Tissue-Specific Running Injury Risk Prediction Using Multi-Modal Data
Corresponding Author	Datao Xu, Ph.D — Ningbo University, China
Review Date	April 2026

1. Manuscript Overview

Element	Detail
Study Design	Cross-sectional case-control laboratory study
Population	52 adult runners: 26 with hallux valgus (HV; mild to moderate confirmed by radiograph) and 26 healthy controls. Mean age ~26 years. Single-centre (Ningbo University Biomechanics Laboratory, China).
Intervention / Exposure	Running at controlled speed (3.33 ± 10% m/s, overground) and 12 km/h treadmill. Three-dimensional motion capture (VICON 200 Hz), AMTI force plates (1000 Hz), wireless EMG (1000 Hz), and five Xsens IMUs.
Musculoskeletal Model	Subject-specific foot-ankle model (32 muscles, 36 ligaments) via MRI/CT reconstruction. Nonlinear strain-time-viscoelastic (NLSTV) constitutive framework. Validated against dual fluoroscopic imaging system (DFIS): ICC 0.965–0.985 across tibiotalar displacement planes.
Primary Outcomes	Cumulative Load (CL), Cumulative Damage (CD), Probability of Fatigue Failure (PFF) at four injury sites: plantar fascia, Achilles tendon, tibia, patellofemoral joint.
Deep Learning Model	SSO-CNN-BiLSTM-HAM. Model 1: IMU-only inputs (R^2 = 0.89–0.93). Model 2: IMU + HVA + FPI inputs (R^2 = 0.94–0.97). Baseline ablation: BiLSTM → CNN-BiLSTM → SSO-CNN-BiLSTM → SSO-CNN-BiLSTM-HAM. Data partitioned at subject level.
Statistical Methods	SPM-based t-tests (random field theory correction, α = 0.05) for time-series. Independent-sample t-tests (SPSS) for discrete parameters. Pearson correlation and linear regression for HVA/FPI vs fatigue indices. Multiple comparison adjustments applied for related variables.
Key Findings	HV runners showed elevated CL, CD, and PFF in Achilles tendon and plantar fascia. Tibial loading largely unchanged. HVA and FPI strongly associated with fatigue index magnitude. Deep learning model achieves high predictive accuracy for simulation-derived fatigue indices.
Revision History	R1 submission under PDIG-D-25-01162. R2 revision (current). Four reviewers at R1. Associate Editor queried financial disclosure, ethics statement format, figure filing requirements, data availability, and copyright.

2. Summary of Review

This is a second revision (R2) of a cross-sectional biomechanical-computational study integrating musculoskeletal simulation, probabilistic fatigue modeling, and hierarchical deep learning for predicting tissue-specific injury risk in runners with hallux valgus. The study is conceptually well-positioned and addresses a genuine gap at the intersection of computational biomechanics and digital health. The methodological pipeline is sophisticated and the multi-modal data acquisition is a notable strength.

The R1-to-R2 revision represents a substantive effort: the authors clarified participant-level data partitioning to address leakage concerns, added baseline model comparisons (relocated to Supplementary Material), expanded statistical reporting with RMSE and NRMSE, moderated causal and translational language, added DFIS validation metrics quantitatively, clarified the laboratory context of IMU data collection, addressed the CL/CD/PFF dissociation through the nonlinear Weibull mapping explanation, and responded appropriately to editorial formatting requirements.

Despite these improvements, the R2 revision leaves several concerns inadequately resolved. The multiple comparison correction for discrete biomechanical variables remains vague ("appropriate adjustments were applied"), without specifying method or corrected thresholds. The correlation analyses between HVA/FPI and fatigue indices are not adjusted for any confounders, and the manuscript does not formally acknowledge the consequent inferential limitations with sufficient specificity. The baseline model comparison results remain in supplementary material without summary statistics in the main text. The author summary still contains language that overstates real-world readiness. Reference numbering in the manuscript body contains a gap (reference 54 is cited in the text but reference 55 does not appear in the reference list, with numbers jumping from 54 to 56). The R2 submission remains acceptable for PLOS Digital Health contingent on addressing these outstanding concerns.

Dimension	Assessment	Change from R1
Data partitioning / leakage prevention	Satisfactorily resolved: explicit subject-level separation described in revised Methods Section 2.8	Resolved
Baseline model comparison	Provided in supplementary material. Summary results cited in main text. RMSE and NRMSE reported.	Substantially improved
Multiple comparison correction (discrete)	Vague: "appropriate adjustments were applied" — method not specified	Partially addressed
Confounder adjustment in correlations	Acknowledged as a limitation but unadjusted analyses remain primary evidence	Partially addressed
Wearable deployment framing	Clarified as laboratory proof-of-concept; limitations section expanded	Resolved
DFIS validation quantification	ICC and RMSE now provided (ICC 0.965–0.985)	Resolved
Causal language moderation	Appropriately revised to associative framing throughout revised text	Resolved
CL/CD/PFF dissociation explanation	Mechanistically explained via nonlinear Weibull mapping; satisfactory	Resolved
Reference list integrity	Reference 55 absent from list; numbers jump 54 to 56	Not resolved
Author summary language	Still contains "real-time fatigue assessment" and "real-time, non-invasive monitoring" language inconsistent with laboratory context	Not resolved
Uncertainty quantification	RMSE and NRMSE added; confidence intervals for effect sizes still absent	Partially addressed

3. Critical and Major Issues

3.1 Critical Issues

None identified at this time. No fundamental design flaw invalidating the primary conclusion has been found that is unresolvable. The subject-level partitioning claimed by the authors, if confirmed, addresses the leakage concern raised at R1.

3.2 Major Issue — Multiple Comparison Correction for Discrete Variables Remains Unspecified

**Issue ID**	M1
**Title**	Multiple Comparison Correction Method for Discrete Biomechanical Variables Not Specified
**Severity**	Major
**Description**	The revised Methods section states: "When multiple comparisons were performed across related variables, appropriate adjustments were applied to control the type I error rate." This is procedurally insufficient. The manuscript reports t-tests across multiple discrete outcome domains: anatomical indices (HVA, IMA, FPI), joint kinematics (multiple planes, multiple joints), joint kinetics, fatigue indices (CL, CD, PFF) at four tissue sites. Without specification of the correction method (e.g., Bonferroni, Benjamini-Hochberg FDR, Holm-Bonferroni), the alpha threshold applied, the number of tests included in each family, and which tests were subject to correction, the statistical reporting does not meet reproducibility standards. This issue was raised by both Reviewer 1 (statistical reporting, minor) and Reviewer 4 (Major Comment 1 and 10) at R1. The R2 response claims correction was applied but provides no specification. For a journal emphasizing transparency and reproducibility, this gap constitutes a Major deficiency.
**Recommendation**	Specify the exact multiple comparison correction procedure applied for discrete biomechanical parameter comparisons. State the number of tests in each comparison family, the correction method selected, and the corrected alpha threshold or adjusted p-values for each domain. If Bonferroni correction was used, report which variables were grouped. If FDR control was applied, specify the method (Benjamini-Hochberg or Benjamini-Yekutieli) and the q-value. This information must appear in the Methods section (Statistical Analysis subsection) and be reflected in the Results tables.

3.3 Major Issue — Correlation Analyses Between Structural Predictors and Fatigue Indices Not Adjusted for Confounders

**Issue ID**	M2
**Title**	HVA/FPI to Fatigue Index Correlation Analyses Unadjusted for Key Potential Confounders
**Severity**	Major
**Description**	The R2 revision acknowledges that the Pearson correlation and linear regression analyses between HVA/FPI and CL/CD/PFF are unadjusted for potential confounders including running speed, cadence, body mass, BMI, training volume, and sex. This was raised as a Major Comment by Reviewer 4 (Comment 2) at R1. The authors note that running speed was controlled "within a relatively narrow range" but do not quantify inter-participant speed variance or report whether speed was a covariate in any analysis. Body mass (65.2 ± 11.8 vs. 67.3 ± 9.4 kg) and weekly mileage (18.5 ± 7.3 vs. 15.8 ± 6.8 km) differ between groups with non-negligible variance and could independently influence CL, CD, and PFF. The revision characterizes the correlations as "descriptive" but does not adequately delimit what inferential conclusions can be drawn from them, and the Discussion still contains statements that frame the HVA/FPI-fatigue relationships in mechanistic terms. The absence of even partial correlation analyses controlling for body mass and running speed, which were measured and could be incorporated, is a substantive weakness.
**Recommendation**	Conduct partial correlation or simple multivariable linear regression analyses between HVA/FPI and the primary fatigue indices (CL-AT, CD-AT, CL-PF, CD-PF at minimum) adjusting for body mass and running speed as covariates. Report partial correlation coefficients or standardized regression coefficients

	alongside unadjusted Pearson r. If this analysis cannot be incorporated before the final revision deadline, the Discussion must contain explicit language clarifying the unadjusted nature of all reported associations and must refrain from inferring mechanistic specificity from these correlations.

3.4 Major Issue — Reference List Numbering Gap

**Issue ID**	M3
**Title**	Reference 55 Missing from Reference List; Numbering Jumps from 54 to 56
**Severity**	Major
**Description**	The reference list in the revised manuscript jumps from reference 54 (Wren et al., Clinical Biomechanics, 2001) to reference 56 (Jia et al., Current Molecular Pharmacology, 2024), with no reference 55 present. The in-text citation to reference 54 (regarding the Weibull modulus and Achilles tendon mechanics) and citation to reference 56 (regarding Piezo1/TRPV4 ion channels added in the R2 discussion revision at Lines 953–955) are present. The omission of reference 55 from the list may indicate either a deletion error during revision or a mis-numbered citation. This constitutes a reporting accuracy issue requiring correction. PLOS Digital Health citation standards require a complete, sequentially numbered reference list.
**Recommendation**	Reconstruct the complete reference list and identify the intended reference 55. Verify all in-text citation numbers against the reference list. If reference 55 was inadvertently deleted, restore it. If citations were renumbered during revision and the list is now internally inconsistent, perform a full citation-by-citation audit. Submit the corrected reference list with a note in the cover letter specifying the nature of the error.

4. Moderate Issues

4.1 Study Design — Author Summary Overstates Real-World Readiness

**Issue ID**	Mod1
**Title**	Author Summary Retains Real-World Monitoring Language Inconsistent with Laboratory Context
**Severity**	Moderate
**Domain**	Study Design
**Description**	The author summary (Lines 70–75 in revised manuscript) states: "enabling simple, non-invasive, and continuous monitoring outside specialized labs" and "By providing individualized, real-time fatigue assessment, this digital health framework has the potential to improve long-term foot health, reduce preventable injuries, and support large-scale screening in community and clinical settings." These claims are inconsistent with the R2 revision clarifications in the Methods and Limitations sections, where the study is correctly described as a laboratory-validated proof-of-concept with IMU data collected under controlled conditions. The author summary is published alongside the article and is read by non-specialist audiences. Its tone directly contradicts the more cautious framing now present in the body text.
**Recommendation**	Revise the author summary to align with the proof-of-concept characterization applied throughout the revised manuscript body. Replace language about monitoring "outside specialized labs" with language describing the framework as a step toward future field deployment, contingent on external validation. The author summary should accurately reflect that model inputs (IMU signals) and training targets (simulation-derived fatigue indices) were both collected under laboratory conditions.

4.2 Confounding and Bias — Effect Sizes for Group Comparisons Not Reported

**Issue ID**	Mod2
**Title**	Effect Sizes (Cohens d or Equivalent) Absent from Group Comparison Reporting
**Severity**	Moderate
**Domain**	Confounding and Bias
**Description**	The manuscript reports p-values and mean ± SD for all discrete group comparisons but does not report effect sizes (Cohen's d or equivalent). Given that the study involves a relatively small sample (n = 26 per group) and reports numerous statistically significant differences, the practical magnitude of these differences is essential for interpreting the biomechanical relevance of the findings. This is particularly important for interpreting the HV-vs-control fatigue index differences (CL, CD, PFF), where statistical significance does not necessarily imply clinically or biomechanically meaningful differentiation. Reviewer 4 (Comment 12) raised the issue of statistical transparency at R1; the R2 revision added RMSE/NRMSE for model performance but did not add effect sizes for group comparisons.
**Recommendation**	Add Cohen's d or Hedges' g effect size estimates with 95% confidence intervals for all primary group comparisons involving the fatigue indices (CL, CD, PFF) and key biomechanical variables (peak ankle moment, peak MTP moment). These may be reported within the Results section text or incorporated into Table 1 or a supplementary table. Effect size reporting is standard for PLOS Digital Health submissions and improves reproducibility.

4.3 External Validity — Severity Distribution of HV Not Characterized in Results

**Issue ID**	Mod3
**Title**	HV Severity Distribution (Mild vs. Moderate) Not Reported in Results or Analyzed as Subgroup
**Severity**	Moderate
**Domain**	External Validity
**Description**	The inclusion criteria specify both mild (HVA 15–20°; IMA 9–11°) and moderate (HVA 20–40°; IMA 11–18°) hallux valgus. The mean HVA in the HV group is 23.8 ± 5.2°, which spans both severity categories. The manuscript does not report the distribution of mild versus moderate cases nor does it examine whether fatigue index magnitudes differ by severity subgroup. This is relevant to the external validity of the framework: a fatigue prediction model calibrated predominantly on mild-to-moderate HV may not generalize to severe cases, and pooling severity levels without characterizing the composition introduces a non-trivial source of within-group heterogeneity. This concern is consistent with Reviewer 2's comment (Comment 1) at R1 regarding generalizability, though the specific severity distribution issue was not explicitly raised.
**Recommendation**	Report the number of participants in each severity subgroup (mild, moderate) and descriptive statistics stratified by severity. If sample sizes permit, report whether CL, CD, and PFF differ between severity subgroups as an exploratory analysis, clearly marked as hypothesis-generating. If subgroup analysis is not feasible due to sample size constraints, explicitly acknowledge the heterogeneous severity composition and its implications for generalizability.

5. Minor Issues

ID	Location	Issue	Suggested Action
min1	Abstract, Line 46	Inconsistent capitalization: "Hallux valgus Angle (HVA)" — the word "valgus" is lowercase mid-sentence but "Angle" is capitalized. This is inconsistent with standard anatomical nomenclature.	Standardize to "Hallux Valgus Angle (HVA)" or all-lowercase "hallux valgus angle (HVA)" throughout.

ID	Location	Issue	Suggested Action
min2	Methods Section 2.6	The tissue-specific weighting factor b is reported as 9.3 for soft tissues and 7.0 for bone in the Methods, but Supplementary Text 4 may contain different notation. Cross-check consistency between main text and supplementary equations.	Confirm that b values and their references are consistent between Equations 5, Supplementary Text 4, and the referenced source (Firminger et al., 2020).
min3	Methods Section 2.8 / Supplementary Text 6	The baseline comparison results (S6, S7 figures) are presented only as scatter plots and RMSE/NRMSE bar charts. No tabulated numerical summary of model performance metrics (R^2, RMSE, NRMSE per model, per tissue, per fatigue index) is provided. Extracting quantitative values from figures is imprecise.	Add a supplementary table summarizing all model performance metrics numerically for all four model variants, all four tissue sites, and all three fatigue indices (CL, CD, PFF).
min4	Results — Fatigue Index Tables	The Results section reports fatigue index comparisons but does not provide the absolute magnitude of CL, CD, and PFF values for each tissue in each group. Only p-values and direction of differences are readable from the text; the actual magnitude of these biomechanical variables is necessary for cross-study comparison.	Ensure Table reporting CL, CD, PFF mean ± SD per group per tissue site is present in the main manuscript or supplementary. This table should appear in the Results section, not only as figures.
min5	Discussion	The mechanobiological discussion added at R2 (Piezo1, TRPV4, skeletal muscle atrophy) is inserted briefly with minimal elaboration and two new references. This reads as superficial addition rather than integrated interpretation.	Either integrate the mechanobiological discussion more substantively (2–3 sentences expanding on how observed fatigue trajectories may relate to mechanosensitive signaling) or remove it as it currently adds narrative inconsistency.
min6	Data availability statement	The Figshare URL contains a malformed DOI prefix: "https://10.6084/..." should be "https://doi.org/10.6084/..." The current URL will not resolve correctly.	Correct the Figshare URL to include the full DOI resolver prefix.

6. Statistical and Methodological Review

Element	Assessment	Notes
Sample size justification	Adequate	G*Power estimation performed (effect size = 0.26, $\alpha = 0.05$, power = 0.8). Yields n = 26 per group.
Group matching	Adequate	Age, height, weight, weekly mileage, and running experience are balanced and reported. No significant between-group differences in confounders.
SPM time-series analysis	Adequate	Random field theory correction at $\alpha = 0.05$ applied. This is the appropriate framework for continuous biomechanical waveform comparisons.
Discrete variable testing	Partially adequate — method unspecified	Independent-sample t-tests reported. Multiple comparison correction stated but method not identified. See M1.
Correlation analyses	Partially adequate — unadjusted	Pearson r and linear regression used. No covariate adjustment. See M2.

Element	Assessment	Notes
Data partitioning (deep learning)	Adequate — as described	Subject-level stratification confirmed. Hyperparameter optimization performed within training set only. Test set held out.
Model validation	Adequate for internal; No external validation	DFIS provides kinematics validation (ICC 0.965–0.985). Internal cross-validation used for deep learning. No external cohort available.
Model performance reporting	Improved — R2, RMSE, NRMSE reported	Confidence intervals for predictive metrics remain absent. Effect sizes for group comparisons absent. See Mod2.
Fatigue model parameters	Adequate — documented in supplementary	Weibull parameters, elastic modulus, viscosity coefficient documented. b-value tissue specificity reported.
Reproducibility	Improved — code and data public	Figshare repository and GitHub code made available. URL formatting error in data availability statement. See min6.

7. Presentation and Clarity

Element	Assessment	Recommendation
Title	Appropriate	No change required.
Abstract	Improved — sample size added	Capitalization error in "Hallux valgus Angle" (see min1).
Author summary	Inconsistent with body text framing	Revise to remove "real-time monitoring outside the lab" language. See Mod1.
Introduction	Well structured; clinical context expanded in R2	No major issues.
Methods	Substantially improved in R2 for data partitioning and IMU context	Multiple comparison correction remains vague. See M1.
Results	Model performance improved; absolute fatigue values should be tabulated	Add summary table for CL/CD/PFF mean values. See min4.
Discussion	Causal language appropriately moderated	Mechanobiological addition requires better integration. See min5.
Figures	Multi-panel figures present in supplementary; density improved per R1 feedback	Tabulated summary of model performance should supplement scatter plots. See min3.
References	Reference 55 missing from list	Audit required. See M3.
Supplementary material	Substantive; DFIS validation, baseline comparisons, fatigue parameters included	Numerical performance summary table should be added. See min3.

8. Abstract and Title Review

Abstract Element	Status	Comment
Background / motivation	Present and adequate	HV-fatigue mechanistic gap clearly articulated.
Population and design	Present — improved in R2	Sample size (26 per group) now stated per Reviewer 2 feedback.
Methods summary	Present	Musculoskeletal model and deep learning approach described.
Primary results	Present	CL, CD, PFF elevation in Achilles tendon and plantar fascia. R^2 values for both model variants reported.
Limitations in abstract	Absent	PLOS Digital Health does not require limitations in abstract. Acceptable.
Causal language	Appropriately hedged	Abstract uses "associated" and "accelerate tissue fatigue across stages" — the latter verges on causal but is defensible in context.
Nomenclature consistency	Minor error	"Hallux valgus Angle (HVA)" — inconsistent capitalization. See min1.
Short title	Acceptable	Describes wearable sensing and ML. Does not overclaim.

9. References and Citations

Assessment Area	Finding
Reference list completeness	CRITICAL GAP: Reference 55 is absent from the reference list. Numbers jump from 54 to 56. This must be corrected before acceptance. See M3.
New R2 references	References 37 (Cudejko et al., 2022, Xsens validation), 54 (Jia et al., 2024, Piezo1/TRPV4), 59 (Wang et al., 2022, skeletal muscle atrophy) added appropriately. Reference 43 (Zhang et al., 2026, SJMSS) is a 2026 publication — confirm it is in press/published and that the citation is complete.
DOI accuracy	One malformed URL in Data Availability Statement: "https://10.6084/..." should be "https://doi.org/10.6084/...". See min6. DOIs in reference list appear correctly formatted.
Self-citation density	Moderate: Several first-author group publications (Zhou et al., 2024; Zhou et al., 2026 TNSRE; Zhang et al., 2026 SJMSS) cited. Density is not inappropriate for a methodologically continuous research program but is notable.
Currency and relevance	Reference list is current (2024–2026 publications included). Foundational fatigue modeling references (Edwards, Drew, Firminger, Pattin) are appropriate.
