# Supplementary material for: Structure-aware fatigue modeling in foot deformities: A digital health framework for tissue-specific running injury risk prediction using multi-modal data
Source: PLOS Digit Health. 2026 Jul 10;5(7):e0001537. doi: 10.1371/journal.pdig.0001537 (PMC13354006; doi:10.1371/journal.pdig.0001537)
Supplement: Attachment — Submitted filename: Response to Editor and Reviewers.docx [file pdig.0001537.s005.docx]

**Response to the Reviewers' Comments**

**Manuscript Number: PDIG-D-25-01162**

**Dear Editors and Reviewers:**

Thank you very much for your constructive comments and time spent analyzing our Manuscript entitled "**Structure-Aware Fatigue Modeling in Foot Deformities: A Digital Health Framework for Tissue-Specific Running Injury Risk Prediction Using Multi-Modal Data**". Those comments are all valuable and very helpful for revising and improving our paper, as well as providing important guiding significance to our research. We have studied the comments carefully and made corrections which we hope to meet with approval. Revised portions are highlighted in red on the Manuscript. Here below is our description of the revision according to the reviewers' comments.

**Response to Associate Editor: Pages 1-4.**

**Response to Reviewer 1: Pages 5-23.**

**Response to Reviewer 2: Pages 24-29.**

**Response to Reviewer 3: Pages 30-40.**

**Response to Reviewer 4: Pages 41-65.**

**Associate Editor**

**1. Please amend your detailed Financial Disclosure statement. This is published with the article. It must therefore be completed in full sentences and contain the exact wording you wish to be published.**

**Response:** Thank you for your helpful comment. We have carefully revised the Financial Disclosure statement to ensure that it is presented in complete sentences and contains the exact wording intended for publication. In the revised version, we have clearly specified the funding sources and explicitly described their respective roles in the study within the Financial Disclosure section itself, as required. We believe that the revised statement now fully complies with the journal’s formatting and transparency requirements.

**Revisions: Page 38-39 Lines 757-767**

This study was supported by multiple funding sources. The Zhejiang Provincial Natural Science Foundation of China for Distinguished Young Scholars (LR22A020002), the Zhejiang Provincial Key Research and Development Program of China (2023C03197), the Ningbo Key R&D Program (2022Z196), the National Key Research and Development Program of China (2024YFC3607305), and the Zhejiang Rehabilitation Medical Association Scientific Research Special Fund (ZKKY2023001) were supported by Yaodong Gu (YG), who contributed to the study through conceptualization, methodology development, supervision, and manuscript revision. The Ningbo 2035 Science and Technology Innovation Yong Jiang Key Technology Breakthrough Plan (2024Z200) was supported by Yi Yuan (YY), who contributed to the study through methodology development, data analysis, and manuscript revision.

**2. Please ensure that your Ethics Statement is available in its entirety at the beginning of your Methods section, under a subheading 'Ethics Statement'. It must include:**

**Response:** Thank you for your helpful comment, and we apologize for this oversight in the original submission. We have carefully revised the manuscript to ensure that the Ethics Statement is presented in its entirety at the beginning of the Methods section under the subheading “Ethics Statement.” The statement has been completed with all required information to fully comply with the journal’s guidelines. We appreciate your guidance in improving the clarity and completeness of the manuscript.

**Revisions: Page 12 Lines 209-212**

**2.1 Ethics statement**

The study protocol was approved by the Ethics Committee of Ningbo University (Approval No. RAGH20231006), and all participants provided informed consent before participating in any study activities.

**3. Please upload separate figure files in .tif or .eps format. Also, remove the figures from your manuscript file but keep the legends.**

**Response:** Thank you for your helpful comment, and we apologize for this oversight in the original submission. We have revised the manuscript accordingly. All figures have now been uploaded as separate files in the required format. In addition, the figures have been removed from the main manuscript file, while the corresponding figure legends have been retained as requested. We appreciate your guidance in helping us improve the manuscript presentation.

**4. We have noticed that you have uploaded Supporting Information files, but you have not included a list of legends. Please add a full list of legends for your Supporting Information files after the references list.**

**Response:** Thank you for pointing this out, and we regret that this detail was not properly addressed in the original submission. The manuscript has now been updated to include a complete set of legends for all Supporting Information files. These have been organized and placed immediately after the references section in accordance with the journal’s formatting requirements. We appreciate your careful review, which has helped us improve the overall clarity and structure of the submission.

**5. We note that your Data Availability Statement is currently as follows: The lower limb musculoskeletal models are available at https://simtk.org/projects/ligament_dct; https://simtk.org/projects/foot-anklemodel. All data relevant to the current study are included in the article and supplementary materials. Please confirm at this time whether or not your submission contains all raw data required to replicate the results of your study. Authors must share the “minimal data set” for their submission. PLOS defines the minimal data set to consist of the data required to replicate all study findings reported in the article, as well as related metadata and methods.**

**Response:** Thank you for your careful review and for highlighting the importance of data transparency and reproducibility. We fully agree with PLOS data sharing requirements and have taken additional steps to ensure that all materials necessary to replicate our study are openly accessible.

We confirm that our submission includes the complete minimal dataset as defined by PLOS. This comprises all raw experimental data, processed data, metadata, and methodological details required to reproduce the results presented in the manuscript, including the musculoskeletal simulations and fatigue modeling outcomes. In addition, the Figshare repository contains complete raw datasets along with relevant metadata, while the GitHub repository provides the full implementation of the prediction models used in this study. Together, these resources enable full reproducibility of the reported results.

We hope this clarification addresses the reviewer’s concern, and we remain fully committed to transparency and open scientific practices.

**Revisions: Page 39 Lines 770-776**

The lower limb musculoskeletal models are available at https://simtk.org/projects/ligament_dct; https://simtk.org/projects/foot-anklemodel. All original data are available at https://10.6084/m9.figshare.31859476.All data relevant to the current study are included in the article and supplementary materials. All prediction model codes relevant to this study are available at: https://github.com/zhifengzhou0125/Structure-Aware-Fatigue-Modeling-in-Foot-Deformities.

**6. Some material included in your submission may be copyrighted. According to PLOS’s copyright policy, authors who use figures or other material (e.g., graphics, clipart, maps) from another author or copyright holder must demonstrate or obtain permission to publish this material under the Creative Commons Attribution 4.0 International (CC BY 4.0) License used by PLOS journals. Please closely review the details of PLOS’s copyright requirements here: PLOS Licenses and Copyright. If you need to request permissions from a copyright holder, you may use PLOS's Copyright Content Permission form.**

**Response:** We appreciate the clarification regarding the journal’s copyright requirements and have carefully reviewed all materials included in our submission. All figures, graphical elements, and visual materials presented in this manuscript were independently generated by the authors using our own experimental data, modeling outputs, and visualization processes. No third-party copyrighted content has been used in the preparation of this work. Therefore, no additional permissions are required. We confirm that all submitted materials fully comply with the Creative Commons Attribution 4.0 International (CC BY 4.0) License adopted by PLOS journals.

**Reviewer 1:**

**This manuscript presents a structure aware digital health framework linking hallux valgus related morphology and foot posture to tissue specific mechanical fatigue during running. The study integrates subject specific musculoskeletal simulation incorporating dense connective tissue mechanics with probabilistic fatigue modeling to derive cumulative load, cumulative damage, and probability of fatigue failure across four injury relevant regions. A deep learning model is then trained to predict simulated tissue forces and derived fatigue indices from wearable inertial signals, with further performance gains reported when hallux valgus angle and Foot Posture Index are incorporated as structural inputs. The work is conceptually strong and aligned with the aims of digital health and biomechanics, particularly in its attempt to translate laboratory grade mechanistic understanding into a wearable enabled prediction framework. However, substantive concerns related to validation rigor, model complexity, reproducibility, and inferential framing limit confidence in generalizable predictive performance and real world applicability.**

**Response:** Thank you for the reviewer’s careful evaluation and for recognizing the conceptual contribution of this work in integrating subject-specific musculoskeletal modeling, tissue-level fatigue analysis, and wearable-driven predictive modeling. We appreciate the reviewer’s constructive concerns regarding validation rigor, model complexity, reproducibility, and the framing of translational claims. In response, we have substantially revised the manuscript to strengthen the methodological transparency and robustness of the predictive framework. Specifically, we clarified the data partitioning strategy with strict participant-level separation, expanded the reporting of predictive performance with additional evaluation metrics and uncertainty estimates, included baseline model comparisons and ablation analyses, and moderated statements regarding real-world wearable deployment. In addition, we improved the reproducibility description by providing more detailed information on data processing and model training procedures. These revisions aim to enhance the reliability and interpretability of the proposed framework while aligning the manuscript more closely with the standards expected for reproducible digital health research.

**Major Issues**

**1. Validation rigor and control of information leakage**

**The manuscript does not clearly specify whether model training, validation, and testing were conducted using strict participant-level separation. Given the use of repeated trials and multichannel time series data, any non-participant level splitting could introduce information leakage and inflate performance estimates. The reported performance metrics necessitate explicit confirmation that leakage was prevented.**

**Response:** Thank you for highlighting the importance of rigorous validation procedures when working with repeated trials and multichannel time-series biomechanical data. We agree that inappropriate data partitioning may introduce information leakage and artificially inflate model performance. The manuscript has been revised to clarify the validation protocol and data partitioning strategy.

The dataset consisted of 52 participants (26 individuals with hallux valgus and 26 healthy controls). Each participant completed ten running trials, resulting in a total of 520 trials. Data partitioning was performed at the participant level rather than the trial level. All trials from a given participant were assigned exclusively to a single dataset split. Consequently, no participant contributed data to more than one subset of the dataset, preventing subject-specific patterns from appearing simultaneously in the training and testing sets. Model evaluation was conducted using participant-stratified cross-validation, ensuring balanced representation of the hallux valgus and control groups across folds. Because each participant contributed multiple trials, all trials from the same participant were assigned to the same fold during cross-validation. This subject-wise validation strategy ensured that model performance reflected generalization to previously unseen individuals rather than unseen trials from previously observed participants. Hyperparameter optimization was performed exclusively within the training data using an internal validation procedure. The test set remained fully independent and was used only for final performance evaluation. No information from the test data was used during model development.

The description of the data partitioning and validation strategy has been clarified in the **Methods section (Section 2.8)** of the revised manuscript.

**Revisions: Page 19-20 Lines 363-388**

To evaluate ligament loading and fatigue-related injury risk across four common running injury sites, we developed a multimodal deep learning framework that integrates wearable-sensor data with subject-specific musculoskeletal simulation outputs. Time-normalized acceleration waveforms collected from foot, tibial, and femoral IMUs (${101}_{data points}$) served as the primary inputs, while the predicted outputs included tissue forces for the plantar fascia, Achilles tendon, tibia, and patellofemoral joint. Each waveform was resampled to 101 points to represent the complete gait cycle, forming multichannel time-series inputs for model training. All input channels were normalized prior to training to reduce scale-related bias.

The dataset consisted of 52 participants, including 26 individuals with hallux valgus and 26 healthy controls. All data were collected under controlled laboratory conditions during instrumented running experiments, where wearable IMU signals and laboratory-based biomechanical measurements were recorded simultaneously. Each participant completed ten running trials, resulting in a total of 520 biomechanical trials. For each trial, synchronized multichannel IMU signals were recorded from sensors placed on the foot, tibia, and femur. In parallel, laboratory biomechanical measurements were used to perform subject-specific musculoskeletal simulations to estimate tissue loading profiles. Based on these simulations, fatigue-related biomechanical indices (CL, CD, and PFF) were derived for four common running injury sites: the plantar fascia, Achilles tendon, tibia, and patellofemoral joint. These simulation-derived indices served as reference targets for supervised learning. The resulting dataset therefore represents a multimodal biomechanical dataset, combining wearable time-series sensor signals with biomechanical fatigue metrics derived from musculoskeletal simulations.

**2. Model complexity relative to sample size**

**The proposed architecture combines multiple high capacity components and an optimization routine, yet the cohort size is limited. This raises substantial concern regarding overfitting and model stability. Performance is reported without uncertainty measures, further limiting interpretability.**

**Response:** Thank you for this important comment regarding model complexity, sample size, and potential overfitting. We acknowledge that the cohort size in this study is relatively limited (52 participants), which may raise concerns regarding model generalization when using a high-capacity deep learning architecture. However, we would like to clarify several aspects of our modeling strategy.

First, the dataset comprises 52 participants with multiple gait trials and synchronized IMU and biomechanical signals, resulting in a relatively rich time-series dataset for model training. To ensure proper statistical independence and avoid data leakage, all data partitioning was performed at the subject level, such that data from each participant were included exclusively in either the training or testing set.

Second, the selection of the proposed SSO–CNN–BiLSTM–HAM architecture was not arbitrary. Prior to finalizing the model, we conducted systematic comparisons with multiple baseline models (e.g., conventional CNN, LSTM-based models, and hybrid architectures). These comparisons consistently showed that the proposed framework achieved higher predictive accuracy and lower errors, supporting its suitability for capturing the nonlinear and temporally dependent characteristics of fatigue-related biomechanical signals. Due to space limitations, these results were not fully presented in the original submission but have now been clarified in the revised manuscript.

Third, several strategies were employed to mitigate overfitting and improve model stability. These include subject-level data separation, the use of multiple gait cycles to enhance signal robustness, and consistency checks across multiple fatigue indicators (CL, CD, and PFF). The fact that consistent trends were observed across different tissues and metrics further supports the robustness of the model rather than isolated overfitting behavior.

Finally, regarding uncertainty reporting, we acknowledge that interval-based metrics (e.g., confidence intervals) were not systematically included in the original manuscript. In the revised version, we have complemented the evaluation by incorporating multiple performance metrics (R², MAE, RMSE) and baseline model comparisons, which provide a more comprehensive assessment of model performance. We agree that further incorporation of uncertainty quantification methods will be an important direction for future work, particularly in larger and more diverse datasets.

**Revisions: Page 19-20 Lines 363-388**

To evaluate ligament loading and fatigue-related injury risk across four common running injury sites, we developed a multimodal deep learning framework that integrates wearable-sensor data with subject-specific musculoskeletal simulation outputs. Time-normalized acceleration waveforms collected from foot, tibial, and femoral IMUs (${101}_{data points}$) served as the primary inputs, while the predicted outputs included tissue forces for the plantar fascia, Achilles tendon, tibia, and patellofemoral joint. Each waveform was resampled to 101 points to represent the complete gait cycle, forming multichannel time-series inputs for model training. All input channels were normalized prior to training to reduce scale-related bias.

The dataset consisted of 52 participants, including 26 individuals with hallux valgus and 26 healthy controls. All data were collected under controlled laboratory conditions during instrumented running experiments, where wearable IMU signals and laboratory-based biomechanical measurements were recorded simultaneously. Each participant completed ten running trials, resulting in a total of 520 biomechanical trials. For each trial, synchronized multichannel IMU signals were recorded from sensors placed on the foot, tibia, and femur. In parallel, laboratory biomechanical measurements were used to perform subject-specific musculoskeletal simulations to estimate tissue loading profiles. Based on these simulations, fatigue-related biomechanical indices (CL, CD, and PFF) were derived for four common running injury sites: the plantar fascia, Achilles tendon, tibia, and patellofemoral joint. These simulation-derived indices served as reference targets for supervised learning. The resulting dataset therefore represents a multimodal biomechanical dataset, combining wearable time-series sensor signals with biomechanical fatigue metrics derived from musculoskeletal simulations.

**Revisions: Page 24, Lines 463-472**

Building upon this validated biomechanical framework, we further evaluated the effectiveness of the proposed deep learning architecture. Specifically, a series of baseline and progressively enhanced models were constructed, including BiLSTM, CNN–BiLSTM, SSO–CNN–BiLSTM, and SSO–CNN–BiLSTM–HAM. These models represent a hierarchical integration of temporal modeling, spatial–temporal feature extraction, parameter optimization, and attention-based feature refinement. Comparative results show a consistent reduction in prediction errors as model complexity increases, with the proposed SSO–CNN–BiLSTM–HAM achieving the lowest RMSE and NRMSE values. Detailed model configurations and extended comparative results are provided in **Supplementary Text 6 (Figures S6–S7).**

**Revisions: Supplementary Text 6, Lines 957-988**

To evaluate the necessity and effectiveness of the proposed architecture, we first conducted systematic comparisons with several baseline models, including standalone CNN, BiLSTM, and their simplified hybrid variants. These baseline models were designed to represent commonly used structures for handling spatial or temporal features in biomechanical time-series data. Performance was evaluated using RMSE and NRMSE to ensure both absolute and normalized error assessment.

Building upon these baseline configurations, the proposed SSO–CNN–BiLSTM–HAM framework was designed to progressively integrate spatial feature extraction, temporal sequence modeling, and adaptive feature weighting. Specifically, the CNN module was employed to capture local spatial patterns in the input signals and to learn feature-level importance weights. These weights reflect the relative contribution of different biomechanical variables. The extracted features were then passed to the BiLSTM module, which models bidirectional temporal dependencies across the gait cycle. Compared to unidirectional recurrent structures, BiLSTM enables a more comprehensive representation of time-dependent biomechanical dynamics. The outputs of the BiLSTM were further combined with the CNN-derived feature weights via a dot-product operation, allowing interaction between spatial importance and temporal evolution. Subsequently, a hybrid attention mechanism (HAM) was introduced to adaptively reweight the fused features, enhancing the model’s ability to focus on critical gait phases and key fatigue-related variables. This attention-based refinement improves feature discrimination under complex and noisy biomechanical conditions. Finally, the integrated features were processed through a linear layer and a Sigmoid activation function to introduce nonlinearity, followed by a Softmax function for output normalization and prediction.

The comparative results demonstrated that the proposed model consistently outperformed all baseline models, achieving lower RMSE and NRMSE values. This indicates that the hierarchical integration of spatial and temporal features, combined with attention-based weighting, provides a measurable advantage in predicting fatigue-related biomechanical indicators. These findings support the rationale for adopting the proposed architecture, particularly under limited sample conditions where efficient feature utilization is critical (Figures S6–S7).

**Fig. S6** Model prediction errors results. (A) Prediction errors of CD based on BiLSTM model. (B) Prediction errors of CD based on CNN-BiLSTM model. (C) Prediction errors of CD based on SSO–CNN-BiLSTM model. (D) Prediction errors of CD based on SSO–CNN-BiLSTM-HAM model. (E) Results of RMSE. (F) Results of NRMSE.

**Fig. S7** Model prediction errors results. (A) Prediction errors of PFF based on BiLSTM model. (B) Prediction errors of PFF based on CNN-BiLSTM model. (C) Prediction errors of PFF based on SSO–CNN-BiLSTM model. (D) Prediction errors of PFF based on SSO–CNN-BiLSTM-HAM model. (E) Results of RMSE. (F) Results of NRM

**3. Absence of baseline model comparisons**

**The manuscript does not demonstrate that the proposed model materially outperforms simpler alternatives. Without appropriate baselines, the added complexity of the model cannot be adequately justified.**

**Response:** Thank you for this important suggestion. We agree that comparisons with simpler baseline models are necessary to justify the architectural complexity of the proposed framework.

In related methodological work, different deep learning architectures—including BiLSTM, CNN–BiLSTM, and SSO–CNN–BiLSTM models—have been explored to evaluate their capability in capturing biomechanical time-series features. In the original submission of this manuscript, these comparisons were not included due to space constraints, as the primary focus was on the proposed structure-aware fatigue prediction framework. Following the reviewer’s suggestion, we have now incorporated these baseline models into the revised manuscript to provide a more comprehensive evaluation. Specifically, we compared four model configurations with progressively increasing architectural complexity: BiLSTM, CNN–BiLSTM, SSO–CNN–BiLSTM, and the proposed SSO–CNN–BiLSTM–HAM model. The results show that the proposed model consistently achieved the best predictive performance for fatigue-related indices (CL, CD, and PFF). These comparisons have now been added to the Supplementary Materials.

**Revisions: Page 20 Lines 400-413**

As illustrated in **Figure 1D**, the deep learning framework was developed through a sequential integration of multiple computational components for modeling biomechanical time-series data derived from wearable sensors and laboratory-based biomechanical analysis. The model architecture was progressively constructed through four configurations with increasing structural complexity. First, a BiLSTM model was implemented to capture temporal dependencies within multichannel biomechanical time-series signals. Next, a CNN–BiLSTM model was developed by introducing convolutional layers to extract local spatial features from the wearable-sensor signals before temporal modeling. Subsequently, a SSO–CNN–BiLSTM model was constructed by incorporating the Sparrow Search Optimization (SSO) algorithm to optimize key hyperparameters of the network during training. Finally, the SSO–CNN–BiLSTM–HAM model was implemented by integrating a Hybrid Attention Mechanism (HAM), enabling the network to adaptively weight temporally and spatially relevant features within the input signals.

**Revisions: Page 24, Lines 463-472**

Building upon this validated biomechanical framework, we further evaluated the effectiveness of the proposed deep learning architecture. Specifically, a series of baseline and progressively enhanced models were constructed, including BiLSTM, CNN–BiLSTM, SSO–CNN–BiLSTM, and SSO–CNN–BiLSTM–HAM. These models represent a hierarchical integration of temporal modeling, spatial–temporal feature extraction, parameter optimization, and attention-based feature refinement. Comparative results show a consistent reduction in prediction errors as model complexity increases, with the proposed SSO–CNN–BiLSTM–HAM achieving the lowest RMSE and NRMSE values. Detailed model configurations and extended comparative results are provided in **Supplementary Text 6 (Figures S6–S7).**

**Revisions: Supplementary Text 6, Lines 957-988**

To evaluate the necessity and effectiveness of the proposed architecture, we first conducted systematic comparisons with several baseline models, including standalone CNN, BiLSTM, and their simplified hybrid variants. These baseline models were designed to represent commonly used structures for handling spatial or temporal features in biomechanical time-series data. Performance was evaluated using RMSE and NRMSE to ensure both absolute and normalized error assessment.

Building upon these baseline configurations, the proposed SSO–CNN–BiLSTM–HAM framework was designed to progressively integrate spatial feature extraction, temporal sequence modeling, and adaptive feature weighting. Specifically, the CNN module was employed to capture local spatial patterns in the input signals and to learn feature-level importance weights. These weights reflect the relative contribution of different biomechanical variables. The extracted features were then passed to the BiLSTM module, which models bidirectional temporal dependencies across the gait cycle. Compared to unidirectional recurrent structures, BiLSTM enables a more comprehensive representation of time-dependent biomechanical dynamics. The outputs of the BiLSTM were further combined with the CNN-derived feature weights via a dot-product operation, allowing interaction between spatial importance and temporal evolution. Subsequently, a hybrid attention mechanism (HAM) was introduced to adaptively reweight the fused features, enhancing the model’s ability to focus on critical gait phases and key fatigue-related variables. This attention-based refinement improves feature discrimination under complex and noisy biomechanical conditions. Finally, the integrated features were processed through a linear layer and a Sigmoid activation function to introduce nonlinearity, followed by a Softmax function for output normalization and prediction.

The comparative results demonstrated that the proposed model consistently outperformed all baseline models, achieving lower RMSE and NRMSE values. This indicates that the hierarchical integration of spatial and temporal features, combined with attention-based weighting, provides a measurable advantage in predicting fatigue-related biomechanical indicators. These findings support the rationale for adopting the proposed architecture, particularly under limited sample conditions where efficient feature utilization is critical **(Figures S6–S7).**

**Fig. S6** Model prediction errors results. (A) Prediction errors of CD based on BiLSTM model. (B) Prediction errors of CD based on CNN-BiLSTM model. (C) Prediction errors of CD based on SSO–CNN-BiLSTM model. (D) Prediction errors of CD based on SSO–CNN-BiLSTM-HAM model. (E) Results of RMSE. (F) Results of NRMSE.

**Fig. S7** Model prediction errors results. (A) Prediction errors of PFF based on BiLSTM model. (B) Prediction errors of PFF based on CNN-BiLSTM model. (C) Prediction errors of PFF based on SSO–CNN-BiLSTM model. (D) Prediction errors of PFF based on SSO–CNN-BiLSTM-HAM model. (E) Results of RMSE. (F) Results of NRM

**4. Overstatement of real world wearable deployment readiness Although framed as wearable driven, model training and evaluation rely on wearable signals collected under controlled laboratory conditions, with targets derived from simulations informed by laboratory grade measurements. No field based validation is provided.**

**Response:** Thank you very much for this insightful and constructive comment. We sincerely apologize for the lack of clarity in the original manuscript regarding the experimental context in which the wearable data were collected. We greatly appreciate the reviewer highlighting this point, as it helped us improve the accuracy and transparency of the study description.

In the present study, wearable IMU signals and laboratory-based biomechanical measurements were collected simultaneously under controlled laboratory conditions during instrumented running trials. Specifically, IMU sensors mounted on the foot, tibia, and femur were used to record wearable motion signals, while laboratory-grade biomechanical measurements were obtained to support musculoskeletal modeling. Each movement condition was repeated across multiple trials for each participant, allowing synchronized wearable and biomechanical datasets to be collected for the same running tasks. The laboratory measurements were subsequently used to perform subject-specific musculoskeletal simulations to estimate tissue loading profiles. From these simulations, fatigue-related biomechanical indices (CL, CD, and PFF) were derived for four common running injury sites: the plantar fascia, Achilles tendon, tibia, and patellofemoral joint. These simulation-derived variables served as reference targets for supervised model training, whereas the wearable IMU signals were used as the primary input features of the deep learning framework. In this way, the model was designed to learn the relationship between wearable motion signals and biomechanical fatigue indicators that are typically obtained through laboratory-based biomechanical analysis, potentially enabling their estimation from wearable measurements.

Following the reviewer’s suggestion, we have revised the manuscript to clarify that the wearable signals used in this study were collected within a laboratory-based experimental setting, where they were synchronized with high-quality biomechanical measurements. The relevant descriptions in the Methods section have been updated accordingly to ensure that the experimental conditions and data acquisition procedures are presented more explicitly. In addition, we have expanded the Limitations section to discuss potential challenges associated with applying the proposed framework in uncontrolled running environments. In real-world settings, additional sources of variability may arise from factors such as sensor placement differences, environmental noise, and variations in running context. These factors may influence prediction stability and may require further robustness evaluation or model recalibration. The revised manuscript therefore highlights that future studies should evaluate the performance of the proposed framework using field-based wearable recordings and more diverse running environments, which will be important for assessing the generalizability and practical applicability of the approach.

Overall, these revisions were made to ensure that the scope of the study is accurately represented as a laboratory-validated multimodal biomechanical prediction framework that integrates wearable sensor signals with laboratory-derived biomechanical references, rather than a fully field-deployed wearable monitoring system. We thank the reviewer again for this helpful suggestion, which has improved the clarity and rigor of the manuscript.

**Revisions: Page14 Lines 240-246**

In addition, five wearable inertial measurement units (IMUs; Xsens, Henderson, USA) were attached to the lower limbs to record multichannel acceleration signals. Sensors were placed on the foot and at the distal and proximal segments of the tibia and femur to capture segmental motion during running. The IMU system was synchronized with the laboratory biomechanical measurements to enable integrated analysis of wearable motion signals and laboratory-based biomechanical variables.

**Revisions: Page 19-20 Lines 363-388**

The dataset consisted of 52 participants, including 26 individuals with hallux valgus and 26 healthy controls. All data were collected under controlled laboratory conditions during instrumented running experiments, where wearable IMU signals and laboratory-based biomechanical measurements were recorded simultaneously. Each participant completed ten running trials, resulting in a total of 520 biomechanical trials. For each trial, synchronized multichannel IMU signals were recorded from sensors placed on the foot, tibia, and femur. In parallel, laboratory biomechanical measurements were used to perform subject-specific musculoskeletal simulations to estimate tissue loading profiles. Based on these simulations, fatigue-related biomechanical indices (CL, CD, and PFF) were derived for four common running injury sites: the plantar fascia, Achilles tendon, tibia, and patellofemoral joint. These simulation-derived indices served as reference targets for supervised learning. The resulting dataset therefore represents a multimodal biomechanical dataset, combining wearable time-series sensor signals with biomechanical fatigue metrics derived from musculoskeletal simulations.

**Revisions: Page 34-35 Lines 698-709**

Third, although wearable IMU signals were used as the primary input features of the predictive framework, all wearable data in this study were collected under controlled laboratory conditions together with laboratory-grade biomechanical measurements. Therefore, the present framework should be interpreted as a laboratory-validated proof-of-concept rather than a fully field-deployed wearable monitoring system. In real-world running environments, additional variability may arise from factors such as sensor placement differences, environmental noise, and variations in running context, which may influence prediction stability. Future work will therefore focus on validating the framework using field-based wearable recordings and longitudinal monitoring to further enhance the ecological validity and practical applicability of the proposed fatigue prediction approach.

**5. Data availability and reproducibility The data availability statement does not clearly demonstrate that all numerical data and code required to reproduce analyses, figures, and model training are publicly accessible in a reusable format.**

**Response:** Thank you for highlighting the importance of data availability and reproducibility. We fully agree that transparent access to the underlying data and computational procedures is essential for ensuring the reliability and reproducibility of data-driven research.

Following the reviewer’s suggestion, we have clarified the Data Availability Statement in the revised manuscript. All datasets and source code used in this study have now been made publicly available in an open repository. The shared materials include the numerical datasets required to reproduce the analyses, model inputs and outputs, as well as the code used to generate the figures reported in the manuscript. We hope that making the dataset and modeling framework openly accessible will encourage other researchers to build upon this work, expand the dataset, and further explore wearable-based biomechanical fatigue prediction in running. The revised manuscript now includes an updated Data Availability Statement specifying the public repository and access details.

**Figure 1.** IMU Data

**Figure 2.** Impact Force Data

**Figure3.** Kinematic data

**6. Statistical reporting and multiplicity The manuscript reports numerous hypothesis tests and correlations without explicit treatment of multiple comparisons or uncertainty. Effect sizes and confidence intervals are inconsistently reported**

**Response:** Thank you for this valuable comment. We appreciate the reviewers’ careful evaluation of the statistical analysis procedures. We acknowledge that the original manuscript did not sufficiently clarify several aspects of the statistical methodology, which may have confused the analytical procedures.

In the revised manuscript, the Statistical Analysis section has been carefully revised to provide clearer descriptions of the analytical steps and assumptions. Specifically, we have made the following improvements:

1. Time-series statistical analysis:
For continuous waveform comparisons across the stance phase, statistical parametric mapping (SPM)-based independent-sample t-tests were performed in MATLAB. The significance threshold was determined using random field theory correction at α = 0.05.

2. Discrete parameter comparisons:
Independent-sample t-tests were conducted for discrete biomechanical parameters, including anatomical indices, joint kinematics, joint kinetics, and fatigue-related indicators (CL, CD, and PFF).

3. Association analysis:
Pearson correlation and linear regression analyses were applied to explore the relationships between foot deformity indicators (HVA and FPI) and fatigue-related biomechanical indices (CL, CD, and PFF).

These revisions have been incorporated into the Statistical Analysis section of the Methods, and the description has been clarified accordingly. Importantly, these clarifications do not affect the results or the main conclusions of the study. We thank the reviewers again for pointing out this issue and helping us improve the clarity and transparency of the statistical analysis.

**Revisions: Page 22-23 Lines 431-445**

For time-series variables across the stance phase, statistical parametric mapping (SPM)-based independent-sample t-tests were conducted in MATLAB to examine temporal differences between the healthy and HV groups [18]. The significance threshold for SPM analyses was determined using random field theory correction at α = 0.05. For discrete biomechanical parameters—including anatomical indices, foot morphology measures, peak joint angles, joint moments, joint forces, range of motion (ROM), and fatigue indicators (CL, CD, and PFF)—independent-sample t-tests were performed in SPSS to compare the healthy and HV groups during running. To further investigate the associations between foot deformity and fatigue-related biomechanical indicators, Pearson correlation and linear regression analyses were conducted to examine the relationships between hallux valgus angle (HVA), Foot Posture Index (FPI), and the fatigue indices CL, CD, and PFF [17]. The significance level was set at α = 0.05. When multiple comparisons were performed across related variables, appropriate adjustments were applied to control the type I error rate.

**Minor Issues**

**• Streamline terminology related to fatigue indices and avoid repetitive definitions.**

**Response:** Thank you for this helpful suggestion. We agree that some terminology related to the fatigue indices was repeated in several sections of the manuscript. In the revised version, the definitions of the fatigue-related biomechanical indicators—CL, CD, and PFF—have been streamlined. In subsequent sections, these variables are consistently referred to using their abbreviations (CL, CD, and PFF) and collectively described as “fatigue indices.” This modification streamlines the terminology and reduces redundancy throughout the manuscript.

**• Reduce visual density in multi-panel figures and strengthen captions to guide interpretation.**

**Response:** Thank you for this helpful suggestion. We agree that the original multi-panel figure was visually dense and could make rapid interpretation of the results more difficult. In the revised manuscript, the figure layout has been reorganized to improve clarity and reduce visual complexity. In addition, the figure caption has been substantially revised to more clearly guide readers through the structure of the panels and the interpretation of the prediction results. These revisions improve the readability of the figure and facilitate clearer comparison of the model predictions and associated errors.

**• Clarify the distinction between prediction of simulated quantities, derived fatigue indices, and clinically observable outcomes.**

**Response:** Thank you for this important comment. We agree that it is important to clearly distinguish between the prediction of simulation-derived biomechanical quantities, the derived fatigue indices, and clinically observable injury outcomes. In the revised manuscript, we have clarified this distinction in the Methods and Discussion sections. Specifically, the proposed deep learning framework uses wearable IMU signals to estimate biomechanical variables derived from subject-specific musculoskeletal simulations. These simulated tissue forces are subsequently used to compute fatigue-related indices (CL, CD, PFF), which serve as quantitative indicators of tissue mechanical loading and fatigue accumulation. Importantly, these indices are not direct clinical diagnoses of injury, but rather biomechanical proxies that may be associated with injury risk. The manuscript has been revised to explicitly reflect this distinction and avoid potential misinterpretation of the predictive outputs.

**Revisions: Page 30, Lines 592-599**

This study aims to explore the relationship between HV and foot pronation, to understand how these structural alterations influence lower-limb biomechanical loading during running. Specifically, we analyze how structural changes in the feet of runners with HV led to the development of foot pronation, and how this subsequently affects their biomechanical characteristics and athletic performance. This work focuses on three key metrics to achieve this: CL, CD, and PFF. Changes in these indicators may reflect increased mechanical loading on tissues, particularly during prolonged running sessions.

**Revisions: Page 30, Lines 609-611**

Additionally, HV patients generally had a smaller knee joint ROM, limiting their athletic ability and potentially increasing mechanical stress on the lower limb.

**Revisions: Page 32, Lines 643-645**

In this study, Model 1 demonstrated high predictive accuracy for the fatigue indices (R² = 0.89–0.93, absolute error < 0.8), with PFF showing superior fidelity compared to CL and CD.

**Revisions: Page 34, Lines 6786-689**

In summary, this study integrates biomechanical modeling with deep learning–based fatigue prediction to reveal how HV-associated structural deformities amplify tissue loading, accelerate fatigue accumulation, and influence tissue loading patterns during running.

**Reviewer 2:**

**This manuscript presents a comprehensive and methodologically sophisticated study integrating biomechanical modeling, wearable sensor data, and hierarchical deep learning to predict tissue-specific fatigue risk in runners with hallux valgus (HV). The work is timely, addresses a clear gap in the literature, and demonstrates high predictive accuracy. However, several limitations should be addressed to strengthen the manuscript.**

**Response:** Thank you for your thoughtful and encouraging evaluation of our manuscript. We sincerely appreciate your positive comments regarding the methodological integration of biomechanical modeling, wearable sensor data, and hierarchical deep learning for predicting tissue-specific fatigue characteristics in runners with hallux valgus (HV). We are pleased that the reviewer recognizes the timeliness of the study and its potential contribution to addressing an existing gap in the literature. At the same time, we fully acknowledge the limitations identified by the reviewer. Following these constructive suggestions, we have carefully revised the manuscript to improve the clarity, methodological transparency, and interpretation of the results. Specifically, we have addressed the concerns regarding model assumptions, data interpretation, and presentation of the results. Detailed point-by-point responses to each comment are provided below, and all corresponding revisions have been clearly indicated in the revised manuscript.

**1. Abstract
The abstract does not mention the sample size (n=26 per group) or acknowledge that the findings are based on a relatively small cohort, which may affect the generalizability of the proposed framework to broader populations.**

**Response:** Thank you for this valuable comment. We agree that clearly reporting the sample size in the abstract improves transparency and helps readers better understand the study context. In the revised manuscript, we have explicitly clarified the group sizes in the abstract by indicating that the study included 26 runners with hallux valgus and 26 healthy controls.

In addition, we acknowledge that the relatively small cohort may limit the generalizability of the proposed framework. This limitation has now been explicitly discussed in the Discussion section, where we note that the current study should be interpreted as a preliminary validation and that future studies with larger and more diverse cohorts are needed. Furthermore, to enhance transparency and reproducibility, the dataset used in this study has been openly shared, allowing other researchers to validate and extend the proposed framework using larger datasets in future work. All corresponding revisions have been incorporated into the revised manuscript.

**Revisions: Page 3, Lines 32-36**

This study integrated gait analysis with a nonlinear, subject-specific musculoskeletal model incorporating multi-joint coordination, muscle–tendon nonlinearity, and tissue-level cumulative damage mechanics to quantify fatigue trajectories in a cohort of 26 runners with HV and 26 healthy controls.

**Revisions: Page 34, Lines 692-693**

First, the sample size was relatively small, which may have limited the generalizability of the results to populations with varying degrees of HV severity.

**2. Introduction
While the technical gaps in fatigue modeling are well articulated, the introduction could better frame the clinical need for such a model—specifically, how it would be integrated into existing prevention or rehabilitation pathways for runners with HV.**

**Response:** Thank you for this helpful suggestion. We agree that further clarifying the potential practical relevance of the proposed framework would improve the context of the study. In the revised manuscript, we have expanded the final paragraph of the Introduction to better describe how the proposed fatigue prediction framework could support injury prevention and rehabilitation management in runners with hallux valgus. Specifically, we now highlight that such a framework may facilitate early identification of excessive tissue loading and provide quantitative biomechanical indicators to assist individualized training regulation, injury prevention, and rehabilitation monitoring. These revisions help better connect the methodological development of the model with its potential applications in clinical and sports training contexts.

**Revisions: Page 10, Lines 167-173**

Ultimately, this integrative approach bridges foot deformity with fatigue-driven injury mechanisms, advancing precision prediction and prevention in runners with HV. From a practical perspective, such a framework may support early identification of excessive tissue loading during running and provide quantitative biomechanical indicators for individualized training regulation, injury prevention, and rehabilitation monitoring in runners with foot deformities.

**3. Methods
The manuscript mentions using IMU data from Xsens sensors but provides minimal detail on how these signals were preprocessed, synchronized, or validated against the gold-standard motion capture system. This omission makes it difficult to assess the reliability of the wearable data pipeline. Additionally, validity and reliability of the sensors should be acknowledged (https://www.nature.com/articles/s41598-022-18845-x)
The subject-specific model assumes consistent ligament properties across participants, which may not capture inter-individual variability in tissue mechanics. The use of a single constitutive model (NLSTV) for all ligaments may oversimplify the diverse viscoelastic behaviors of different connective tissues**

**Response:** Thank you for this valuable comment. In the revised manuscript, we have added the recommended reference and related literature to acknowledge the validity and reliability of the Xsens IMU sensors and to better support the wearable data acquisition pipeline. Based on these studies, we have also refined the description of the IMU signal processing in the Methods section.

Regarding the modeling assumptions, we acknowledge that the current framework assumes consistent ligament properties and adopts a single NLSTV constitutive formulation for computational efficiency. We have clarified this point in the model construction description and further discussed it in the limitations section, noting that future work will incorporate more individualized tissue parameters and subject-specific modeling strategies to better capture inter-individual variability.

In addition, to promote transparency and reproducibility, the dataset used in this study has been openly shared, allowing future studies to further expand and personalize the modeling framework.

**Revisions: Page 14, Lines 240-246**

In addition, five wearable inertial measurement units (IMUs; Xsens, Henderson, USA) were attached to the lower limbs to record multichannel acceleration signals. Sensors were placed on the foot and at the distal and proximal segments of the tibia and femur to capture segmental motion during running. The IMU system was synchronized with the laboratory biomechanical measurements to enable integrated analysis of wearable motion signals and laboratory-based biomechanical variables [37].

37.	Cudejko T, Button K, Al-Amri MJSr. Validity and reliability of accelerations and orientations measured using wearable sensors during functional activities. 2022;12(1):14619.

**4. Results
While R² values are reported, other important metrics—such as mean absolute error (MAE), root mean square error (RMSE), or precision-recall for classification of high-risk individuals—are not provided. This limits the ability to fully evaluate model robustness.**

**Response:** Thank you for this helpful suggestion, and we apologize for not providing a more comprehensive set of evaluation metrics in the original version of the manuscript. In the revised manuscript, we have expanded the Results section by adding additional performance metrics, including Root Mean Square Error (RMSE) and Normalized Root Mean Square Error (NRMSE), to provide a more complete assessment of the model’s predictive accuracy and robustness. In addition, we have included comparisons with several baseline models to further evaluate the effectiveness of the proposed framework. These additions complement the previously reported R² values and allow a clearer and more comprehensive evaluation of model performance.

**5. Discussion
The discussion strongly implies that HV causes increased fatigue risk, but the study design is cross-sectional and correlational. Causal claims should be tempered, and the potential for confounding factors (e.g., running experience, training load) should be acknowledged.
While the authors note that real-time field validation was not performed, the discussion does not sufficiently address the practical challenges of deploying such a model outside the lab—such as sensor placement variability, environmental noise, or user compliance.**

**Response:** Thank you for this valuable comment. We agree that the interpretation of the results should be presented with appropriate caution given the cross-sectional design of the present study. In the revised manuscript, we have carefully refined several statements in the Discussion section to reduce overly direct causal wording and instead emphasize that the findings reflect biomechanical associations between hallux valgus–related structural characteristics and increased tissue fatigue indices during running. In particular, the relevant paragraphs discussing the relationship between HV-related structural deviation and fatigue-related indices (CL, CD, and PFF) have been revised to frame these findings as potential biomechanical mechanisms inferred from the observed differences rather than definitive causal effects. These revisions help ensure that the interpretation remains consistent with the study design while still highlighting the biomechanical insights provided by the analysis. In addition, we have further expanded the Discussion section to acknowledge that other factors may also contribute to fatigue-related injury risk. This addition provides a more balanced interpretation of the results and clarifies that HV-related structural characteristics represent one important contributing factor within a broader biomechanical context.

Furthermore, regarding the practical deployment of the proposed framework, we appreciate the reviewer’s suggestion. In the revised manuscript, we have clarified that the wearable sensor data used in this study were collected under controlled laboratory conditions, and this aspect has now been explicitly discussed in the limitations section. Accordingly, the present work should be interpreted as a laboratory-validated framework. Future research will extend this work by evaluating the robustness and applicability of the proposed approach in real-world running environments.

**Revisions: Page 30, Lines 594-599**

Specifically, we analyzed how structural characteristics of the feet in runners with HV are associated with pronation-related gait patterns, and how these variations may influence biomechanical characteristics during running. This work focuses on three key metrics to achieve this: CL, CD, and PFF. Changes in these indicators may reflect increased mechanical loading on tissues, particularly during prolonged running sessions.

**Revisions: Page 30, Lines 609-611**

Additionally, HV patients generally had a smaller knee joint ROM, which may influence running mechanics and potentially increase mechanical demands on the lower limb.

**Revisions: Page 33, Lines 661-663**

Runners with HV typically exhibit more pronounced foot pronation due to alterations in foot structure, which may influence gait mechanics and potentially contribute to increased lower-limb injury susceptibility.

**Revisions: Page 34, Lines 686-689**

In summary, this study integrates biomechanical modeling with deep learning–based fatigue prediction to reveal how HV-associated structural deformities amplify tissue loading, accelerate fatigue accumulation, and influence tissue loading patterns during running.

**Revisions: Page 34-35, Lines 692-709**

Third, although wearable IMU signals were used as the primary input features of the predictive framework, all wearable data in this study were collected under controlled laboratory conditions together with laboratory-grade biomechanical measurements. Therefore, the present framework should be interpreted as a laboratory-validated proof-of-concept rather than a fully field-deployed wearable monitoring system. In real-world running environments, additional variability may arise from factors such as sensor placement differences, environmental noise, and variations in running context, which may influence prediction stability. Future work will therefore focus on validating the framework using field-based wearable recordings and longitudinal monitoring to further enhance the ecological validity and practical applicability of the proposed fatigue prediction approach.

**Reviewer 3:**

**This manuscript proposes a digital health framework combining gait analysis, nonlinear subject-specific musculoskeletal models, and hierarchical deep learning algorithms, aimed at quantifying and predicting tissue-specific fatigue risks in runners with Hallux Valgus (HV) compared to healthy controls during running. By integrating multi-joint coordination, muscle-tendon nonlinearity, and tissue-level cumulative damage mechanics, the authors reveal specific loading patterns in HV runners under foot structural deviations (e.g., increased hallux valgus angle and foot posture index), specifically showing significantly elevated risks of cumulative damage in the Achilles tendon and plantar fascia. This study provides a potential theoretical and technical pathway for personalized sports injury prevention and remote monitoring. However, there are some limitations in the manuscript that need further refinement.**

**Response:** Thank you for the reviewer’s thoughtful and constructive comments. We appreciate the positive evaluation of the proposed framework and the recognition of the potential value of integrating gait analysis, nonlinear subject-specific musculoskeletal modeling, and hierarchical deep learning algorithms for quantifying tissue-specific fatigue risk in runners with hallux valgus. We are encouraged that the reviewer acknowledged the potential contribution of this work to personalized sports injury prevention and wearable-based monitoring strategies. Following the reviewer’s suggestions, we have carefully revised the manuscript to further improve clarity, methodological description, and interpretation of the results.

**Major Comments
1. The authors constructed a deep learning model (SSO-CNN-BILSTM-HAM) to predict fatigue trajectories; however, the sample size is relatively small (26 in the HV group and 26 in the control group). For deep learning methods, insufficient sample size may lead to model overfitting, thereby affecting its generalization ability. It is suggested that the authors further discuss the impact of small sample data on model stability, or attempt to use validation methods more suitable for small samples (such as leave-one-out cross-validation) to enhance the credibility of the results. Furthermore, although "Wearable Data" is mentioned in the title, the data for model training in the methodology section appears to be primarily derived from laboratory-grade gait analysis systems. The authors should clarify how high-dimensional data obtained solely from wearable devices in real-world application scenarios can meet the input requirements of this complex model, or provide supplementary validation analysis on the consistency between wearable sensor signals and laboratory gold-standard data.**

**Response:** Thank you for this insightful comment. We appreciate the reviewer’s concerns regarding the sample size and the use of wearable data in the proposed deep learning framework, and we have clarified these aspects in the revised manuscript.

First, regarding the sample size, the study included 52 participants (26 runners with hallux valgus and 26 healthy controls), and this information has now been explicitly stated in the revised manuscript. Importantly, each participant completed ten successful running trials, and synchronized wearable IMU signals and laboratory biomechanical measurements were collected for each trial. Therefore, the dataset used for model development consists of multiple repeated experimental trials and high-dimensional time-series sensor signals, rather than a single observation per subject. These repeated measurements and continuous IMU signal streams substantially increase the effective data volume available for model training and evaluation. In addition, to improve transparency and facilitate future research, the dataset and related resources have been made openly available to enable subsequent studies to further expand the dataset and validate the framework under broader experimental conditions.

Second, regarding the selection of the deep learning architecture, we performed comparisons with several baseline models during the model development stage to ensure that the chosen framework was suitable for the characteristics of the dataset. These preliminary evaluations indicated that the proposed SSO–CNN–BiLSTM–HAM framework consistently achieved higher predictive accuracy and lower prediction errors compared with other commonly used architectures. Therefore, this model was selected as the primary framework for fatigue prediction in this study. Due to space limitations in the original manuscript, the detailed baseline comparison results were not presented. Following the reviewer’s suggestion, we have now clarified the rationale for model selection and added relevant descriptions in the revised manuscript.

Finally, we acknowledge the reviewer’s point regarding the wearable data described in the title. In the present study, all IMU signals were collected using wearable sensors under controlled laboratory conditions, synchronized with laboratory-grade biomechanical measurements. This design was adopted to ensure signal reliability and accurate model calibration during the initial development stage. We have now clarified this point in the revised manuscript and emphasized in the limitations section that the current framework should be interpreted as a laboratory-validated proof-of-concept. Future work will aim to validate the model using fully field-based wearable recordings to further evaluate its robustness under real-world running conditions.

**Revisions: Page 19-20, Lines 366-388**

To evaluate ligament loading and fatigue-related injury risk across four common running injury sites, we developed a multimodal deep learning framework that integrates wearable-sensor data with subject-specific musculoskeletal simulation outputs. Time-normalized acceleration waveforms collected from foot, tibial, and femoral IMUs (${101}_{data points}$) served as the primary inputs, while the predicted outputs included tissue forces for the plantar fascia, Achilles tendon, tibia, and patellofemoral joint. Each waveform was resampled to 101 points to represent the complete gait cycle, forming multichannel time-series inputs for model training. All input channels were normalized prior to training to reduce scale-related bias.

The dataset consisted of 52 participants, including 26 individuals with hallux valgus and 26 healthy controls. All data were collected under controlled laboratory conditions during instrumented running experiments, where wearable IMU signals and laboratory-based biomechanical measurements were recorded simultaneously. Each participant completed ten running trials, resulting in a total of 520 biomechanical trials. For each trial, synchronized multichannel IMU signals were recorded from sensors placed on the foot, tibia, and femur. In parallel, laboratory biomechanical measurements were used to perform subject-specific musculoskeletal simulations to estimate tissue loading profiles. Based on these simulations, fatigue-related biomechanical indices (CL, CD, and PFF) were derived for four common running injury sites: the plantar fascia, Achilles tendon, tibia, and patellofemoral joint. These simulation-derived indices served as reference targets for supervised learning. The resulting dataset therefore represents a multimodal biomechanical dataset, combining wearable time-series sensor signals with biomechanical fatigue metrics derived from musculoskeletal simulations.

**Revisions: Page 24, Lines 463-472**

Building upon this validated biomechanical framework, we further evaluated the effectiveness of the proposed deep learning architecture. Specifically, a series of baseline and progressively enhanced models were constructed, including BiLSTM, CNN–BiLSTM, SSO–CNN–BiLSTM, and SSO–CNN–BiLSTM–HAM. These models represent a hierarchical integration of temporal modeling, spatial–temporal feature extraction, parameter optimization, and attention-based feature refinement. Comparative results show a consistent reduction in prediction errors as model complexity increases, with the proposed SSO–CNN–BiLSTM–HAM achieving the lowest RMSE and NRMSE values. Detailed model configurations and extended comparative results are provided in **Supplementary Text 6 (Figures S6–S7).**

**Revisions: Supplementary Text 6, Lines 957-988**

To evaluate the necessity and effectiveness of the proposed architecture, we first conducted systematic comparisons with several baseline models, including standalone CNN, BiLSTM, and their simplified hybrid variants. These baseline models were designed to represent commonly used structures for handling spatial or temporal features in biomechanical time-series data. Performance was evaluated using RMSE and NRMSE to ensure both absolute and normalized error assessment.

Building upon these baseline configurations, the proposed SSO–CNN–BiLSTM–HAM framework was designed to progressively integrate spatial feature extraction, temporal sequence modeling, and adaptive feature weighting. Specifically, the CNN module was employed to capture local spatial patterns in the input signals and to learn feature-level importance weights. These weights reflect the relative contribution of different biomechanical variables. The extracted features were then passed to the BiLSTM module, which models bidirectional temporal dependencies across the gait cycle. Compared to unidirectional recurrent structures, BiLSTM enables a more comprehensive representation of time-dependent biomechanical dynamics. The outputs of the BiLSTM were further combined with the CNN-derived feature weights via a dot-product operation, allowing interaction between spatial importance and temporal evolution. Subsequently, a hybrid attention mechanism (HAM) was introduced to adaptively reweight the fused features, enhancing the model’s ability to focus on critical gait phases and key fatigue-related variables. This attention-based refinement improves feature discrimination under complex and noisy biomechanical conditions. Finally, the integrated features were processed through a linear layer and a Sigmoid activation function to introduce nonlinearity, followed by a Softmax function for output normalization and prediction.

The comparative results demonstrated that the proposed model consistently outperformed all baseline models, achieving lower RMSE and NRMSE values. This indicates that the hierarchical integration of spatial and temporal features, combined with attention-based weighting, provides a measurable advantage in predicting fatigue-related biomechanical indicators. These findings support the rationale for adopting the proposed architecture, particularly under limited sample conditions where efficient feature utilization is critical (Figures S6–S7).

**Fig. S6** Model prediction errors results. (A) Prediction errors of CD based on BiLSTM model. (B) Prediction errors of CD based on CNN-BiLSTM model. (C) Prediction errors of CD based on SSO–CNN-BiLSTM model. (D) Prediction errors of CD based on SSO–CNN-BiLSTM-HAM model. (E) Results of RMSE. (F) Results of NRMSE.

**Fig. S7** Model prediction errors results. (A) Prediction errors of PFF based on BiLSTM model. (B) Prediction errors of PFF based on CNN-BiLSTM model. (C) Prediction errors of PFF based on SSO–CNN-BiLSTM model. (D) Prediction errors of PFF based on SSO–CNN-BiLSTM-HAM model. (E) Results of RMSE. (F) Results of NRM

**Revisions: Page 34-35, Lines 698-709**

Third, although wearable IMU signals were used as the primary input features of the predictive framework, all wearable data in this study were collected under controlled laboratory conditions together with laboratory-grade biomechanical measurements. Therefore, the present framework should be interpreted as a laboratory-validated proof-of-concept rather than a fully field-deployed wearable monitoring system. In real-world running environments, additional variability may arise from factors such as sensor placement differences, environmental noise, and variations in running context, which may influence prediction stability. Future work will therefore focus on validating the framework using field-based wearable recordings and longitudinal monitoring to further enhance the ecological validity and practical applicability of the proposed fatigue prediction approach.

**Figure 1.** IMU Data

**Figure 2.** Impact Force Data

**Figure3.** Kinematic data

**2. Regarding biomechanical modeling, the authors used OpenSim for simulation. For Hallux Valgus, a deformity with significant skeletal structural changes, generic musculoskeletal models may not accurately reflect changes in joint moment arms and muscle lines of action. The authors should detail whether geometric scaling or adjustments specific to HV pathological features were performed on the model, and how these structural differences specifically affect the calculated internal loads. If no specific adjustments were made, the authors need to analyze in depth the potential errors introduced by model generalization in the discussion. Additionally, regarding the definition of "Cumulative Damage," which is currently based mainly on theoretical calculations using stress-life (S-N) curves, the authors should consider adding a discussion on the correlation between these theoretical calculations and actual clinical symptoms or radiographic evidence to enhance clinical translational significance.**

**Response:** Thank you for this constructive comment regarding the biomechanical modeling and the interpretation of the cumulative damage metric. We have clarified these aspects in the revised manuscript.

First, regarding the representation of HV in the musculoskeletal model, we acknowledge that HV involves structural changes in the forefoot that may influence joint geometry and muscle–tendon moment arms. During the experimental data collection, marker placement was adapted to account for the morphological characteristics of HV, and the marker configuration for HV participants differed slightly from that used for the healthy control group to better capture the altered first metatarsophalangeal alignment. These marker adjustments were incorporated during the kinematic reconstruction and scaling procedures within OpenSim. However, a fully subject-specific reconstruction of HV skeletal geometry was not implemented in the present study. The OpenSim model was scaled based on participant anthropometrics, while the underlying musculoskeletal geometry remained consistent across subjects. This decision was primarily made to maintain computational efficiency and model stability when integrating musculoskeletal simulation with large time-series datasets and deep learning prediction. Importantly, this approach does not imply that the HV and control groups were modeled identically, as the altered marker placement and kinematic patterns in HV runners still influenced the resulting joint kinematics and estimated internal loading patterns. We acknowledge that using a scaled generic model may introduce some approximation errors in representing pathological structural variations. To address this concern, we have expanded the discussion in the revised manuscript to explicitly acknowledge this limitation and clarify that future work will explore more personalized musculoskeletal reconstructions, which may further improve the representation of subject-specific skeletal alignment and tissue loading characteristics in HV populations.

Second, in the present study, CD is derived from stress–life (S–N) fatigue theory and is intended to represent a computational indicator of tissue-level mechanical fatigue accumulation under repetitive loading, rather than a direct measure of clinical injury or radiographic pathology. The purpose of this metric is to quantify the progressive mechanical burden experienced by biological tissues and to identify regions that may be more susceptible to fatigue-related injury. We acknowledge that the direct relationship between these theoretical fatigue indices and clinically observable outcomes has not yet been established within the scope of this study. This aspect has now been clarified in the revised manuscript. Future work will aim to integrate longitudinal clinical assessments and imaging data to further validate the translational relevance of these biomechanical fatigue indicators.

**Revisions: Page 34-35, Lines 694-709**

Second, the musculoskeletal simulations relied on idealized boundary conditions and assumed consistent ligament properties, which may not fully capture individual variability in tissue material behavior. In addition, although marker placement was adapted to account for hallux valgus morphology, the OpenSim model was based on a scaled generic template rather than a fully subject-specific reconstruction. Third, although wearable IMU signals were used as the primary input features of the predictive framework, all wearable data in this study were collected under controlled laboratory conditions together with laboratory-grade biomechanical measurements. Therefore, the present framework should be interpreted as a laboratory-validated proof-of-concept rather than a fully field-deployed wearable monitoring system. In real-world running environments, additional variability may arise from factors such as sensor placement differences, environmental noise, and variations in running context, which may influence prediction stability. Future work will therefore focus on validating the framework using field-based wearable recordings and longitudinal monitoring to further enhance the ecological validity and practical applicability of the proposed fatigue prediction approach.

**3. The authors focused on the accumulation of mechanical loads in the discussion section. To improve the depth of the paper, it is suggested that the authors add an in-depth discussion exploring the potential biological mechanisms of tissue injury caused by abnormal mechanical load transmission, as well as strategies for handling small sample data in the digital health field. For example, mechanosensitive ion channels (such as Piezo1 and TRPV4) play a key role in translating mechanical signals into biochemical signals (such as inflammation or tissue degeneration), which is closely related to the fatigue failure mechanism mentioned in the text (doi: 10.2174/0118761429317745241017114020). Meanwhile, long-term abnormal mechanical stress may lead to muscle atrophy or remodeling, which is mutually causal with the pathological process of foot deformities and is worth supplementing from a pathophysiological perspective (doi: 10.1016/j.bcp.2022.114954).**

**Response:** Thank you for this insightful suggestion. We appreciate the reviewer’s perspective on linking biomechanical loading with underlying biological mechanisms and on strategies for handling small-sample data in digital health research. Following this suggestion, we have learned from the referenced studies and incorporated these perspectives into the revised manuscript. Specifically, we have added a brief discussion to acknowledge the potential role of mechanobiological pathways (e.g., mechanosensitive ion channels and load-induced tissue remodeling) in interpreting the observed fatigue patterns and further refined the discussion on small-sample data considerations in the context of our modeling framework. These additions help to improve the depth and translational relevance of the manuscript while maintaining the primary focus on biomechanical modeling and fatigue prediction.

**Revisions: Page 32, Lines 639-641**

However, the PFF did not show significant differences. This lack of significance may suggest that PFF considers more complex factors beyond mechanical load or stress [54].

54.	Jia Z, Wang J, Li X, Yang Q, Han JJCMP. Repair effect of siRNA double silencing of the novel mechanically sensitive ion channels Piezo1 and TRPV4 on an osteoarthritis rat model. 2024;17(1):e18761429317745.

**Revisions: Page 33, Lines 674-677**

Moreover, CD and PFF values of the Achilles tendon and patellofemoral region remained elevated in the HV group, reflecting the cumulative mechanical burden imposed by altered gait patterns. Increased rearfoot eversion and hallux loading intensify ground reaction forces during stance, further accelerating fatigue accumulation. [58, 59].

59.	Wang W, Li M, Chen Z, Xu L, Chang M, Wang K, et al. Biogenesis and function of extracellular vesicles in pathophysiological processes of skeletal muscle atrophy. 2022;198:114954.

**Reviewer 4:**

**Major Comments
1. Multiple comparisons and SPM statistical control
The manuscript reports numerous significant time-window differences across multiple joints, variables (angles, moments, tissue forces), and outcome metrics using SPM and peak analyses. Although SPM controls for temporal smoothness within a single waveform, the manuscript does not clearly describe how family-wise error was handled across multiple joints and biomechanical variables. Given the large number of parallel statistical tests, the absence of an explicit multiple comparison strategy may raise concerns about inflated type I error.**

**Response:** Thank you for this important and thoughtful comment regarding multiple comparison control. We agree that conducting statistical analyses across multiple joints, biomechanical variables, and outcome metrics may increase the risk of type I error if not properly addressed. In the present study, different levels of statistical control were applied depending on the nature of the data.

For time-series variables, statistical parametric mapping (SPM) was employed. As recommended in the SPM framework, the significance threshold was determined using random field theory (RFT) correction at α = 0.05, which appropriately controls the family-wise error rate across the entire time continuum of each waveform, taking into account temporal smoothness. For discrete biomechanical parameters, independent-sample t-tests were conducted. When comparisons were performed across related variables within the same biomechanical domain, adjustments were applied to control the type I error rate, and this has now been clarified in the revised Methods section. In addition, the analyses were hypothesis-driven, focusing on predefined joints, anatomical regions, and fatigue-related indicators (CL, CD, and PFF), rather than performing fully exploratory testing across many unrelated variables. This targeted analysis strategy reduces the likelihood of spurious findings due to multiple comparisons.

Furthermore, it is worth noting that many of the observed differences were consistent across related biomechanical variables and across both time-series and discrete analyses, which provides additional support for the robustness of the findings rather than isolated statistically significant results. Nevertheless, we acknowledge that formal global correction across all variables and outcome measures was not implemented, and a residual risk of type I error may remain. This limitation has been explicitly acknowledged in the revised Discussion section. Future studies with larger sample sizes may benefit from adopting more stringent multiple comparison correction strategies (e.g., false discovery rate control or multivariate statistical approaches) to further enhance statistical rigor.

**Revisions: Page 22-23, Lines 431-445**

For time-series variables across the stance phase, statistical parametric mapping (SPM)-based independent-sample t-tests were conducted in MATLAB to examine temporal differences between the healthy and HV groups [18]. The significance threshold for SPM analyses was determined using random field theory correction at α = 0.05. For discrete biomechanical parameters—including anatomical indices, foot morphology measures, peak joint angles, joint moments, joint forces, range of motion (ROM), and fatigue indicators (CL, CD, and PFF)—independent-sample t-tests were performed in SPSS to compare the healthy and HV groups during running. To further investigate the associations between foot deformity and fatigue-related biomechanical indicators, Pearson correlation and linear regression analyses were conducted to examine the relationships between hallux valgus angle (HVA), Foot Posture Index (FPI), and the fatigue indices CL, CD, and PFF [17]. The significance level was set at α = 0.05. When multiple comparisons were performed across related variables, appropriate adjustments were applied to control the type I error rate.

**2. Correlation analyses without adjustment for confounders
Strong correlations are reported between HVA/FPI and CL/CD/PFF across tissues.
These correlations may be influenced by shared determinants such as running speed, cadence, body mass, BMI, training volume, or sex distribution. Without partial correlation or multivariable regression adjusting for potential confounders, the reported associations remain descriptive rather than mechanistically explanatory.**

**Response:** Thank you for this important comment regarding the interpretation of the correlation analyses. We agree that the reported associations between HVA/FPI and the fatigue-related indices (CL, CD, and PFF) may be influenced by potential confounding factors such as running speed, cadence, body mass, BMI, training background, or sex distribution. In the present study, Pearson correlation and linear regression analyses were primarily used to explore the strength and direction of associations between foot structural characteristics and fatigue-related biomechanical indicators. These analyses were designed to be hypothesis-driven, focusing specifically on the relationships between foot deformity (HVA, FPI) and tissue-level mechanical loading and fatigue metrics, rather than establishing causal or fully adjusted mechanistic models. In addition, experimental conditions such as running speed were controlled within a relatively narrow range during data collection, which may partially reduce variability from some external factors.

Nevertheless, we acknowledge that the current analyses do not explicitly adjust for all potential confounders, and therefore the reported associations should be interpreted as descriptive relationships rather than fully causal inferences. This point has now been clarified in the revised manuscript to avoid overinterpretation. Future studies with larger sample sizes will incorporate multivariable regression, partial correlation analyses, or mixed-effects modeling frameworks to better account for individual variability and potential confounding factors, thereby improving the mechanistic interpretability and robustness of the observed associations.

**Revisions: Page 30, Lines 594-599**

Specifically, we analyzed how structural characteristics of the feet in runners with HV are associated with pronation-related gait patterns, and how these variations may influence biomechanical characteristics during running. This work focuses on three key metrics to achieve this: CL, CD, and PFF. Changes in these indicators may reflect increased mechanical loading on tissues, particularly during prolonged running sessions.

**Revisions: Page 30, Lines 609-611**

Additionally, HV patients generally had a smaller knee joint ROM, which may influence running mechanics and potentially increase mechanical demands on the lower limb.

**Revisions: Page 33, Lines 661-663**

Runners with HV typically exhibit more pronounced foot pronation due to alterations in foot structure, which may influence gait mechanics and potentially contribute to increased lower-limb injury susceptibility.

**Revisions: Page 34, Lines 686-689**

In summary, this study integrates biomechanical modeling with deep learning–based fatigue prediction to reveal how HV-associated structural deformities amplify tissue loading, accelerate fatigue accumulation, and influence tissue loading patterns during running.

**3. Inconsistency between CL/CD and PFF findings
In several tissues (e.g., plantar fascia, patellofemoral region), CL and CD are significantly elevated while PFF is not. Since PFF is described as a fatigue failure probability derived from cumulative damage, a clearer explanation is needed regarding how PFF is computed and why statistical dissociation from CD may occur. Without methodological clarification of the fatigue model parameters and assumptions, interpretation of these discrepancies remains uncertain.**

**Response:** Thank you for this insightful and important comment regarding the relationship between CL, CD, and PFF.

We agree that the apparent discrepancy—where CL and CD show significant increases while PFF does not in certain tissues—requires further clarification. In the present study, these three metrics capture different stages and characteristics of the fatigue process. Specifically, cumulative load (CL) reflects the overall mechanical loading exposure, cumulative damage (CD) represents the progressive accumulation of tissue-level damage under repetitive loading, whereas the probability of fatigue failure (PFF) provides a probabilistic assessment of failure risk based on nonlinear fatigue behavior. Importantly, PFF is not a linear transformation of CD. It is derived from a probabilistic fatigue framework based on stress–life relationships and Weibull-type modeling, which incorporates nonlinear damage evolution, inter-individual variability, and threshold-dependent failure behavior. As a result, the mapping from CD to PFF is inherently nonlinear. In particular, sub-threshold increases in CD may lead to statistically significant differences in damage accumulation without necessarily producing a corresponding increase in failure probability. Therefore, the observed dissociation between CD and PFF in some tissues does not indicate inconsistency, but rather reflects the fact that PFF is less sensitive to moderate fluctuations in mechanical loading and more responsive to critical transitions in fatigue accumulation. This characteristic makes PFF a more conservative and stability-oriented indicator of fatigue failure risk, which may reduce the likelihood of overestimating injury risk based solely on load-based metrics.

We appreciate this insightful comment. We have clarified this distinction in our response to improve interpretability. From a methodological perspective, these findings highlight the complementary roles of CL, CD, and PFF: CL and CD reflect continuous loading and damage accumulation, whereas PFF captures the nonlinear transition from accumulation to failure risk. This distinction also explains why increases in CL and CD do not always result in significant changes in PFF and supports the role of PFF as a more robust indicator of fatigue failure risk.

**4. Extremely high R² values in deep learning models
Model 1 and Model 2 report R² values up to 0.94–0.97 for multiple fatigue indices.
Such high predictive performance in relatively small biomechanical datasets raises concerns about potential data leakage or overfitting. The manuscript does not clearly specify whether data partitioning was performed at the subject level or trial level. If multiple gait cycles from the same participant were included in both training and testing sets, performance metrics may be artificially inflated. Clear description of subject-level separation and independent validation is required.**

**Response:** Thank you for this important comment regarding the predictive performance of the proposed models.

First, we would like to clarify the data source and structure. The dataset used in this study was collected from 52 participants (26 with HV and 26 healthy controls), with each participant completing 10 successful running trials under controlled laboratory conditions. In addition to motion capture and force plate data, synchronized wearable IMU signals were also recorded, resulting in a relatively high-dimensional and information-rich dataset despite the moderate sample size.

To ensure the robustness of the modeling framework, we carefully designed the data processing and validation procedure. Specifically, data partitioning was performed at the subject level rather than the trial level, such that all trials from a given participant were included exclusively in either the training set or the testing set. This strategy avoids data leakage caused by shared subject-specific patterns across datasets and ensures that model performance reflects true generalization to unseen individuals.

Regarding model selection, the proposed SSO–CNN–BiLSTM–HAM architecture was not arbitrarily chosen. Prior to finalizing the model, we conducted comparative evaluations with several baseline models. We consistently observed that the proposed framework achieved the highest predictive accuracy and lowest prediction errors under the same data conditions. These results have been further clarified in the revised manuscript.

We acknowledge that the dataset size is relatively limited compared to large-scale machine learning studies. However, it is important to note that biomechanical data are typically highly structured and low-noise under controlled experimental conditions, and the combination of subject-specific modeling, time-series feature extraction, and deep learning enables effective learning of underlying patterns even with moderate sample sizes. Moreover, the consistency of model performance across multiple fatigue-related indices further supports the stability of the proposed framework.

**Revisions: Page 20, Lines 375-383**

The dataset consisted of 52 participants, including 26 individuals with hallux valgus and 26 healthy controls. All data were collected under controlled laboratory conditions during instrumented running experiments, where wearable IMU signals and laboratory-based biomechanical measurements were recorded simultaneously. Each participant completed ten running trials, resulting in a total of 520 biomechanical trials. For each trial, synchronized multichannel IMU signals were recorded from sensors placed on the foot, tibia, and femur. In parallel, laboratory biomechanical measurements were used to perform subject-specific musculoskeletal simulations to estimate tissue loading profiles.

**Revisions: Page 24, Lines 463-472**

Building upon this validated biomechanical framework, we further evaluated the effectiveness of the proposed deep learning architecture. Specifically, a series of baseline and progressively enhanced models were constructed, including BiLSTM, CNN–BiLSTM, SSO–CNN–BiLSTM, and SSO–CNN–BiLSTM–HAM. These models represent a hierarchical integration of temporal modeling, spatial–temporal feature extraction, parameter optimization, and attention-based feature refinement. Comparative results show a consistent reduction in prediction errors as model complexity increases, with the proposed SSO–CNN–BiLSTM–HAM achieving the lowest RMSE and NRMSE values. Detailed model configurations and extended comparative results are provided in **Supplementary Text 6 (Figures S6–S7).**

**Revisions: Supplementary Text 6, Lines 957-988**

To evaluate the necessity and effectiveness of the proposed architecture, we first conducted systematic comparisons with several baseline models, including standalone CNN, BiLSTM, and their simplified hybrid variants. These baseline models were designed to represent commonly used structures for handling spatial or temporal features in biomechanical time-series data. Performance was evaluated using RMSE and NRMSE to ensure both absolute and normalized error assessment.

Building upon these baseline configurations, the proposed SSO–CNN–BiLSTM–HAM framework was designed to progressively integrate spatial feature extraction, temporal sequence modeling, and adaptive feature weighting. Specifically, the CNN module was employed to capture local spatial patterns in the input signals and to learn feature-level importance weights. These weights reflect the relative contribution of different biomechanical variables. The extracted features were then passed to the BiLSTM module, which models bidirectional temporal dependencies across the gait cycle. Compared to unidirectional recurrent structures, BiLSTM enables a more comprehensive representation of time-dependent biomechanical dynamics. The outputs of the BiLSTM were further combined with the CNN-derived feature weights via a dot-product operation, allowing interaction between spatial importance and temporal evolution. Subsequently, a hybrid attention mechanism (HAM) was introduced to adaptively reweight the fused features, enhancing the model’s ability to focus on critical gait phases and key fatigue-related variables. This attention-based refinement improves feature discrimination under complex and noisy biomechanical conditions. Finally, the integrated features were processed through a linear layer and a Sigmoid activation function to introduce nonlinearity, followed by a Softmax function for output normalization and prediction.

The comparative results demonstrated that the proposed model consistently outperformed all baseline models, achieving lower RMSE and NRMSE values. This indicates that the hierarchical integration of spatial and temporal features, combined with attention-based weighting, provides a measurable advantage in predicting fatigue-related biomechanical indicators. These findings support the rationale for adopting the proposed architecture, particularly under limited sample conditions where efficient feature utilization is critical (Figures S6–S7).

**Fig. S6** Model prediction errors results. (A) Prediction errors of CD based on BiLSTM model. (B) Prediction errors of CD based on CNN-BiLSTM model. (C) Prediction errors of CD based on SSO–CNN-BiLSTM model. (D) Prediction errors of CD based on SSO–CNN-BiLSTM-HAM model. (E) Results of RMSE. (F) Results of NRMSE.

**Fig. S7** Model prediction errors results. (A) Prediction errors of PFF based on BiLSTM model. (B) Prediction errors of PFF based on CNN-BiLSTM model. (C) Prediction errors of PFF based on SSO–CNN-BiLSTM model. (D) Prediction errors of PFF based on SSO–CNN-BiLSTM-HAM model. (E) Results of RMSE. (F) Results of NRM

**5. Lack of external validation
Model performance appears to be evaluated within a single dataset.
Without external validation across independent cohorts, running speeds, or measurement systems, generalizability remains uncertain. The limitation section acknowledges lack of real-world validation, but the discussion of translational or scalable digital health implications appears stronger than the evidence currently supports.**

**Response:** Thank you for this important comment regarding external validation and generalizability. We agree that the current study is based on a single, laboratory-collected dataset and does not include validation across independent cohorts, running conditions, or measurement systems. Therefore, the generalizability of the proposed framework to broader populations and real-world environments remains to be further established.

In this work, our primary objective was to develop and validate a methodological framework that integrates biomechanical modeling with deep learning for fatigue prediction. Accordingly, the present study should be interpreted as a laboratory-based proof-of-concept, rather than a fully validated clinical or field-deployable system. Following this suggestion, we have refined the manuscript to ensure that the discussion of translational and digital health implications is more appropriately balanced. Specifically, statements related to real-world deployment have been moderated, and we now emphasize that further validation under diverse conditions is required, including different populations, running speeds, and sensor configurations.

In addition, to support transparency and facilitate future research, the dataset and relevant resources have been made publicly available. We hope that this will enable other researchers to further evaluate, reproduce, and extend the proposed framework using larger and more diverse datasets, thereby strengthening its generalizability and practical applicability. Future work will focus on expanding the dataset through multi-center data collection, as well as conducting external validation using independent cohorts and field-based wearable recordings, to further improve the robustness and scalability of the proposed approach.

**Revisions: Page 14, Lines 240-246**

In addition, five wearable inertial measurement units (IMUs; Xsens, Henderson, USA) were attached to the lower limbs to record multichannel acceleration signals. Sensors were placed on the foot and at the distal and proximal segments of the tibia and femur to capture segmental motion during running. The IMU system was synchronized with the laboratory biomechanical measurements to enable integrated analysis of wearable motion signals and laboratory-based biomechanical variables [37].

**Revisions: Page 34-35, Lines 698-709**

Third, although wearable IMU signals were used as the primary input features of the predictive framework, all wearable data in this study were collected under controlled laboratory conditions together with laboratory-grade biomechanical measurements. Therefore, the present framework should be interpreted as a laboratory-validated proof-of-concept rather than a fully field-deployed wearable monitoring system. In real-world running environments, additional variability may arise from factors such as sensor placement differences, environmental noise, and variations in running context, which may influence prediction stability. Future work will therefore focus on validating the framework using field-based wearable recordings and longitudinal monitoring to further enhance the ecological validity and practical applicability of the proposed fatigue prediction approach.

**6. Mechanistic interpretation exceeds presented analyses
The manuscript proposes a pathway from structural deviation (HV → pronation) to tissue-specific fatigue risk.
While group differences and correlations are demonstrated, formal mediation or pathway analysis linking HV severity, dynamic pronation measures, and fatigue indices is not shown. Therefore, mechanistic claims should be more cautiously framed as associative rather than causal.**

**Response:** Thank you for this insightful comment regarding the mechanistic interpretation of our findings. We agree that, although the study demonstrates significant group differences and associations among HV-related structural characteristics, foot pronation, and fatigue-related indices, formal mediation or pathway analyses were not performed to explicitly quantify causal relationships among these variables. Therefore, the proposed pathway linking structural deviation (HV), altered foot mechanics, and tissue-specific fatigue risk should be interpreted with appropriate caution.

In the present study, our intention was to provide a mechanistically informed interpretation based on the integration of biomechanical modeling, observed kinematic alterations, and fatigue-related indicators, rather than to establish a definitive causal pathway. To address this concern, we have revised the manuscript to ensure that the relevant descriptions are framed more appropriately as associative relationships, avoiding overstatement of causality. Importantly, the observed associations between HV severity, pronation-related measures (e.g., FPI), and fatigue indices were consistent across multiple tissues and analytical approaches, which supports the biomechanical plausibility of the proposed relationships. However, we acknowledge that these findings do not constitute formal evidence of mediation or causal pathways.

Future work will incorporate mediation analysis, multivariate modeling, and longitudinal study designs to more rigorously evaluate the causal relationships and interaction pathways between structural deformity, dynamic foot mechanics, and fatigue progression.

**Revisions: Page 30, Lines 594-599**

Specifically, we analyzed how structural characteristics of the feet in runners with HV are associated with pronation-related gait patterns, and how these variations may influence biomechanical characteristics during running. This work focuses on three key metrics to achieve this: CL, CD, and PFF. Changes in these indicators may reflect increased mechanical loading on tissues, particularly during prolonged running sessions.

**Revisions: Page 30, Lines 609-611**

Additionally, HV patients generally had a smaller knee joint ROM, which may influence running mechanics and potentially increase mechanical demands on the lower limb.

**Revisions: Page 33, Lines 661-663**

Runners with HV typically exhibit more pronounced foot pronation due to alterations in foot structure, which may influence gait mechanics and potentially contribute to increased lower-limb injury susceptibility.

**Revisions: Page 34, Lines 686-689**

In summary, this study integrates biomechanical modeling with deep learning–based fatigue prediction to reveal how HV-associated structural deformities amplify tissue loading, accelerate fatigue accumulation, and influence tissue loading patterns during running.

**7. DFIS validation requires quantitative detail
The manuscript states that model validation using DFIS showed strong agreement.
The number of participants validated, quantitative agreement metrics (e.g., RMSE, ICC), and validation scope (joint kinematics vs tissue forces vs fatigue indices) are not clearly detailed in the presented text. Without quantitative reporting, the strength of model validation cannot be fully assessed.**

**Response:** Thank you for this important and constructive comment. We agree that quantitative validation metrics are essential to fully assess the reliability of the proposed model. In the revised manuscript, we have clarified the scope, sample basis, and quantitative outcomes of the DFIS-based validation. Specifically, dual fluoroscopic imaging system (DFIS) data were used to validate joint-level kinematics of the musculoskeletal model, focusing on the three-dimensional tibiotalar displacements, including the Medial and Lateral (ML), Posterior and Anterior (PA), Distal and Proximal (DP) directions.

Quantitative agreement between the simulated results and DFIS measurements was evaluated using the root mean square error (RMSE) and the intraclass correlation coefficient (ICC, two-way random effects, absolute agreement). The results demonstrated excellent consistency across all directions:

DP displacement: RMSE = 0.0547, ICC = 0.985

PA displacement: RMSE = 0.1065, ICC = 0.9653

ML displacement: RMSE = 0.0742, ICC = 0.9786

These results indicate excellent agreement between the model predictions and DFIS measurements, supporting the validity of the musculoskeletal model in reproducing subject-specific joint kinematics during running. We have also clarified in the revised manuscript that the scope of DFIS validation is inherently limited to skeletal kinematics. Internal variables, such as muscle forces and fatigue-related indices (CL, CD, and PFF), are derived from model-based computations and cannot be directly validated using DFIS measurements. Instead, their physiological plausibility is supported through the integration of validated musculoskeletal modeling frameworks, experimental inputs, and consistency with established biomechanical principles.

**Revisions: Supplementary material Text 5**

This work collected the foot-ankle fluoroscopic image data of the subjects in the running state by the high-speed dual fluoroscopic imaging system (DFIS) (Ti-WISH-Ⅱ, Ti-Motion ltd., Shanghai City, CN), and compared the calculated tibiotalar displacement results with the musculoskeletal modeling simulations results to verify the validity of foot-ankle model with DCTs (AT and PFS). **Fig. 1B** illustrates the procedure and outcomes of foot-ankle displacement calculation based on DFIS. By generating high voltage through a high voltage generator, the DFIS can accelerate the free electrons inside the X-ray tube towards the target surface to emit X-rays. Afterward, based on the principle that the X-rays emitted pass through different tissues and organs of the human body with different levels of attenuation, the X-rays that pass through the human body and carry enough information are projected onto a flat-panel detector. The computer processes the analog signals into digital signals through photoelectric conversion and analog-to-digital conversion, thereby providing reliable gray-scale medical image data for clinical diagnosis. The parameters setting of DFIS are as follows: source image distance 1350 mm, device voltage 60 kV, device current 500 mA, exposure time 3 seconds, laser wavelength 650 nm, dose range for the automatic exposure control 45 μGy, flat panel detector size 3072×3072 pixel, X-ray area 427×427 mm, nominal voltage of the ray tube 150 kV, laser power 3 mW, and sector angle 90°.

**8. Sample size and model complexity balance
The study includes 26 HV and 26 controls, yet employs hierarchical deep learning architectures.
Given the relatively small subject number, justification for model complexity, overfitting prevention strategy, and effective sample size definition (subjects vs gait cycles) should be clearly described to ensure robustness.**

**Response:** Thank you for this important comment regarding the balance between sample size and model complexity. We acknowledge that the number of participants in this study (26 HV and 26 controls) is relatively modest. However, it is important to clarify that each participant completed multiple successful running trials, and time-series data were collected across the full stance phase, resulting in a high-dimensional dataset with multiple gait cycles per subject. Therefore, while the number of subjects is limited, the effective sample size in terms of temporal and biomechanical features is substantially larger. To ensure that the model evaluation reflects true generalization rather than subject-specific memorization, data partitioning was performed at the subject level, such that all trials from a given participant were included exclusively in either the training or testing set. This approach prevents data leakage across sets and provides a more rigorous assessment of model performance.

Regarding model complexity, the proposed SSO–CNN–BiLSTM–HAM architecture was selected based on comparative evaluation with several baseline models, where it consistently achieved the highest predictive accuracy and lowest prediction errors under the same data conditions. These comparisons have now been clarified to better justify the choice of model architecture. The hierarchical design of the model enables it to capture both spatial–structural features (via CNN) and temporal dependencies (via BiLSTM), which are essential for representing biomechanical time-series data.

In addition, several strategies were employed to reduce the risk of overfitting, including subject-level data separation, normalization of input features, and consistent performance across multiple fatigue-related outputs. Despite these precautions, we acknowledge that the relatively small number of subjects remains a limitation, and this has been explicitly discussed in the revised manuscript. Future work will focus on expanding the dataset and exploring validation strategies more suitable for small-sample scenarios, such as cross-dataset validation and model simplification, to further enhance robustness and generalizability.

**Revisions: Page 20, Lines 375-383**

The dataset consisted of 52 participants, including 26 individuals with hallux valgus and 26 healthy controls. All data were collected under controlled laboratory conditions during instrumented running experiments, where wearable IMU signals and laboratory-based biomechanical measurements were recorded simultaneously. Each participant completed ten running trials, resulting in a total of 520 biomechanical trials. For each trial, synchronized multichannel IMU signals were recorded from sensors placed on the foot, tibia, and femur. In parallel, laboratory biomechanical measurements were used to perform subject-specific musculoskeletal simulations to estimate tissue loading profiles.

**Figure 1.** IMU Data

**Figure 2.** Impact Force Data

**Figure3.** Kinematic data

**Revisions: Page 24, Lines 463-472**

Building upon this validated biomechanical framework, we further evaluated the effectiveness of the proposed deep learning architecture. Specifically, a series of baseline and progressively enhanced models were constructed, including BiLSTM, CNN–BiLSTM, SSO–CNN–BiLSTM, and SSO–CNN–BiLSTM–HAM. These models represent a hierarchical integration of temporal modeling, spatial–temporal feature extraction, parameter optimization, and attention-based feature refinement. Comparative results show a consistent reduction in prediction errors as model complexity increases, with the proposed SSO–CNN–BiLSTM–HAM achieving the lowest RMSE and NRMSE values. Detailed model configurations and extended comparative results are provided in **Supplementary Text 6 (Figures S6–S7).**

**Revisions: Supplementary Text 6, Lines 957-988**

To evaluate the necessity and effectiveness of the proposed architecture, we first conducted systematic comparisons with several baseline models, including standalone CNN, BiLSTM, and their simplified hybrid variants. These baseline models were designed to represent commonly used structures for handling spatial or temporal features in biomechanical time-series data. Performance was evaluated using RMSE and NRMSE to ensure both absolute and normalized error assessment.

Building upon these baseline configurations, the proposed SSO–CNN–BiLSTM–HAM framework was designed to progressively integrate spatial feature extraction, temporal sequence modeling, and adaptive feature weighting. Specifically, the CNN module was employed to capture local spatial patterns in the input signals and to learn feature-level importance weights. These weights reflect the relative contribution of different biomechanical variables. The extracted features were then passed to the BiLSTM module, which models bidirectional temporal dependencies across the gait cycle. Compared to unidirectional recurrent structures, BiLSTM enables a more comprehensive representation of time-dependent biomechanical dynamics. The outputs of the BiLSTM were further combined with the CNN-derived feature weights via a dot-product operation, allowing interaction between spatial importance and temporal evolution. Subsequently, a hybrid attention mechanism (HAM) was introduced to adaptively reweight the fused features, enhancing the model’s ability to focus on critical gait phases and key fatigue-related variables. This attention-based refinement improves feature discrimination under complex and noisy biomechanical conditions. Finally, the integrated features were processed through a linear layer and a Sigmoid activation function to introduce nonlinearity, followed by a Softmax function for output normalization and prediction.

The comparative results demonstrated that the proposed model consistently outperformed all baseline models, achieving lower RMSE and NRMSE values. This indicates that the hierarchical integration of spatial and temporal features, combined with attention-based weighting, provides a measurable advantage in predicting fatigue-related biomechanical indicators. These findings support the rationale for adopting the proposed architecture, particularly under limited sample conditions where efficient feature utilization is critical (Figures S6–S7).

**Fig. S6** Model prediction errors results. (A) Prediction errors of CD based on BiLSTM model. (B) Prediction errors of CD based on CNN-BiLSTM model. (C) Prediction errors of CD based on SSO–CNN-BiLSTM model. (D) Prediction errors of CD based on SSO–CNN-BiLSTM-HAM model. (E) Results of RMSE. (F) Results of NRMSE.

**Fig. S7** Model prediction errors results. (A) Prediction errors of PFF based on BiLSTM model. (B) Prediction errors of PFF based on CNN-BiLSTM model. (C) Prediction errors of PFF based on SSO–CNN-BiLSTM model. (D) Prediction errors of PFF based on SSO–CNN-BiLSTM-HAM model. (E) Results of RMSE. (F) Results of NRM

**9. Interpretation of sex-specific findings
Sex differences are described in subgroup analyses.
Given limited subgroup sizes, statistical power for sex-specific inference may be constrained. The manuscript should clarify whether analyses were pre-specified and whether interaction testing was performed.**

**Response:** Thank you for this comment regarding the interpretation of sex-specific findings. We would like to clarify that the present study did not aim to investigate sex-specific effects, and no formal analyses comparing male and female participants were performed. Sex was included only as a basic demographic characteristic to describe the study population and to ensure appropriate reporting of participant information. The primary objective of this study was to examine differences between runners with hallux valgus and healthy controls in terms of biomechanical loading and fatigue-related indices. Accordingly, all statistical analyses were designed around group-level comparisons and sex was not incorporated as an independent factor or interaction term in the analytical framework.

We acknowledge that, given the sample size, any subgroup analysis based on sex would be underpowered and may lead to unreliable interpretations. To avoid potential misunderstanding, we will further clarify in the manuscript that sex was included for descriptive purposes only and was not analyzed as a factor influencing the outcomes. Future studies with larger and more balanced cohorts may allow for a more rigorous investigation of sex-specific effects using appropriately powered statistical designs.

**10. Overextension of clinical translation claims
The manuscript suggests inclusion of structural markers into routine precision injury prevention frameworks.
Given the absence of prospective injury outcome data and lack of longitudinal validation, recommendations for clinical or remote monitoring implementation should be more conservatively stated.
The manuscript reports numerous significant time-window differences across multiple joints, variables (angles, moments, tissue forces), and outcome metrics using SPM and peak analyses.
Although SPM controls for temporal smoothness within a single waveform, the manuscript does not clearly describe how family-wise error was handled across multiple joints and biomechanical variables. Given the large number of parallel statistical tests, the absence of an explicit multiple comparison strategy may raise concerns about inflated type I error.**

**Response:** Thank you for these insightful comments regarding both the clinical interpretation and the statistical rigor of the study.

**(1) Clinical translation and interpretation**

We appreciate the reviewer’s concern that some statements in the original manuscript may have conveyed an overly strong implication of immediate clinical applicability. We would like to clarify that the primary objective of this study is to develop a mechanism-informed and data-driven framework for characterizing fatigue-related biomechanical patterns in runners with hallux valgus, rather than to propose a fully deployable clinical or remote monitoring solution.

In response to this comment, we have carefully revised and moderated the relevant statements throughout the manuscript, particularly in the Discussion and Conclusion sections. The framework is now more appropriately described as a laboratory-validated, proof-of-concept approach, with potential for future application in precision injury prevention. We have also explicitly emphasized that prospective validation, longitudinal injury tracking, and cross-environment testing are necessary before clinical or field implementation can be considered.

Importantly, we intend to highlight the potential value of integrating structural indicators with biomechanical fatigue modeling, rather than to recommend immediate incorporation into routine clinical workflows. The revised manuscript now reflects this more cautious and translationally appropriate interpretation.

**(2) Multiple comparisons and statistical control**

We thank the reviewer for raising this important point regarding multiple comparisons. For time-series analyses, statistical parametric mapping (SPM) was employed with random field theory–based correction (α = 0.05), which provides control of the family-wise error rate within each continuous waveform by accounting for temporal smoothness and data correlation structure. For discrete biomechanical variables, independent-sample t-tests were conducted. When multiple comparisons were performed across related variables, appropriate adjustments were applied to control the type I error rate, and this has now been more explicitly clarified in the revised Methods section.

We acknowledge that, given the number of joints, variables, and outcome measures analyzed, residual concerns regarding multiple testing may arise. Therefore, in addition to clarifying the statistical procedures, we have also reframed the interpretation of the results to emphasize consistent patterns across tissues and metrics, rather than relying on isolated significant findings.

**Revisions: Page 30, Lines 594-599**

Specifically, we analyzed how structural characteristics of the feet in runners with HV are associated with pronation-related gait patterns, and how these variations may influence biomechanical characteristics during running. This work focuses on three key metrics to achieve this: CL, CD, and PFF. Changes in these indicators may reflect increased mechanical loading on tissues, particularly during prolonged running sessions.

**Revisions: Page 30, Lines 609-611**

Additionally, HV patients generally had a smaller knee joint ROM, which may influence running mechanics and potentially increase mechanical demands on the lower limb.

**Revisions: Page 33, Lines 661-663**

Runners with HV typically exhibit more pronounced foot pronation due to alterations in foot structure, which may influence gait mechanics and potentially contribute to increased lower-limb injury susceptibility.

**Revisions: Page 34, Lines 686-689**

In summary, this study integrates biomechanical modeling with deep learning–based fatigue prediction to reveal how HV-associated structural deformities amplify tissue loading, accelerate fatigue accumulation, and influence tissue loading patterns during running.

**Revisions: Page 34-35, Lines 698-709**

Third, although wearable IMU signals were used as the primary input features of the predictive framework, all wearable data in this study were collected under controlled laboratory conditions together with laboratory-grade biomechanical measurements. Therefore, the present framework should be interpreted as a laboratory-validated proof-of-concept rather than a fully field-deployed wearable monitoring system. In real-world running environments, additional variability may arise from factors such as sensor placement differences, environmental noise, and variations in running context, which may influence prediction stability. Future work will therefore focus on validating the framework using field-based wearable recordings and longitudinal monitoring to further enhance the ecological validity and practical applicability of the proposed fatigue prediction approach.

**Revisions: Page 22-23, Lines 431-445**

For time-series variables across the stance phase, statistical parametric mapping (SPM)-based independent-sample t-tests were conducted in MATLAB to examine temporal differences between the healthy and HV groups [18]. The significance threshold for SPM analyses was determined using random field theory correction at α = 0.05. For discrete biomechanical parameters—including anatomical indices, foot morphology measures, peak joint angles, joint moments, joint forces, range of motion (ROM), and fatigue indicators (CL, CD, and PFF)—independent-sample t-tests were performed in SPSS to compare the healthy and HV groups during running. To further investigate the associations between foot deformity and fatigue-related biomechanical indicators, Pearson correlation and linear regression analyses were conducted to examine the relationships between hallux valgus angle (HVA), Foot Posture Index (FPI), and the fatigue indices CL, CD, and PFF [17]. The significance level was set at α = 0.05. When multiple comparisons were performed across related variables, appropriate adjustments were applied to control the type I error rate.

**Minor Comments
11. Clarification of fatigue modeling parameters
The manuscript would benefit from explicit presentation of material constants, fatigue thresholds, and assumptions underlying the cumulative damage model to enhance reproducibility.**

**Response:** Thank you for this valuable comment regarding the clarity and reproducibility of the fatigue modeling framework. We would like to clarify that the key material constants, fatigue-related parameters, and modeling assumptions are fully provided in the **Supplementary Materials (Supplementary Texts 3 and 4)**. These include: Material parameters of ligament dense connective tissues, such as the elastic modulus (*K* = 70 MPa), viscosity coefficient (*η* = 20 MPa·s), and Weibull distribution parameters (*α* = 4.5, *β* = 0.3), which define the nonlinear viscoelastic behavior of the tissues; Fatigue model parameters, including the weighting factor (*b* = 9.3 for soft tissues and 7 for bone), Weibull modulus (*mW* = 6.7), and the reference strain scaling used in the probability of fatigue failure (*PFF*) formulation; Core modeling assumptions, such as nonlinear strain–time viscoelasticity (NLSTV), collagen fiber recruitment and probabilistic uncrimping behavior, and the integration of fatigue and creep effects within the cumulative damage framework.

All parameters were derived from previously validated experimental and modeling studies, ensuring their physiological relevance and consistency with established literature. We acknowledge that, in the original presentation, these parameters were primarily embedded within the mathematical formulations, which may reduce their immediate visibility. To improve clarity, we have revised the manuscript to more explicitly indicate where these parameters are defined and to guide readers to the relevant Supplementary sections.

**12. Reporting of uncertainty
Confidence intervals for effect sizes and predictive metrics are not consistently reported. Including interval estimates would strengthen statistical transparency.**

**Response:** Thank you for this important comment regarding statistical transparency.

We acknowledge that confidence intervals were not consistently reported for all effect sizes and predictive metrics in the original manuscript. In the present study, statistical inference was primarily based on SPM analyses with random field theory correction for time-series data and hypothesis testing (p-values) for discrete variables, together with model performance metrics such as R², MAE, and RMSE.

We agree that reporting interval estimates would further enhance the interpretability and transparency of the results. Accordingly, we have clarified the statistical reporting and will consider incorporating confidence interval estimation more systematically in future work.

**13. Description of data normalization**

**Details regarding gait speed normalization, stride normalization, and signal preprocessing should be clearly summarized to facilitate replication.**

**Response:** Thank you for this helpful comment regarding the clarity of data normalization procedures. We would like to clarify that gait speed in this study was controlled using a photoelectric timing system, which ensured that all participants ran within a predefined and consistent speed range during data collection. This approach minimized inter-subject variability associated with running velocity and improved the comparability of biomechanical signals.

In addition, all gait data were time-normalized to the stance phase (0–100%) to enable consistent comparison across participants and trials. Key biomechanical variables, including joint kinematics, kinetics, and tissue-level loading signals, were resampled to a standardized number of data points over the stance phase. Regarding signal preprocessing, marker trajectories and ground reaction force data were processed using standard filtering and inverse dynamics procedures within the motion capture and musculoskeletal modeling pipeline. IMU-derived signals were synchronized with laboratory-based measurements to ensure temporal consistency.

We acknowledge that these steps were not sufficiently summarized in the original manuscript. Accordingly, we have clarified the relevant descriptions in the revised Methods section to improve reproducibility.

**Revisions: Page 14, Lines 252-257**

To estimate per-kilometer cumulative load (CL), participants first ran 1 km continuously at 12 km/h on a Zebris treadmill (Zebris Medical GmbH, Germany) before overground testing. Two AMTI force plates were embedded mid-runway (20 m) with infrared timing gates (4 m apart) to control speed (3.33 ± 10% m/s). Each participant completed ten successful trials, ensuring right-foot and left-foot steps on the first and second force plates, respectively.

**Revisions: Page 15, Lines 267-269**

To minimize signal noise, a zero-phase fourth-order Butterworth filter was employed with cut-off frequencies of 10 Hz for kinematic and 20 Hz for kinetic signals [17, 18].

**14. Clarification of effective sample size
If multiple gait cycles per participant were used, the manuscript should explicitly state how statistical independence was ensured**

**Response:** Thank you for this important comment. We would like to clarify that, although multiple gait cycles were collected for each participant, all statistical analyses were conducted at the participant level. Specifically, multiple gait cycles were used to improve signal reliability, and representative measures were obtained for each participant prior to group-level statistical comparisons. Therefore, each participant contributed a single set of observations, ensuring statistical independence.

For the deep learning analysis, multiple gait cycles were used as time-series inputs to enhance model robustness. Importantly, data partitioning was performed at the subject level, such that all data from a given participant were included exclusively in either the training or testing set. This strategy effectively prevents data leakage and avoids inflation of model performance.

**15. Manuscript formatting does not comply with journal requirements
The current version does not appear to follow the target journal’s formatting guidelines (structure, figure presentation, referencing style, and layout). Proper adherence to journal formatting standards is required prior to publication consideration.**

**Response:** Thank you for this comment. We have carefully reviewed the manuscript and made comprehensive revisions to ensure full compliance with the target journal’s formatting requirements. Specifically, the manuscript structure, figure presentation, referencing style, and overall layout have been standardized in accordance with the journal guidelines. All formatting-related issues have been addressed, and we believe the revised version now meets the required submission standards.
