# Supplementary material for: Structure-aware fatigue modeling in foot deformities: A digital health framework for tissue-specific running injury risk prediction using multi-modal data
Source: PLOS Digit Health. 2026 Jul 10;5(7):e0001537. doi: 10.1371/journal.pdig.0001537 (PMC13354006; doi:10.1371/journal.pdig.0001537)
Supplement: Attachment — Submitted filename: Response to Editor and Reviewers R1.docx [file pdig.0001537.s007.docx]

**Response to the Reviewers' Comments**

**Manuscript Number: PDIG-D-25-01162R1**

**Dear Editors and Reviewers:**

Thank you very much for your constructive comments and time spent analyzing our Manuscript entitled "**Structure-Aware Fatigue Modeling in Foot Deformities: A Digital Health Framework for Tissue-Specific Running Injury Risk Prediction Using Multi-Modal Data**". Those comments are all valuable and very helpful for revising and improving our paper, as well as providing important guiding significance to our research. We have studied the comments carefully and made corrections which we hope to meet with approval. Revised portions are highlighted in red on the Manuscript. Here below is our description of the revision according to the reviewers' comments.

**Response to Reviewer 1: Pages 1-23.**

**Reviewer 1:**

**Thank you for the substantive revisions submitted in response to the first-round review. The authors have made meaningful progress across several dimensions. Subject-level data partitioning is now explicitly described in the Methods, directly addressing the information leakage concern raised at R1. The DFIS-based musculoskeletal model validation has been strengthened with quantitative agreement metrics (ICC 0.965–0.985 across tibiotalar displacement planes). Baseline model ablation comparisons have been added to the supplementary materials. The laboratory context of the IMU data collection is now correctly characterized throughout the Methods and Limitations sections. Causal language in the Discussion and Results has been appropriately moderated. The Associate Editor's formatting requirements — including the ethics statement, financial disclosure, figure filing, and data availability statement — have all been addressed. These revisions represent a genuine improvement in methodological transparency and reporting quality.**

**However, several concerns remain that must be resolved before the manuscript can be accepted for publication. Three issues are classified as mandatory corrections. Three additional concerns require author attention at the level of either targeted analysis or explicit inferential restriction.**

**Response:** Thank you for the reviewer’s careful evaluation and for recognizing the conceptual contribution of this work in integrating subject-specific musculoskeletal modeling, tissue-level fatigue analysis, and wearable-driven predictive modeling. We appreciate the reviewer’s constructive concerns regarding validation rigor, model complexity, reproducibility, and the framing of translational claims. In response, we have substantially revised the manuscript to strengthen the methodological transparency and robustness of the predictive framework. Specifically, we clarified the data partitioning strategy with strict participant-level separation, expanded the reporting of predictive performance with additional evaluation metrics and uncertainty estimates, included baseline model comparisons and ablation analyses, and moderated statements regarding real-world wearable deployment. In addition, we improved the reproducibility description by providing more detailed information on data processing and model training procedures. These revisions aim to enhance the reliability and interpretability of the proposed framework while aligning the manuscript more closely with the standards expected for reproducible digital health research.

**Mandatory Corrections**

**1.** **Multiple comparison correction for discrete biomechanical variables — method not specified**

**The revised Methods section states that "when multiple comparisons were performed across related variables, appropriate adjustments were applied to control the type I error rate." This statement is procedurally insufficient for a journal committed to reproducible research. The manuscript reports independent-sample t-tests across multiple discrete outcome domains simultaneously: anatomical indices (HVA, IMA, FPI), joint kinematics across multiple planes and joints, joint kinetics, range of motion variables, and fatigue indices (CL, CD, PFF) at four tissue sites. The reader cannot assess whether the type I error rate was adequately controlled without knowing which correction procedure was applied, which tests were grouped into a comparison family, how many tests were included in each family, and what the corrected alpha threshold or adjusted p-values were for each domain. Please specify the correction method (e.g., Bonferroni, Holm-Bonferroni, Benjamini-Hochberg FDR) in the Statistical Analysis subsection of the Methods, state the number of comparisons in each family, and report the corrected significance thresholds. This information should also be reflected in the Results tables where applicable.**

**Response:** Thank you for this insightful comment regarding the transparency and reproducibility of our statistical procedures. We agree that the previous description of multiple comparison correction lacked sufficient detail to allow full assessment of type I error control.

In the revised manuscript, we have substantially clarified the multiple comparison correction strategy in the Statistical Analysis subsection. Specifically, the Benjamini–Hochberg false discovery rate (FDR) procedure was applied to control for the increased risk of type I error arising from multiple hypothesis testing. This approach was selected as it provides an appropriate balance between error control and statistical power in the context of high-dimensional biomechanical data. To ensure methodological transparency, all variables were grouped into predefined comparison families based on their biomechanical domains: (1) anatomical indices (HVA, IMA, FPI; n = 3), (2) joint kinematics and kinetics variables (n = 20), and (3) fatigue-related indices (CL, CD, and PFF across four tissue sites; n = 12). Independent sample t-tests were conducted for each variable, and FDR correction was applied. Statistical significance was determined using an adjusted threshold of q < 0.05.

In addition, the Results section and corresponding tables have been revised to reflect these adjustments. Specifically, FDR-adjusted q-values are now reported alongside the original p-values, and statistical significance after correction has been clearly indicated where applicable. This allows readers to directly assess the impact of multiple comparison correction across different domains. We believe these revisions fully address the reviewer’s concerns and ensure that the statistical analysis is transparent, reproducible, and consistent with current best practices in biomechanical and digital health research.

**Revisions: Page 23 Lines 445-451**

When multiple comparisons were performed across related variables, the Benjamini–Hochberg false discovery rate (FDR) procedure was applied to correct for multiple comparisons. To this purpose, variables were grouped into three domains based on their biomechanical characteristics: anatomical indices (HVA, IMA, FPI; n = 3), joint kinematics and kinetics (n = 20), and fatigue-failure related indices (CL, CD, and PFF across four tissues; n = 12) for the correction. All comparisons within each domain were adjusted independently using the FDR procedure, with statistical significance defined as q < 0.05.

**2. Model complexity relative to sample size**

**The proposed architecture combines multiple high capacity components and an optimization routine, yet the cohort size is limited. This raises substantial concern regarding overfitting and model stability. Performance is reported without uncertainty measures, further limiting interpretability.**

**Response:** We thank the reviewer for this valuable comment regarding the potential influence of confounding variables on the reported correlation analyses. We agree that factors such as body mass, running speed, and training volume may affect fatigue-related indices and could potentially influence the observed associations with structural variables.

In the present study, correlation analyses were primarily intended as an exploratory step to examine the relationships between foot structure (HVA and FPI) and tissue-specific fatigue metrics within the broader biomechanical modeling framework. We acknowledge that these analyses were conducted without adjustment for potential confounders, which may limit the interpretability of the findings. Following the reviewer’s suggestion, we have revised the manuscript to more clearly and explicitly reflect this limitation. Specifically, all correlation results are now described as unadjusted bivariate associations, and we have clarified in both the Results and Discussion sections. In addition, we have carefully revised the relevant text throughout the manuscript to avoid causal or mechanistic interpretations based solely on these correlations. The language has been adjusted to reflect associative relationships, and the corresponding limitation has been clearly acknowledged.

We believe that these revisions provide a more appropriate and transparent interpretation of the results while maintaining the exploratory value of the analysis. Future work incorporating multivariable or adjusted analytical approaches will be important to further validate these findings.

**Revisions: Page 32 Lines 635-637**

This progressive accumulation of load, combined with greater strain, may be associated with an increased likelihood of fatigue-related tissue responses.

**Revisions: Page 32 Lines 658-662**

This improvement suggests that fatigue-related patterns in HV runners are not solely captured by mechanical loading variables, but are also associated with structural characteristics such as foot posture. These findings indicate that incorporating structural parameters may enhance the representation of individual variability in fatigue-related responses [23, 57].

**Revisions: Page 34 Lines 694-699**

In summary, this study integrates biomechanical modeling with deep learning–based fatigue prediction to characterize associations between HV-related structural deformities and tissue-specific loading and fatigue patterns during running. By combining CL, CD, and PFF with FPI- and HVA-driven adaptive optimization, the framework provides a multiscale perspective linking foot deformity, mechanical stress, and fatigue-related progression.

**3. Reference list — reference 55 absent**

**The reference list in the revised manuscript jumps from reference 54 (Wren et al., Clinical Biomechanics, 2001) to reference 56 (Jia et al., Current Molecular Pharmacology, 2024). Reference 55 is absent. Please audit the full reference list against all in-text citations, identify the intended reference 55, restore it to the list, and verify that all subsequent citation numbers are correct. A note in the cover letter specifying the nature of the error would assist the editorial office.**

**Response:** We sincerely apologize for this oversight in the reference list and thank the reviewer for carefully identifying this issue. In the revised manuscript, we have thoroughly audited the entire reference list against all in-text citations. The missing Reference 55 has now been correctly identified and restored, and all subsequent references have been carefully renumbered to ensure consistency throughout the manuscript. In addition, we have conducted a comprehensive cross-check of all citations and references to prevent similar inconsistencies. We appreciate the reviewer’s attention to detail, which has helped us improve the accuracy and overall quality of the manuscript.

**Revisions: Page 44 Lines 961-963**

**55.**	Omar K, Mohamed A, Essa M, et al. The influence of hallux valgus severity on postural stability and risk of fall: a cross-sectional study design. BMC Musculoskeletal Disorders, 2026, 27(1): 29. <https://doi.org/10.1186/s12891-025-09357-6>

**Additional Required Revisions
4. Author summary — real-world monitoring language
The author summary states that the proposed framework enables "simple, non-invasive, and continuous monitoring outside specialized labs" and provides "individualized, real-time fatigue assessment" with potential for "large-scale screening in community and clinical settings." These claims are inconsistent with the characterization now present throughout the revised manuscript body, where the study is correctly described as a laboratory-validated proof-of-concept in which IMU signals were collected under controlled laboratory conditions synchronized with motion-capture instrumentation. The author summary is published alongside the article and is read by non-specialist audiences. Please revise it to accurately reflect the laboratory context of the data and the proof-of-concept status of the framework.**

**Response:** We sincerely thank the reviewer for this valuable comment regarding the Author Summary. We fully agree that the original wording may have overemphasized real-world applicability and real-time monitoring, which could be misleading given the current study design. In response, we have carefully revised the Author Summary to ensure consistency with the main manuscript. Specifically, we have removed statements implying real-world deployment, continuous monitoring outside laboratory settings, and real-time fatigue assessment. The revised version now clearly reflects that the proposed framework is a laboratory-validated proof-of-concept, developed and tested under controlled experimental conditions using synchronized wearable and motion-capture data. We have also revised the language to emphasize feasibility and potential future application rather than immediate clinical or community deployment. We appreciate the reviewer’s insightful suggestion, which has helped improve the accuracy, clarity, and appropriate positioning of the study for a broader audience.

**Revisions: Page 4-5 Lines 56-75**

Foot deformities are common in the general population, and hallux valgus is one of the most prevalent conditions. Although often considered a mild structural alteration, hallux valgus can influence the leg loading patterns during daily movement and sports such as running. Over time, these altered loading patterns may contribute to increased mechanical demand on key musculoskeletal tissues, such as the Achilles tendon and plantar fascia, potentially elevating the risk of overuse-related injury. However, because the biomechanical consequences of foot deformities remain largely undocumented, we quantified the biomechanical alterations in loading at common running injury locations in individuals with hallux valgus during running.

Further, the assessment of tissue-specific fatigue-failure processes typically requires laboratory-based biomechanical measurements, which limits accessibility for wider use. To address this challenge, as a secondary aim, we developed a digital health framework that integrates wearable inertial sensor data with structure-aware fatigue modeling to estimate tissue-specific fatigue-failure risk in individuals with and without foot deformities. The developed model demonstrated strong performance in predicting fatigue-related outcomes using wearable-derived signals, suggesting potential for non-invasive and scalable injury risk estimation based on wearable data.

**5. Effect sizes for primary group comparisons**

**The manuscript reports p-values and mean ± SD for all discrete group comparisons but does not report effect sizes. Given the sample size of 26 per group and the number of outcomes examined, the practical magnitude of observed differences is essential for interpreting biomechanical relevance. Please add Cohen's d or Hedges' g with 95% confidence intervals for the primary group comparisons involving the fatigue indices (CL, CD, PFF) and the key biomechanical variables (peak ankle moment, peak MTP moment). These may be incorporated into the existing results tables or reported in a supplementary table.**

**Response:** We thank the reviewer for this helpful suggestion regarding the reporting of effect sizes. However, d values were already included in the original tables; however, we agree that providing the corresponding 95% confidence intervals improves the interpretability of the results. To address this, we have added the 95% confidence intervals for Cohen’s d for the primary group comparisons in a supplementary table, including the fatigue indices (CL, CD, PFF) and key biomechanical variables. We chose to present these results in the supplementary material to avoid overloading the main tables while ensuring full transparency and reproducibility.

**6. Data availability statement — URL error and scope clarification**

**The Figshare URL as written in the revised manuscript is https://10.6084/m9.figshare.31859476. This is a malformed address that will not resolve in a browser or citation manager.**

**Additionally, the data availability statement should be expanded to explicitly confirm whether the Figshare repository includes the pre-computed musculoskeletal simulation outputs — specifically the tissue force time-series and derived fatigue index values (CL, CD, PFF) per participant per trial. These simulation-derived values constitute the training targets for the deep learning model. A reader wishing to replicate the deep learning analysis without independently running the full OpenSim simulation pipeline would require access to these outputs. If they are present in the repository, please state this explicitly. If they are not currently deposited, they should be added, as they constitute part of the minimal dataset as defined by PLOS policy. Please also clarify in the cover letter whether trained model weights are included in the GitHub repository**

**Response:** We sincerely thank the reviewer for carefully identifying these issues related to data availability and reproducibility. We fully agree that clarity and accessibility of the shared resources are essential for ensuring transparency and replicability of the proposed framework.

First, we have corrected the Figshare repository URL in the revised manuscript. The previous link was incorrectly formatted. The correct and fully functional link is now: https://doi.org/10.6084/m9.figshare.31859476, which has been verified to resolve properly in both web browsers and citation managers. Second, we have substantially expanded the Data Availability Statement to clarify the contents of the Figshare repository explicitly. We confirm that the repository now includes not only raw experimental data but also the pre-computed musculoskeletal simulation outputs. These outputs correspond directly to the target variables used for training and validating the deep learning model.

By providing these processed simulation outputs, the repository enables replication of the machine learning analysis without requiring re-execution of the full OpenSim musculoskeletal simulation pipeline, thereby improving accessibility for readers who may not have access to specialized modeling environments. We appreciate the reviewer’s detailed and constructive feedback, which has significantly helped us improve the transparency and completeness of the data sharing components of this study.

**Revisions: Page 39 Lines 777-779**

All original data are available at <https://doi.org/10.6084/m9.figshare.31859476>.

**Minor Points**

**7. The capitalization of "Hallux valgus Angle (HVA)" is inconsistent in the abstract and should be standardized throughout. The baseline model performance presented in Supplementary Figures S6 and S7 should be accompanied by a tabulated numerical summary of R², RMSE, and NRMSE per model variant, per tissue site, and per fatigue index, as values cannot be extracted precisely from scatter plots. The absolute mean ± SD values of CL, CD, and PFF by group and tissue site should be presented in a main-text table to facilitate cross-study comparison. The discussion of mechanobiological pathways (Piezo1, TRPV4) added at R2 is currently too brief to add interpretive value; it should either be expanded with two to three sentences connecting the observed fatigue patterns to the cited signaling mechanisms, or removed.**

**Response:** We sincerely thank the reviewer for these thoughtful and constructive comments, which have helped us improve the clarity and completeness of the manuscript. In the revised version, we have carefully standardized the terminology of “hallux valgus angle (HVA)” throughout the abstract and main text to ensure consistency. In addition, to address the concern regarding the quantitative interpretation of model performance, we have supplemented the original figures with a detailed tabulated summary (Table Sx), reporting R², RMSE, and NRMSE for each model variant across different tissue sites and fatigue indices, thereby improving transparency and reproducibility. Regarding the discussion of mechanobiological pathways, we have revised this section by replacing the previous references with more directly relevant and up-to-date studies, thereby improving the alignment between the cited literature and the biomechanical findings of the present work. We appreciate the reviewer’s suggestions, which have significantly strengthened the rigor and presentation of our study.

**Revisions: Page 44 Lines 963-965**

**56.**	Ridge S T, Trotter T, Sponbeck J K, et al. Variability among individual Male runners influences cumulative loading more than foot strike type. Sports Health, 2026, 18(1): 214-221. <https://doi.org/10.1177/19417381251333415>

**Table S4**. Detailed results of the peak joint angle, moment, force and range of motion between the healthy and HV groups.

| Variables | | Health Group | HV Group | Difference 95% Confidence Interval | Mean Difference | Cohen’d Effect Size | P | Q |
| --- | --- | --- | --- | --- | --- | --- | --- | --- |
| Ankle | Maximal Dorsiflexion (°) | 20.52±5.87 | 18.22±4.51 | [1.02, 3.58] | 2.30 | 0.44 [-0.11, 0.99] | 0.011 | 0.020 |
|  | Maximal Plantarflexion (°) | -18.56±8.58 | -12.04±3.75 | [-8.14, -4.91] | -6.53 | 0.99 [0.41, 1.57] | 0.001 | 0.003 |
|  | Range of Motion (°) | 39.09±7.42 | 30.26±5.70 | [7.21, 10.44] | 8.83 | 1.34 [0.74, 1.94] | 0.001 | 0.003 |
|  | Peak Moment (N·m/kg) | -2.39±0.34 | -2.68±0.42 | [0.10, 0.48] | 0.29 | 0.83 [0.26, 1.40] | 0.004 | 0.009 |
| Knee | Maximal Flexion (°) | 46.65±4.08 | 44.10±5.87 | [-0.06, 5.17] | 2.55 | 0.50 [-0.05, 1.05] | 0.046 | 0.065 |
|  | Minimum Flexion (°) | 17.82±5.00 | 16.61±5.04 | [-1.35, 3.78] | 1.21 | 0.26 [-0.29, 0.81] | 0.073 | 0.093 |
|  | Range of Motion (°) | 28.82±3.22 | 27.49±3.54 | [-0.14, 2.82] | 1.34 | 0.51 [-0.04, 1.06] | 0.075 | 0.094 |
|  | Peak Moment (N·m/kg) | 3.44±0.56 | 3.59±0.51 | [-0.42, 0.12] | -0.15 | 0.31 [-0.24, 0.86] | 0.273 | 0.287 |

| Metatarsophalangeal | Maximal Dorsiflexion (°) | 36.30±5.38 | 29.92±4.49 | [5.17, 7.59] | 6.38 | 1.29 [0.69, 1.89] | 0.001 | 0.002 |
| --- | --- | --- | --- | --- | --- | --- | --- | --- |
|  | Minimum Dorsiflexion (°) | 9.01±2.31 | 7.53±1.47 | [1.01, 1.96] | 1.49 | 0.77 [0.21, 1.33] | 0.001 | 0.003 |
|  | Range of Motion (°) | 27.28±5.42 | 22.39±3.94 | [3.74, 6.05] | 4.89 | 1.03 [0.45, 1.61] | 0.001 | 0.001 |
|  | Peak Moment (N·m/kg) | -0.32±0.06 | -0.36±0.08 | [0.02, 0.06] | 0.04 | 0.80 [0.24, 1.37] | 0.003 | 0.007 |
| Subtalar | Maximal Dorsiflexion (°) | 8.67±1.39 | 8.33±1.27 | [0.01, 0.66] | 0.34 | 0.26 [-0.29, 0.81] | 0.042 | 0.066 |
|  | Minimum Dorsiflexion (°) | 0.18±0.60 | 0.29±0.50 | [-0.25, 0.02] | -0.11 | 0.20 [-0.35, 0.75] | 0.097 | 0.114 |
|  | Range of Motion (°) | 8.49±1.67 | 8.34±1.44 | [0.07, 0.83] | 0.45 | 0.10 [-0.44, 0.64] | 0.22 | 0.249 |
|  | Peak Moment (N·m/kg) | -1.19±0.17 | -1.27±0.17 | [0.03, 0.12] | 0.08 | 0.57 [0.02, 1.12] | 0.036 | 0.061 |
| Plantar fascia force | Peak Force (BW) | 2.83±0.34 | 3.01±0.34 | [-0.26, -0.08] | -0.17 | 0.71 [0.15, 1.27] | 0.001 | 0.002 |
| Achilles tendon force | Peak Force (BW) | 5.58±0.98 | 6.21±0.94 | [-0.87, -0.40] | -0.63 | 0.66 [0.10, 1.22] | 0.001 | 0.002 |
| Tibial impact force | Peak Force (BW) | 4.05±0.54 | 4.14±0.67 | [-0.29, 0.11] | -0.09 | 0.25 [-0.29, 0.80] | 0.383 | 0.396 |
| Patellofemoral force | Peak Force (BW) | 2.85±0.41 | 3.05±0.49 | [-0.33, -0.06] | -0.20 | 0.83 [0.26, 1.40] | 0.005 | 0.012 |

Note: “*” represents significance with p < 0.05.

**Table S5**. Detailed results of the CL, CD and PFF between the healthy and HV groups.

| Variables | | Health Group | HV Group | Difference 95% Confidence Interval | Mean Difference | Cohen’d Effect Size | P | | Q |
| --- | --- | --- | --- | --- | --- | --- | --- | --- | --- |
| Plantar fascia force | CL (N·s/km) | 192663.4±15645.6 | 208285.4±15212.3 | [6557.36, 25455.99] | -15622 | 0.92 [0.35, 1.49] | | **0.001** | 0.004 |
|  | CD (N·(s/km)^1/9.3^) | 3097.3±215.4 | 3286.9±181.6 | [12.83, 335.61] | -189.6 | 1.09 [0.50, 1.68] | | **0.035** | 0.060 |
|  | PFF (%) | 4.3±0.7 | 4.2±1.0 | [-0.063, 0.054] | 0.1 | 0.10 [-0.44, 0.64] | | 0.878 | 0.878 |
| Achilles tendon force | CL (N·s/km) | 325554.7±22473.9 | 363555.3±27435.2 | [63154.00, 89751.05] | -38000.6 | 1.59 [0.96, 2.22] | | **0.001** | 0.004 |
|  | CD (N·(s/km)^1/9.3^) | 5911.5±526.3 | 6584.0±700.7 | [310.72, 1065.11] | -672.5 | 0.99 [0.41, 1.57] | | **0.001** | 0.003 |
|  | PFF (%) | 4.5±0.9 | 5.2±1.2 | [0.0018, 0.1362] | -0.7 | 0.58 [0.03, 1.13] | | **0.044** | 0.066 |
| Tibial impact force | CL (N·s/km) | 287102.7±18526.3 | 298811.3±35630.6 | [-4423.93, 27840.95] | -11708.6 | 0.40 [-0.15, 0.95] | | 0.153 | 0.229 |
|  | CD (N·(s/km)^1/7^) | 5115.9±406.2 | 5265.7±571.0 | [-177.12, 422.93] | -149.8 | 0.28 [-0.27, 0.83] | | 0.415 | 0.498 |
|  | PFF (%) | 4.4±0.8 | 4.8±1.3 | [-0.02293, 0.10277] | -0.4 | 0.35 [-0.20, 0.90] | | 0.208 | 0.277 |
| Patellofemoral force | CL (N·s/km) | 134413.4±10544.2 | 144630.4±11779.9 | [3414.94, 16788.19] | -10217 | 0.85 [0.28, 1.42] | | **0.004** | 0.012 |
|  | CD (N·(s/km)^1/7^) | 3284.0±271.6 | 3513.6±240.9 | [-24.48, 460.59] | -229.6 | 0.53 [-0.02, 1.08] | | 0.077 | 0.103 |
|  | PFF (%) | 4.6±0.8 | 5.0±1.0 | [-0.0239, 0.0966] | -0.4 | 0.37 [-0.18, 0.92] | | 0.231 | 0.277 |

Note: CL: Cumulative load; CD: Cumulative damage; PFF: Probability of fatigue failure; “*” represents significance with p < 0.05.

**Table S6**. Detailed results of the correlation coefficients between HVA, FPI and CL, CD, PFF.

|  |  |  | | Health Group | | | | | | |  |  |  | HV Group | | | | | | | | | |
| --- | --- | --- | --- | --- | --- | --- | --- | --- | --- | --- | --- | --- | --- | --- | --- | --- | --- | --- | --- | --- | --- | --- | --- |
|  |  | HVA (9.9° ± 3.0°) | | | |  | FPI (1.8° ± 1.7°) | | | |  |  | HVA (23.8°±5.1°) | | | | |  | | FPI (5.9° ± 1.8°) | | | |
| Variables | | r | R^2^ | | P | Q | r | | R^2^ | P | Q | r | R^2^ | P | Q | r | | | R^2^ | | P | Q |  |
| Plantar fascia force | CL (N·s/km) | 0.39 | 0.1542 | | 0.0472 | 0.078 | | 0.23 | 0.055 | 0.2487 | 0.312 | 0.71 | 0.5064 | 0.0001 | <0.001 | | 0.66 | | 0.4299 | | 0.0001 | <0.001 |  |
|  | CD (N·(s/km)^1/9.3^) | 0.46 | 0.2124 | | 0.0178 | 0.034 | | 0.36 | 0.1279 | 0.0729 | 0.102 | 0.72 | 0.5115 | 0.0001 | <0.001 | | 0.61 | | 0.3768 | | 0.0002 | <0.001 |  |
|  | PFF (%) | 0.49 | 0.2373 | | 0.0116 | 0.023 | | 0.40 | 0.164 | 0.0401 | 0.061 | 0.65 | 0.4245 | 0.0002 | <0.001 | | 0.62 | | 0.3816 | | 0.0001 | <0.001 |  |
| Achilles tendon force | CL (N·s/km) | **0.79** | 0.6218 | | 0.0001 | <0.001 | | 0.60 | 0.3598 | 0.0012 | 0.003 | 0.83 | 0.6808 | 0.0001 | <0.001 | | 0.68 | | 0.4677 | | 0.0001 | <0.001 |  |
|  | CD (N·(s/km)^1/9.3^) | **0.75** | 0.5629 | | 0.0001 | <0.001 | | 0.56 | 0.3136 | 0.0029 | 0.006 | 0.85 | 0.7158 | 0.0001 | <0.001 | | 0.76 | | 0.5798 | | 0.0001 | <0.001 |  |
|  | PFF (%) | 0.66 | 0.4382 | | 0.0002 | <0.001 | | 0.58 | 0.342 | 0.0017 | 0.004 | 0.82 | 0.6775 | 0.0001 | <0.001 | | 0.74 | | 0.546 | | 0.0001 | <0.001 |  |
| Tibial impact force | CL (N·s/km) | 0.51 | 0.259 | | 0.0079 | 0.015 | | 0.26 | 0.0669 | 0.2019 | 0.236 | 0.47 | 0.2178 | 0.0089 | 0.017 | | 0.29 | | 0.0835 | | 0.227 | 0.259 |  |
|  | CD (N·(s/km)^1/7^) | 0.53 | 0.276 | | 0.0059 | 0.012 | | 0.26 | 0.0675 | 0.1999 | 0.234 | 0.46 | 0.2107 | 0.0094 | 0.018 | | 0.28 | | 0.0808 | | 0.219 | 0.251 |  |
|  | PFF (%) | 0.33 | 0.1078 | | 0.1014 | 0.129 | | 0.01 | 0.0002 | 0.9483 | 0.948 | 0.43 | 0.1836 | 0.0239 | 0.041 | | 0.27 | | 0.0738 | | 0.206 | 0.238 |  |
| Patellofemoral force | CL (N·s/km) | 0.59 | 0.3465 | | 0.0016 | 0.004 | | 0.58 | 0.3361 | 0.0019 | 0.004 | 0.69 | 0.4801 | 0.0001 | <0.001 | | 0.55 | | 0.3079 | | 0.0028 | 0.006 |  |
|  | CD (N·(s/km)^1/7^) | 0.61 | 0.3739 | | 0.0009 | 0.002 | | 0.56 | 0.3134 | 0.0029 | 0.006 | 0.61 | 0.3776 | 0.0003 | <0.001 | | 0.54 | | 0.2864 | | 0.0062 | 0.012 |  |
|  | PFF (%) | 0.64 | 0.4092 | | 0.0004 | 0.001 | | 0.59 | 0.3458 | 0.0016 | 0.004 | 0.59 | 0.3483 | 0.0007 | 0.002 | | 0.50 | | 0.2491 | | 0.0074 | 0.015 |  |

Note: PFF: CL: Cumulative load; CD: Cumulative damage; PFF: Probability of fatigue failure; “*” represents significance with p < 0

**Table S7**. The mean value and standard deviation of R^2^, RMSE, NRMSE from each model

| Models | | R^2^ | RMSE | NRMSE |
| --- | --- | --- | --- | --- |
| BiLSTM | Plantar fascia CD | 0.8247 (0.005) | 195.74 (109.01) | 0.45 (0.25) |
|  | Plantar fascia PFF | 0.8783 (0.006) | 0.97 (0.81) | 0.26 (0.22) |
|  | Achilles tendon CD | 0.8759 (0.008) | 433.34 (350.82) | 0.27 (0.22) |
|  | Achilles tendon PFF | 0.8417 (0.007) | 1.33 (1.25) | 0.26 (0.25) |
|  | Tibial CD | 0.8306 (0.004) | 340.74 (244.18) | 0.35 (0.24) |
|  | Tibial PFF | 0.8222 (0.006) | 1.12 (1.07) | 0.19 (0.18) |
|  | Patellofemoral CD | 0.8510 (0.007) | 236.17 (164.88) | 0.31 (0.21) |
|  | Patellofemoral PFF | 0.8370 (0.006) | 1.05 (1.04) | 0.23 (0.20) |
| CNN–BiLSTM | Plantar fascia CD | 0.8435 (0.005) | 167.45 (115.07) | 0.39 (0.27) |
|  | Plantar fascia PFF | 0.8565 (0.004) | 0.78 (0.57) | 0.21 (0.15) |
|  | Achilles tendon CD | 0.8894 (0.006) | 384.25 (273.79) | 0.24 (0.17) |
|  | Achilles tendon PFF | 0.8547 (0.005) | 1.06 (0.82) | 0.21 (0.16) |
|  | Tibial CD | 0.8465 (0.004) | 360.40 (285.55) | 0.37 (0.29) |
|  | Tibial PFF | 0.8540 (0.007) | 0.68 (0.49) | 0.11 (0.08) |
|  | Patellofemoral CD | 0.8767 (0.006) | 231.03 (175.68) | 0.31 (0.23) |
|  | Patellofemoral PFF | 0.8538 (0.005) | 1.11 (0.88) | 0.25 (0.20) |
| SSO–CNN–BiLSTM | Plantar fascia CD | 0.8768 (0.004) | 140.71 (110.01) | 0.33 (0.25) |
|  | Plantar fascia PFF | 0.8932 (0.002) | 0.72 (0.56) | 0.20 (0.15) |
|  | Achilles tendon CD | 0.9054 (0.005) | 375.51 (277.54) | 0.24 (0.18) |
|  | Achilles tendon PFF | 0.8757 (0.003) | 1.03 (0.91) | 0.20 (0.18) |
|  | Tibial CD | 0.8649 (0.005) | 261.67 (180.73) | 0.27 (0.19) |
|  | Tibial PFF | 0.9172 (0.005) | 0.52 (0.45) | 0.08 (0.07) |
|  | Patellofemoral CD | 0.8664 (0.002) | 215.82 (158.66) | 0.29 (0.21) |
|  | Patellofemoral PFF | 0.8891 (0.004) | 1.08 (0.81) | 0.24 (0.18) |
| SSO–CNN–BiLSTM-HAM | Plantar fascia CD | 0.8995 (0.003) | 133.47 (91.02) | 0.30 (0.21) |
|  | Plantar fascia PFF | 0.9269 (0.002) | 0.48 (0.45) | 0.13 (0.12) |
|  | Achilles tendon CD | 0.9324 (0.002) | 301.12 (213.98) | 0.19 (0.13) |
|  | Achilles tendon PFF | 0.9080 (0.003) | 0.64 (0.57) | 0.13 (0.11) |
|  | Tibial CD | 0.8896 (0.004) | 286.69 (197.23) | 0.30 (0.20) |
|  | Tibial PFF | 0.9302 (0.002) | 0.46 (0.37) | 0.08 (0.06) |
|  | Patellofemoral CD | 0.9081 (0.003) | 166.77 (109.21) | 0.22 (0.15) |
|  | Patellofemoral PFF | 0.8893 (0.004) | 0.61 (0.53) | 0.14 (0.12) |

Note: CD: Cumulative damage; PFF: Probability of fatigue failure
