# Supplementary material for: Structure-aware fatigue modeling in foot deformities: A digital health framework for tissue-specific running injury risk prediction using multi-modal data
Source: PLOS Digit Health. 2026 Jul 10;5(7):e0001537. doi: 10.1371/journal.pdig.0001537 (PMC13354006; doi:10.1371/journal.pdig.0001537)
Supplement: Peer review history

# Peer review: Decision Letter 0

**DOI:** 10.1371/journal.pdig.0001537.r001

**Authors:** Amara Tariq, Tomasz Cudejko

5 Mar 2026

PDIG-D-25-01162

Structure-Aware Fatigue Modeling in Foot Deformities: A Digital Health Framework for Tissue-Specific Risk Prediction Using Wearable Data

PLOS Digital Health

Dear Dr. Xu,

Thank you for submitting your manuscript to PLOS Digital Health. After careful consideration, we feel that it has merit but does not fully meet PLOS Digital Health's publication criteria as it currently stands. Therefore, we invite you to submit a revised version of the manuscript that addresses the points raised during the review process.

Please submit your revised manuscript by Apr 04 2026 11:59PM. If you will need more time than this to complete your revisions, please reply to this message or contact the journal office at digitalhealth@plos.org. When you're ready to submit your revision, log on to https://www.editorialmanager.com/pdig/ and select the 'Submissions Needing Revision' folder to locate your manuscript file.

Please include the following items when submitting your revised manuscript:

* A letter that responds to each point raised by the editor and reviewer(s). You should upload this letter as a separate file labeled 'Response to Reviewers'. This file does not need to include responses to any formatting updates and technical items listed in the 'Journal Requirements' section below.

* A marked-up copy of your manuscript that highlights changes made to the original version. You should upload this as a separate file labeled 'Revised Manuscript with Track Changes'.

* An unmarked version of your revised paper without tracked changes. You should upload this as a separate file labeled 'Manuscript'.

If you would like to make changes to your financial disclosure, competing interests statement, or data availability statement, please make these updates within the submission form at the time of resubmission. Guidelines for resubmitting your figure files are available below the reviewer comments at the end of this letter.

We look forward to receiving your revised manuscript.

Kind regards,

Tomasz Cudejko

Academic Editor

PLOS Digital Health

Amara Tariq

Section Editor

PLOS Digital Health

Leo Anthony Celi

Editor-in-Chief

PLOS Digital Health

orcid.org/0000-0001-6712-6626

**Journal Requirements:**

1. Please amend your detailed Financial Disclosure statement. This is published with the article. It must therefore be completed in full sentences and contain the exact wording you wish to be published.

i. Please clarify all sources of financial support for your study. List the grants, grant numbers, and organizations that funded your study, including funding received from your institution. Please note that suppliers of material support, including research materials, should be recognized in the Acknowledgements section rather than in the Financial Disclosure.

ii. State the initials, alongside each funding source, of each author to receive each grant. For example: "This work was supported by the National Institutes of Health (####### to AM; ###### to CJ) and the National Science Foundation (###### to AM)."

iii. State what role the funders took in the study. If the funders had no role in your study, please state: “The funders had no role in study design, data collection and analysis, decision to publish, or preparation of the manuscript.”

iv. If any authors received a salary from any of your funders, please state which authors and which funders.

2. Please ensure that your Ethics Statement is available in its entirety at the beginning of your Methods section, under a subheading 'Ethics Statement'. It must include:

i) The name(s) of the Institutional Review Board(s) or Ethics Committee(s)

ii) The approval number(s), or a statement that approval was granted by the named board(s)

iii) (for human participants or donors) - A statement that formal consent was obtained (must state whether verbal/written) OR the reason consent was not obtained (e.g., anonymity).

3. Please upload separate figure files in .tif or .eps format. Also, remove the figures from your manuscript file but keep the legends.

For more information about figure files please see our guidelines:

https://journals.plos.org/digitalhealth/s/figures

https://journals.plos.org/digitalhealth/s/figures#loc-file-requirements

4. We have noticed that you have uploaded Supporting Information files, but you have not included a list of legends. Please add a full list of legends for your Supporting Information files after the references list.

5. We note that your Data Availability Statement is currently as follows: The lower limb musculoskeletal models are available at https://simtk.org/projects/ligament_dct; https://simtk.org/projects/foot-anklemodel. All data relevant to the current study are included in the article and supplementary materials.

Please confirm at this time whether or not your submission contains all raw data required to replicate the results of your study. Authors must share the “minimal data set” for their submission. PLOS defines the minimal data set to consist of the data required to replicate all study findings reported in the article, as well as related metadata and methods (https://journals.plos.org/plosone/s/data-availability#loc-minimal-data-set-definition).

For example, authors should submit the following data:

- The values behind the means, standard deviations and other measures reported;

- The values used to build graphs;

- The points extracted from images for analysis.

Authors do not need to submit their entire data set if only a portion of the data was used in the reported study.

If your submission does not contain these data, please either upload them as Supporting Information files or deposit them to a stable, public repository and provide us with the relevant URLs, DOIs, or accession numbers. For a list of recommended repositories, please see https://journals.plos.org/plosone/s/recommended-repositories.

If there are ethical or legal restrictions on sharing a de-identified data set, please explain them in detail (e.g., data contain potentially sensitive information, data are owned by a third-party organization, etc.) and who has imposed them (e.g., an ethics committee). Please also provide contact information for a data access committee, ethics committee, or other institutional body to which data requests may be sent. If data are owned by a third party, please indicate how others may request data access.

6. Some material included in your submission may be copyrighted. According to PLOS’s copyright policy, authors who use figures or other material (e.g., graphics, clipart, maps) from another author or copyright holder must demonstrate or obtain permission to publish this material under the Creative Commons Attribution 4.0 International (CC BY 4.0) License used by PLOS journals. Please closely review the details of PLOS’s copyright requirements here: PLOS Licenses and Copyright. If you need to request permissions from a copyright holder, you may use PLOS's Copyright Content Permission form.

Please respond directly to this email or email the journal office and provide any known details concerning your material's license terms and permissions required for reuse, even if you have not yet obtained copyright permissions or are unsure of your material's copyright compatibility.

Potential Copyright Issues:

a. Figures 5, 6, 7: Please confirm whether you drew the images / clip-art within the figure panels by hand. If you did not draw the images, please provide (a) a link to the source of the images or icons and their license / terms of use; or (b) written permission from the copyright holder to publish the images or icons under our CC-BY 4.0 license. Alternatively, you may replace the images with open source alternatives. See these open source resources you may use to replace images / clip-art:

- https://commons.wikimedia.org

- https://openclipart.org/

If the reviewer comments include a recommendation to cite specific previously published works, please review and evaluate these publications to determine whether they are relevant and should be cited. There is no requirement to cite these works unless the editor has indicated otherwise.

**Additional Editor Comments (if provided):**

[Note: HTML markup is below. Please do not edit.]

**Reviewers' Comments:**

Reviewer's Responses to Questions

**Comments to the Author**

1. Does this manuscript meet PLOS Digital Health’s publication criteria? Is the manuscript technically sound, and do the data support the conclusions? The manuscript must describe methodologically and ethically rigorous research with conclusions that are appropriately drawn based on the data presented.

Reviewer #1: Partly

Reviewer #2: Yes

Reviewer #3: Yes

Reviewer #4: Partly

**********

2. Has the statistical analysis been performed appropriately and rigorously?

Reviewer #1: Yes

Reviewer #2: I don't know

Reviewer #3: Yes

Reviewer #4: No

**********

3. Have the authors made all data underlying the findings in their manuscript fully available (please refer to the Data Availability Statement at the start of the manuscript PDF file)?

The PLOS Data policy requires authors to make all data underlying the findings described in their manuscript fully available without restriction, with rare exception. The data should be provided as part of the manuscript or its supporting information, or deposited to a public repository. For example, in addition to summary statistics, the data points behind means, medians and variance measures should be available. If there are restrictions on publicly sharing data—e.g. participant privacy or use of data from a third party—those must be specified.

Reviewer #1: No

Reviewer #2: Yes

Reviewer #3: Yes

Reviewer #4: Yes

**********

4. Is the manuscript presented in an intelligible fashion and written in standard English?

PLOS Digital Health does not copyedit accepted manuscripts, so the language in submitted articles must be clear, correct, and unambiguous. Any typographical or grammatical errors should be corrected at revision, so please note any specific errors here.

Reviewer #1: Yes

Reviewer #2: Yes

Reviewer #3: No

Reviewer #4: Yes

**********

5. Review Comments to the Author

Please use the space provided to explain your answers to the questions above. You may also include additional comments for the author, including concerns about dual publication, research ethics, or publication ethics. (Please upload your review as an attachment if it exceeds 20,000 characters)

Reviewer #1: The manuscript addresses an important problem and presents a novel structure aware framework integrating musculoskeletal simulation with wearable driven prediction of tissue fatigue indices. The mechanistic modeling is a clear strength. However, the machine learning evaluation requires stronger rigor, including participant level validation, leakage safeguards, uncertainty reporting, baseline comparisons, and ablation studies. In addition, claims regarding real world wearable deployment should be tempered or supported with field robustness evidence. Finally, data and code availability should be strengthened to enable independent reproduction of analyses and reported performance.

Reviewer #2: Overall Summary:

This manuscript presents a comprehensive and methodologically sophisticated study integrating biomechanical modeling, wearable sensor data, and hierarchical deep learning to predict tissue-specific fatigue risk in runners with hallux valgus (HV). The work is timely, addresses a clear gap in the literature, and demonstrates high predictive accuracy. However, several limitations should be addressed to strengthen the manuscript.

Abstract

The abstract does not mention the sample size (n=26 per group) or acknowledge that the findings are based on a relatively small cohort, which may affect the generalizability of the proposed framework to broader populations.

Introduction

While the technical gaps in fatigue modeling are well articulated, the introduction could better frame the clinical need for such a model—specifically, how it would be integrated into existing prevention or rehabilitation pathways for runners with HV.

Methods

The manuscript mentions using IMU data from Xsens sensors but provides minimal detail on how these signals were preprocessed, synchronized, or validated against the gold-standard motion capture system. This omission makes it difficult to assess the reliability of the wearable data pipeline. Additonally, validity and relaibility of the sensors should be acknolwedged (https://www.nature.com/articles/s41598-022-18845-x)

The subject-specific model assumes consistent ligament properties across participants, which may not capture inter-individual variability in tissue mechanics. The use of a single constitutive model (NLSTV) for all ligaments may oversimplify the diverse viscoelastic behaviors of different connective tissues.

Results

While R² values are reported, other important metrics—such as mean absolute error (MAE), root mean square error (RMSE), or precision-recall for classification of high-risk individuals—are not provided. This limits the ability to fully evaluate model robustness

Discussion

The discussion strongly implies that HV causes increased fatigue risk, but the study design is cross-sectional and correlational. Causal claims should be tempered, and the potential for confounding factors (e.g., running experience, training load) should be acknowledged.

While the authors note that real-time field validation was not performed, the discussion does not sufficiently address the practical challenges of deploying such a model outside the lab—such as sensor placement variability, environmental noise, or user compliance.

Reviewer #3: This manuscript proposes a digital health framework combining gait analysis, nonlinear subject-specific musculoskeletal models, and hierarchical deep learning algorithms, aimed at quantifying and predicting tissue-specific fatigue risks in runners with Hallux Valgus (HV) compared to healthy controls during running. By integrating multi-joint coordination, muscle-tendon nonlinearity, and tissue-level cumulative damage mechanics, the authors reveal specific loading patterns in HV runners under foot structural deviations (e.g., increased hallux valgus angle and foot posture index), specifically showing significantly elevated risks of cumulative damage in the Achilles tendon and plantar fascia. This study provides a potential theoretical and technical pathway for personalized sports injury prevention and remote monitoring. However, there are some limitations in the manuscript that need further refinement.

Major Comments

1. The authors constructed a deep learning model (SSO-CNN-BILSTM-HAM) to predict fatigue trajectories; however, the sample size is relatively small (26 in the HV group and 26 in the control group). For deep learning methods, insufficient sample size may lead to model overfitting, thereby affecting its generalization ability. It is suggested that the authors further discuss the impact of small sample data on model stability, or attempt to use validation methods more suitable for small samples (such as leave-one-out cross-validation) to enhance the credibility of the results. Furthermore, although "Wearable Data" is mentioned in the title, the data for model training in the methodology section appears to be primarily derived from laboratory-grade gait analysis systems. The authors should clarify how high-dimensional data obtained solely from wearable devices in real-world application scenarios can meet the input requirements of this complex model, or provide supplementary validation analysis on the consistency between wearable sensor signals and laboratory gold-standard data.

2. Regarding biomechanical modeling, the authors used OpenSim for simulation. For Hallux Valgus, a deformity with significant skeletal structural changes, generic musculoskeletal models may not accurately reflect changes in joint moment arms and muscle lines of action. The authors should detail whether geometric scaling or adjustments specific to HV pathological features were performed on the model, and how these structural differences specifically affect the calculated internal loads. If no specific adjustments were made, the authors need to analyze in depth the potential errors introduced by model generalization in the discussion. Additionally, regarding the definition of "Cumulative Damage," which is currently based mainly on theoretical calculations using stress-life (S-N) curves, the authors should consider adding a discussion on the correlation between these theoretical calculations and actual clinical symptoms or radiographic evidence to enhance clinical translational significance.

3. The authors focused on the accumulation of mechanical loads in the discussion section. To improve the depth of the paper, it is suggested that the authors add an in-depth discussion exploring the potential biological mechanisms of tissue injury caused by abnormal mechanical load transmission, as well as strategies for handling small sample data in the digital health field. For example, mechanosensitive ion channels (such as Piezo1 and TRPV4) play a key role in translating mechanical signals into biochemical signals (such as inflammation or tissue degeneration), which is closely related to the fatigue failure mechanism mentioned in the text (doi: 10.2174/0118761429317745241017114020). Meanwhile, long-term abnormal mechanical stress may lead to muscle atrophy or remodeling, which is mutually causal with the pathological process of foot deformities and is worth supplementing from a pathophysiological perspective (doi: 10.1016/j.bcp.2022.114954).

Minor Comments

1. The manuscript requires professional native English editing to reduce grammatical errors and improve non-idiomatic expressions.

Reviewer #4: Major Comments

1. Multiple comparisons and SPM statistical control

The manuscript reports numerous significant time-window differences across multiple joints, variables (angles, moments, tissue forces), and outcome metrics using SPM and peak analyses.

Although SPM controls for temporal smoothness within a single waveform, the manuscript does not clearly describe how family-wise error was handled across multiple joints and biomechanical variables. Given the large number of parallel statistical tests, the absence of an explicit multiple comparison strategy may raise concerns about inflated type I error.

2. Correlation analyses without adjustment for confounders

Strong correlations are reported between HVA/FPI and CL/CD/PFF across tissues.

These correlations may be influenced by shared determinants such as running speed, cadence, body mass, BMI, training volume, or sex distribution. Without partial correlation or multivariable regression adjusting for potential confounders, the reported associations remain descriptive rather than mechanistically explanatory.

3. Inconsistency between CL/CD and PFF findings

In several tissues (e.g., plantar fascia, patellofemoral region), CL and CD are significantly elevated while PFF is not.

Since PFF is described as a fatigue failure probability derived from cumulative damage, a clearer explanation is needed regarding how PFF is computed and why statistical dissociation from CD may occur. Without methodological clarification of the fatigue model parameters and assumptions, interpretation of these discrepancies remains uncertain.

4. Extremely high R² values in deep learning models

Model 1 and Model 2 report R² values up to 0.94–0.97 for multiple fatigue indices.

Such high predictive performance in relatively small biomechanical datasets raises concerns about potential data leakage or overfitting. The manuscript does not clearly specify whether data partitioning was performed at the subject level or trial level. If multiple gait cycles from the same participant were included in both training and testing sets, performance metrics may be artificially inflated. Clear description of subject-level separation and independent validation is required.

5. Lack of external validation

Model performance appears to be evaluated within a single dataset.

Without external validation across independent cohorts, running speeds, or measurement systems, generalizability remains uncertain. The limitation section acknowledges lack of real-world validation, but the discussion of translational or scalable digital health implications appears stronger than the evidence currently supports.

6. Mechanistic interpretation exceeds presented analyses

The manuscript proposes a pathway from structural deviation (HV → pronation) to tissue-specific fatigue risk.

While group differences and correlations are demonstrated, formal mediation or pathway analysis linking HV severity, dynamic pronation measures, and fatigue indices is not shown. Therefore, mechanistic claims should be more cautiously framed as associative rather than causal.

7. DFIS validation requires quantitative detail

The manuscript states that model validation using DFIS showed strong agreement.

The number of participants validated, quantitative agreement metrics (e.g., RMSE, ICC), and validation scope (joint kinematics vs tissue forces vs fatigue indices) are not clearly detailed in the presented text. Without quantitative reporting, the strength of model validation cannot be fully assessed.

8. Sample size and model complexity balance

The study includes 26 HV and 26 controls, yet employs hierarchical deep learning architectures.

Given the relatively small subject number, justification for model complexity, overfitting prevention strategy, and effective sample size definition (subjects vs gait cycles) should be clearly described to ensure robustness.

9. Interpretation of sex-specific findings

Sex differences are described in subgroup analyses.

Given limited subgroup sizes, statistical power for sex-specific inference may be constrained. The manuscript should clarify whether analyses were pre-specified and whether interaction testing was performed.

10. Overextension of clinical translation claims

The manuscript suggests inclusion of structural markers into routine precision injury prevention frameworks.

Given the absence of prospective injury outcome data and lack of longitudinal validation, recommendations for clinical or remote monitoring implementation should be more conservatively stated.

The manuscript reports numerous significant time-window differences across multiple joints, variables (angles, moments, tissue forces), and outcome metrics using SPM and peak analyses.

Although SPM controls for temporal smoothness within a single waveform, the manuscript does not clearly describe how family-wise error was handled across multiple joints and biomechanical variables. Given the large number of parallel statistical tests, the absence of an explicit multiple comparison strategy may raise concerns about inflated type I error.

Minor Comments

11. Clarification of fatigue modeling parameters

The manuscript would benefit from explicit presentation of material constants, fatigue thresholds, and assumptions underlying the cumulative damage model to enhance reproducibility.

12. Reporting of uncertainty

Confidence intervals for effect sizes and predictive metrics are not consistently reported. Including interval estimates would strengthen statistical transparency.

13. Description of data normalization

Details regarding gait speed normalization, stride normalization, and signal preprocessing should be clearly summarized to facilitate replication.

14. Clarification of effective sample size

If multiple gait cycles per participant were used, the manuscript should explicitly state how statistical independence was ensured.

15. Manuscript formatting does not comply with journal requirements

The current version does not appear to follow the target journal’s formatting guidelines (structure, figure presentation, referencing style, and layout). Proper adherence to journal formatting standards is required prior to publication consideration.

**********

6. PLOS authors have the option to publish the peer review history of their article (what does this mean?). If published, this will include your full peer review and any attached files.

**Do you want your identity to be public for this peer review?**  If you choose “no”, your identity will remain anonymous but your review may still be made public.

For information about this choice, including consent withdrawal, please see our Privacy Policy.

Reviewer #1: No

Reviewer #2: No

Reviewer #3: No

Reviewer #4: No

**********

[NOTE: If reviewer comments were submitted as an attachment file, they will be attached to this email and accessible via the submission site. Please log into your account, locate the manuscript record, and check for the action link "View Attachments". If this link does not appear, there are no attachment files.]

**Figure resubmission:**

While revising your submission, we strongly recommend that you use PLOS’s NAAS tool (https://ngplosjournals.pagemajik.ai/artanalysis) to test your figure files. NAAS can convert your figure files to the TIFF file type and meet basic requirements (such as print size, resolution), or provide you with a report on issues that do not meet our requirements and that NAAS cannot fix.

After uploading your figures to PLOS’s NAAS tool - https://ngplosjournals.pagemajik.ai/artanalysis, NAAS will process the files provided and display the results in the "Uploaded Files" section of the page as the processing is complete. If the uploaded figures meet our requirements (or NAAS is able to fix the files to meet our requirements), the figure will be marked as "fixed" above. If NAAS is unable to fix the files, a red "failed" label will appear above. When NAAS has confirmed that the figure files meet our requirements, please download the file via the download option, and include these NAAS processed figure files when submitting your revised manuscript.

**Reproducibility:**

To enhance the reproducibility of your results, we recommend that authors of applicable studies deposit laboratory protocols in protocols.io, where a protocol can be assigned its own identifier (DOI) such that it can be cited independently in the future. Additionally, PLOS ONE offers an option to publish peer-reviewed clinical study protocols. Read more information on sharing protocols at https://plos.org/protocols?utm_medium=editorial-email&utm_source=authorletters&utm_campaign=protocols

Attachment Submitted filename: PDIG-D-25-01162_reviewer_JULIAN_BORGES.pdf

# Author comment: Author response to Decision Letter 1

**DOI:** 10.1371/journal.pdig.0001537.r002

28 Mar 2026

Attachment Submitted filename: Response to Editor and Reviewers.docx

# Peer review: Decision Letter 1

**DOI:** 10.1371/journal.pdig.0001537.r003

**Authors:** Amara Tariq, Tomasz Cudejko

20 Apr 2026

PDIG-D-25-01162R1

Structure-Aware Fatigue Modeling in Foot Deformities: A Digital Health Framework for Tissue-Specific Running Injury Risk Prediction Using Multi-Modal Data

PLOS Digital Health

Dear Dr. Xu,

Thank you for submitting your manuscript to PLOS Digital Health. After careful consideration, we feel that it has merit but does not fully meet PLOS Digital Health's publication criteria as it currently stands. Therefore, we invite you to submit a revised version of the manuscript that addresses the points raised during the review process.

Please submit your revised manuscript by Jun 19 2026 11:59PM. If you will need more time than this to complete your revisions, please reply to this message or contact the journal office at digitalhealth@plos.org. When you're ready to submit your revision, log on to https://www.editorialmanager.com/pdig/ and select the 'Submissions Needing Revision' folder to locate your manuscript file.

Please include the following items when submitting your revised manuscript:

* A letter that responds to each point raised by the editor and reviewer(s). You should upload this letter as a separate file labeled 'Response to Reviewers'. This file does not need to include responses to any formatting updates and technical items listed in the 'Journal Requirements' section below.

* A marked-up copy of your manuscript that highlights changes made to the original version. You should upload this as a separate file labeled 'Revised Manuscript with Track Changes'.

* An unmarked version of your revised paper without tracked changes. You should upload this as a separate file labeled 'Manuscript'.

If you would like to make changes to your financial disclosure, competing interests statement, or data availability statement, please make these updates within the submission form at the time of resubmission. Guidelines for resubmitting your figure files are available below the reviewer comments at the end of this letter.

We look forward to receiving your revised manuscript.

Kind regards,

Tomasz Cudejko

Academic Editor

PLOS Digital Health

Amara Tariq

Section Editor

PLOS Digital Health

Leo Anthony Celi

Editor-in-Chief

PLOS Digital Health

orcid.org/0000-0001-6712-6626

**Journal Requirements:**

If the reviewer comments include a recommendation to cite specific previously published works, please review and evaluate these publications to determine whether they are relevant and should be cited. There is no requirement to cite these works unless the editor has indicated otherwise.

**Additional Editor Comments (if provided):**

[Note: HTML markup is below. Please do not edit.]

**Reviewers' Comments:**

Reviewer's Responses to Questions

**Comments to the Author**

1. If the authors have adequately addressed your comments raised in a previous round of review and you feel that this manuscript is now acceptable for publication, you may indicate that here to bypass the “Comments to the Author” section, enter your conflict of interest statement in the “Confidential to Editor” section, and submit your "Accept" recommendation.

Reviewer #1: All comments have been addressed

Reviewer #2: All comments have been addressed

Reviewer #3: (No Response)

**********

2. Does this manuscript meet PLOS Digital Health’s publication criteria? Is the manuscript technically sound, and do the data support the conclusions? The manuscript must describe methodologically and ethically rigorous research with conclusions that are appropriately drawn based on the data presented.

Reviewer #1: Partly

Reviewer #2: Yes

Reviewer #3: (No Response)

**********

3. Has the statistical analysis been performed appropriately and rigorously?

Reviewer #1: (No Response)

Reviewer #2: I don't know

Reviewer #3: (No Response)

**********

4. Have the authors made all data underlying the findings in their manuscript fully available (please refer to the Data Availability Statement at the start of the manuscript PDF file)?

The PLOS Data policy requires authors to make all data underlying the findings described in their manuscript fully available without restriction, with rare exception. The data should be provided as part of the manuscript or its supporting information, or deposited to a public repository. For example, in addition to summary statistics, the data points behind means, medians and variance measures should be available. If there are restrictions on publicly sharing data—e.g. participant privacy or use of data from a third party—those must be specified.

Reviewer #1: Yes

Reviewer #2: Yes

Reviewer #3: (No Response)

**********

5. Is the manuscript presented in an intelligible fashion and written in standard English?

PLOS Digital Health does not copyedit accepted manuscripts, so the language in submitted articles must be clear, correct, and unambiguous. Any typographical or grammatical errors should be corrected at revision, so please note any specific errors here.

Reviewer #1: No

Reviewer #2: Yes

Reviewer #3: (No Response)

**********

6. Review Comments to the Author

Please use the space provided to explain your answers to the questions above. You may also include additional comments for the author, including concerns about dual publication, research ethics, or publication ethics. (Please upload your review as an attachment if it exceeds 20,000 characters)

**Reviewer #1:**  Summary Assessment

Thank you for the substantive revisions submitted in response to the first-round review. The authors have made meaningful progress across several dimensions. Subject-level data partitioning is now explicitly described in the Methods, directly addressing the information leakage concern raised at R1. The DFIS-based musculoskeletal model validation has been strengthened with quantitative agreement metrics (ICC 0.965–0.985 across tibiotalar displacement planes). Baseline model ablation comparisons have been added to the supplementary materials. The laboratory context of the IMU data collection is now correctly characterized throughout the Methods and Limitations sections. Causal language in the Discussion and Results has been appropriately moderated. The Associate Editor's formatting requirements — including the ethics statement, financial disclosure, figure filing, and data availability statement — have all been addressed. These revisions represent a genuine improvement in methodological transparency and reporting quality.

However, several concerns remain that must be resolved before the manuscript can be accepted for publication. Three issues are classified as mandatory corrections. Three additional concerns require author attention at the level of either targeted analysis or explicit inferential restriction.

Mandatory Corrections

1. Multiple comparison correction for discrete biomechanical variables — method not specified

The revised Methods section states that "when multiple comparisons were performed across related variables, appropriate adjustments were applied to control the type I error rate." This statement is procedurally insufficient for a journal committed to reproducible research. The manuscript reports independent-sample t-tests across multiple discrete outcome domains simultaneously: anatomical indices (HVA, IMA, FPI), joint kinematics across multiple planes and joints, joint kinetics, range of motion variables, and fatigue indices (CL, CD, PFF) at four tissue sites. The reader cannot assess whether the type I error rate was adequately controlled without knowing which correction procedure was applied, which tests were grouped into a comparison family, how many tests were included in each family, and what the corrected alpha threshold or adjusted p-values were for each domain. Please specify the correction method (e.g., Bonferroni, Holm-Bonferroni, Benjamini-Hochberg FDR) in the Statistical Analysis subsection of the Methods, state the number of comparisons in each family, and report the corrected significance thresholds. This information should also be reflected in the Results tables where applicable.

2. Correlation analyses between structural predictors and fatigue indices — unadjusted for confounders

The Pearson correlation and linear regression analyses between HVA/FPI and the fatigue indices (CL, CD, PFF) remain unadjusted for potential confounders. The authors acknowledge this limitation in the revised Discussion but characterize it as a future direction. We consider this an insufficient resolution for the following reason: body mass (65.2 ± 11.8 kg versus 67.3 ± 9.4 kg) and weekly mileage (18.5 ± 7.3 km versus 15.8 ± 6.8 km) differ between groups with substantial within-group variance, both variables were measured, and both could plausibly influence CL, CD, and PFF independently of foot morphology. Running speed was controlled within a narrow range but the actual inter-participant variance in speed is not reported and was not included as a covariate. Two paths to resolution are acceptable. The preferred path is to conduct partial correlation or simple multivariable regression analyses between HVA/FPI and the primary fatigue outcomes (at minimum for Achilles tendon and plantar fascia CL and CD) adjusting for body mass and running speed as covariates, and to report the adjusted coefficients alongside unadjusted Pearson r. The alternative path, if reanalysis is not possible, is to substantially restrict the inferential language associated with all HVA/FPI correlation findings in the Results and Discussion so that they are explicitly characterized as unadjusted bivariate associations subject to potential confounding, and to remove any mechanistic interpretation built directly on these correlations.

3. Reference list — reference 55 absent

The reference list in the revised manuscript jumps from reference 54 (Wren et al., Clinical Biomechanics, 2001) to reference 56 (Jia et al., Current Molecular Pharmacology, 2024). Reference 55 is absent. Please audit the full reference list against all in-text citations, identify the intended reference 55, restore it to the list, and verify that all subsequent citation numbers are correct. A note in the cover letter specifying the nature of the error would assist the editorial office.

Additional Required Revisions

4. Author summary — real-world monitoring language

The author summary states that the proposed framework enables "simple, non-invasive, and continuous monitoring outside specialized labs" and provides "individualized, real-time fatigue assessment" with potential for "large-scale screening in community and clinical settings." These claims are inconsistent with the characterization now present throughout the revised manuscript body, where the study is correctly described as a laboratory-validated proof-of-concept in which IMU signals were collected under controlled laboratory conditions synchronized with motion-capture instrumentation. The author summary is published alongside the article and is read by non-specialist audiences. Please revise it to accurately reflect the laboratory context of the data and the proof-of-concept status of the framework.

5. Effect sizes for primary group comparisons

The manuscript reports p-values and mean ± SD for all discrete group comparisons but does not report effect sizes. Given the sample size of 26 per group and the number of outcomes examined, the practical magnitude of observed differences is essential for interpreting biomechanical relevance. Please add Cohen's d or Hedges' g with 95% confidence intervals for the primary group comparisons involving the fatigue indices (CL, CD, PFF) and the key biomechanical variables (peak ankle moment, peak MTP moment). These may be incorporated into the existing results tables or reported in a supplementary table.

6. Data availability statement — URL error and scope clarification

The Figshare URL as written in the revised manuscript is https://10.6084/m9.figshare.31859476. This is a malformed address that will not resolve in a browser or citation manager.

Additionally, the data availability statement should be expanded to explicitly confirm whether the Figshare repository includes the pre-computed musculoskeletal simulation outputs — specifically the tissue force time-series and derived fatigue index values (CL, CD, PFF) per participant per trial. These simulation-derived values constitute the training targets for the deep learning model. A reader wishing to replicate the deep learning analysis without independently running the full OpenSim simulation pipeline would require access to these outputs. If they are present in the repository, please state this explicitly. If they are not currently deposited, they should be added, as they constitute part of the minimal dataset as defined by PLOS policy. Please also clarify in the cover letter whether trained model weights are included in the GitHub repository.

Minor Points

The capitalization of "Hallux valgus Angle (HVA)" is inconsistent in the abstract and should be standardized throughout. The baseline model performance presented in Supplementary Figures S6 and S7 should be accompanied by a tabulated numerical summary of R², RMSE, and NRMSE per model variant, per tissue site, and per fatigue index, as values cannot be extracted precisely from scatter plots. The absolute mean ± SD values of CL, CD, and PFF by group and tissue site should be presented in a main-text table to facilitate cross-study comparison. The discussion of mechanobiological pathways (Piezo1, TRPV4) added at R2 is currently too brief to add interpretive value; it should either be expanded with two to three sentences connecting the observed fatigue patterns to the cited signaling mechanisms, or removed.

**Reviewer #2:**  Thank you, all comments have been addressed.

**Reviewer #3:**  The latest revisions meet all the requirements set out in the previous feedback. This manuscript is now ready for acceptance in its current form.

**********

7. PLOS authors have the option to publish the peer review history of their article (what does this mean?). If published, this will include your full peer review and any attached files.

**Do you want your identity to be public for this peer review?**  If you choose “no”, your identity will remain anonymous but your review may still be made public.

For information about this choice, including consent withdrawal, please see our Privacy Policy.

Reviewer #1: No

Reviewer #2: No

Reviewer #3: None

**********

[NOTE: If reviewer comments were submitted as an attachment file, they will be attached to this email and accessible via the submission site. Please log into your account, locate the manuscript record, and check for the action link "View Attachments". If this link does not appear, there are no attachment files.]

**Figure resubmission:**

While revising your submission, we strongly recommend that you use PLOS’s NAAS tool (https://ngplosjournals.pagemajik.ai/artanalysis) to test your figure files. NAAS can convert your figure files to the TIFF file type and meet basic requirements (such as print size, resolution), or provide you with a report on issues that do not meet our requirements and that NAAS cannot fix.

After uploading your figures to PLOS’s NAAS tool - https://ngplosjournals.pagemajik.ai/artanalysis, NAAS will process the files provided and display the results in the "Uploaded Files" section of the page as the processing is complete. If the uploaded figures meet our requirements (or NAAS is able to fix the files to meet our requirements), the figure will be marked as "fixed" above. If NAAS is unable to fix the files, a red "failed" label will appear above. When NAAS has confirmed that the figure files meet our requirements, please download the file via the download option, and include these NAAS processed figure files when submitting your revised manuscript.

**Reproducibility:**

To enhance the reproducibility of your results, we recommend that authors of applicable studies deposit laboratory protocols in protocols.io, where a protocol can be assigned its own identifier (DOI) such that it can be cited independently in the future. Additionally, PLOS ONE offers an option to publish peer-reviewed clinical study protocols. Read more information on sharing protocols at https://plos.org/protocols?utm_medium=editorial-email&utm_source=authorletters&utm_campaign=protocols

Attachment Submitted filename: PDIG-D-25-01162R1_reviewer_JULIAN_BORGES.pdf

# Author comment: Author response to Decision Letter 2

**DOI:** 10.1371/journal.pdig.0001537.r004

11 May 2026

Attachment Submitted filename: Response to Editor and Reviewers R1.docx

# Editor report: Decision Letter 2

**DOI:** 10.1371/journal.pdig.0001537.r005

**Authors:** Amara Tariq, Tomasz Cudejko

15 Jun 2026

Structure-Aware Fatigue Modeling in Foot Deformities: A Digital Health Framework for Tissue-Specific Running Injury Risk Prediction Using Multi-Modal Data

PDIG-D-25-01162R2

Dear Xu,

We are pleased to inform you that your manuscript 'Structure-Aware Fatigue Modeling in Foot Deformities: A Digital Health Framework for Tissue-Specific Running Injury Risk Prediction Using Multi-Modal Data' has been provisionally accepted for publication in PLOS Digital Health.

Before your manuscript can be formally accepted you will need to complete some formatting changes, which you will receive in a follow-up email from a member of our team.

Please note that your manuscript will not be scheduled for publication until you have made the required changes, so a swift response is appreciated.

IMPORTANT: The editorial review process is now complete. PLOS will only permit corrections to spelling, formatting or significant scientific errors from this point onwards. Requests for major changes, or any which affect the scientific understanding of your work, will cause delays to the publication date of your manuscript.

If your institution or institutions have a press office, please notify them about your upcoming paper to help maximize its impact. If they'll be preparing press materials, please inform our press team as soon as possible -- no later than 48 hours after receiving the formal acceptance. Your manuscript will remain under strict press embargo until 2 pm Eastern Time on the date of publication. For more information, please contact digitalhealth@plos.org.

Thank you again for supporting Open Access publishing; we are looking forward to publishing your work in PLOS Digital Health.

Best regards,

Tomasz Cudejko, Ph.D.

Academic Editor

PLOS Digital Health

***********************************************************

**Additional Editor Comments (if provided):**

**Reviewer Comments (if any, and for reference):**
